# Supplementary material for: miR-146a mimic therapy protects against platelet-mediated thrombosis
Source: Mol Ther. 2026 Jul 13;34(9):5333–51. doi: 10.1016/j.ymthe.2026.07.019 (PMC13555580; doi:10.1016/j.ymthe.2026.07.019)
Supplement: Supplementary file 1 [file mmc1.pdf]

YMTHE, Volume *34*

Supplemental Information

miR-146a mimic therapy protects against platelet-mediated thrombosis

Sonia Águila, Juan C. García-Cañaveras, Laura Zapata-Martínez, Ana B. Arroyo, Nuria García-Barberá, Salvador Carrillo-Tornel, Pedro J. Guijarro-Carrillo, Paula González-Jiménez, Ana Latorre, María L. Lozano, Bernard Payrastre, José Rivera, Marie-Pierre Gratacap, Raúl Méndez, Agustín Lahoz, Marcin Kortylewski, Rocío González-Conejero, and Constantino Martínez

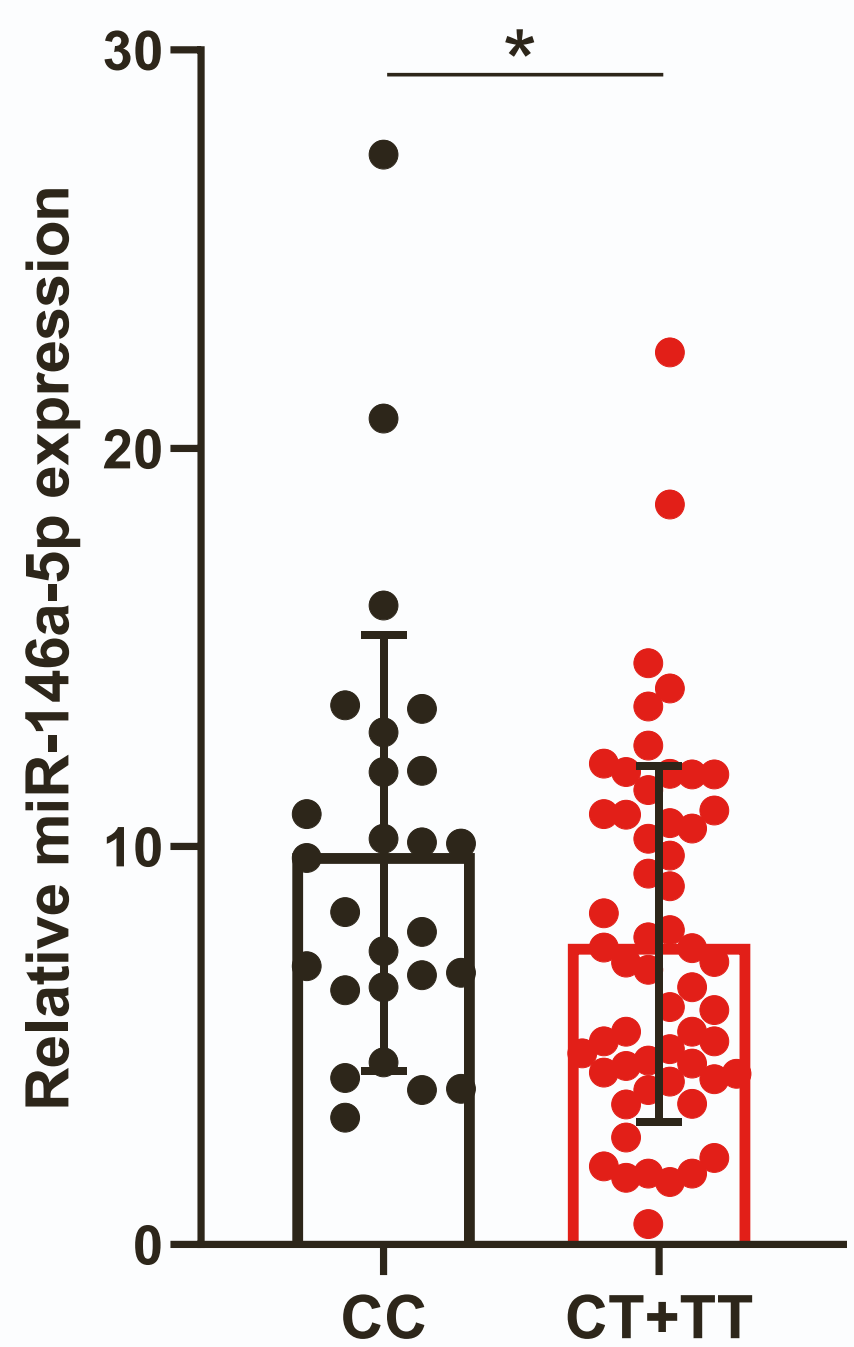

Figure S1. The presence of T-allele of rs2431697 decreased miR-146a in human platelets. RNA was extracted from washed platelets of 82 healthy donors' blood samples (CC=26, CT=26, TT=30). miR-146a were quantified by RT-qPCR. The statistical significance was calculated by Mann Whitney U test (*p<0.05).

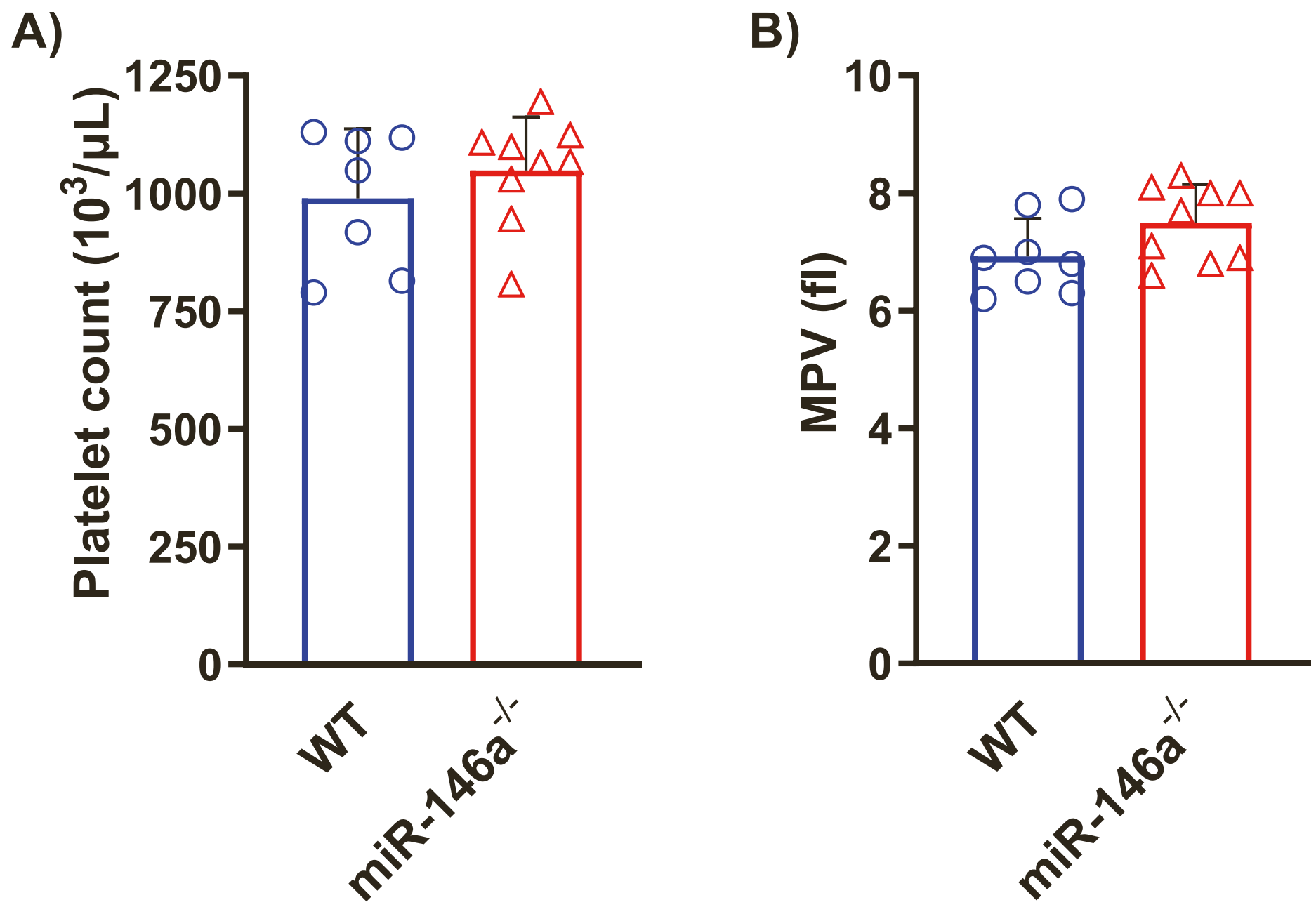

Figure S2. No significant differences in platelet count or volume between WT and miR-146a deficient mice. A) Platelet count and B) mean platelet volume (MPV) were measured (ADVIA 120 Hematology system) in 10 weeks-old mice (n=8 per group).

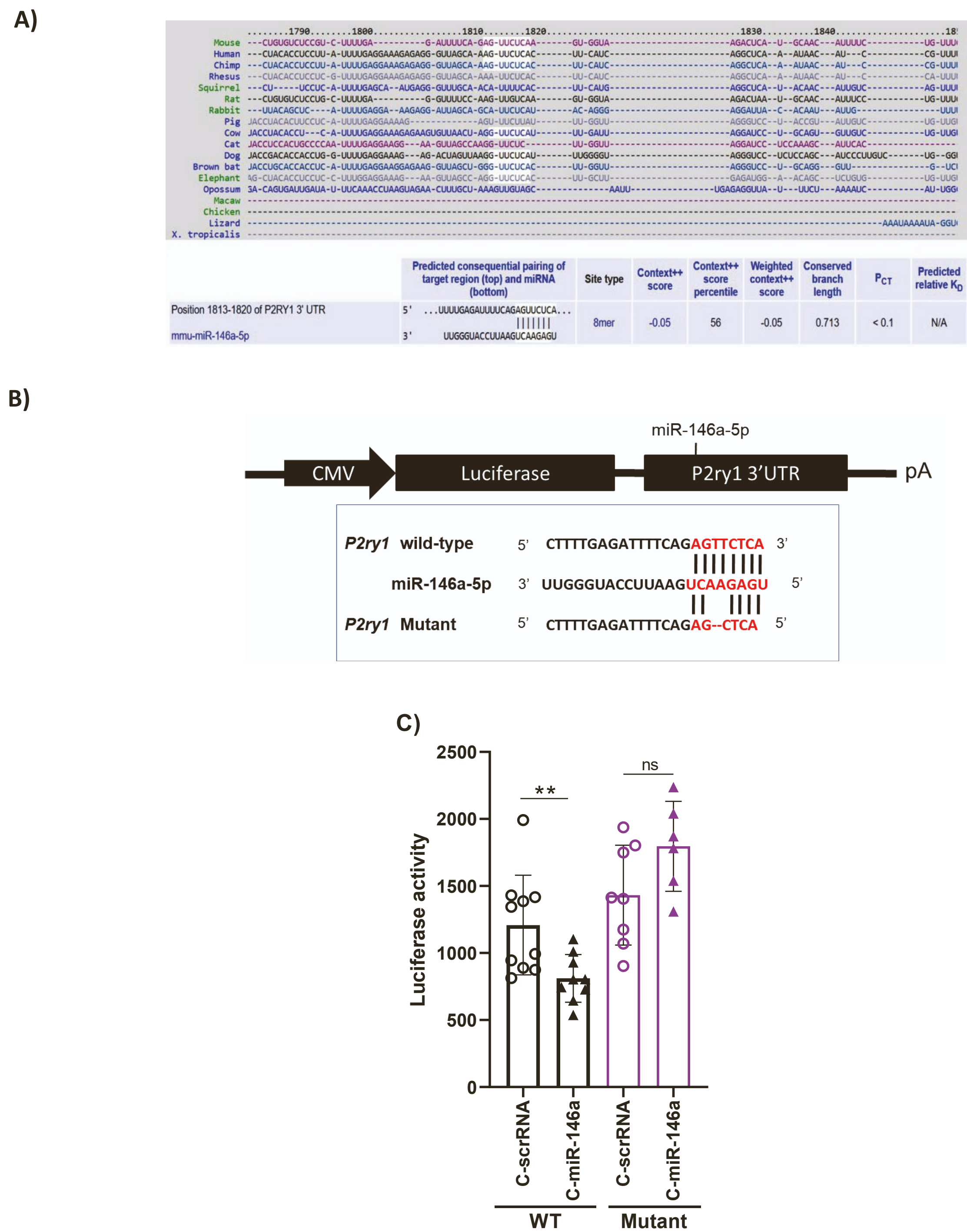

Figure S3. *P2ry1* gene is target of miR-146a. A) *P2ry1* mRNA is an *in-silico* target of miR-146a. TargetScanHuman software (https://www.targetscan.org/vert_80/) predicts that nucleotides (8mer) of 3'UTR region of *P2ry1* mRNA paired to miR-146a. B) Schematic diagram of the luciferase reporter plasmids containing a 390-nt fragment of the *P2ry1* wild-type (WT) 3'UTR with the predicted conserved miR-146a binding site, or a mutant *P2ry1* 3'UTR in which two nucleotides within the miR-146a-5p seed-matching region were deleted. C) Luciferase activity in HEK-293T cells co-transfected with the different vectors and a pRL-TK vector to normalize the data, and C-miR-146a mimic (C-miR-146a) or scramble RNA control (C-scrRNA) [n=2, 3-5 replicates/experiment]. The statistical significance was calculated by 2-tailed *t*-test (**p<0.01).

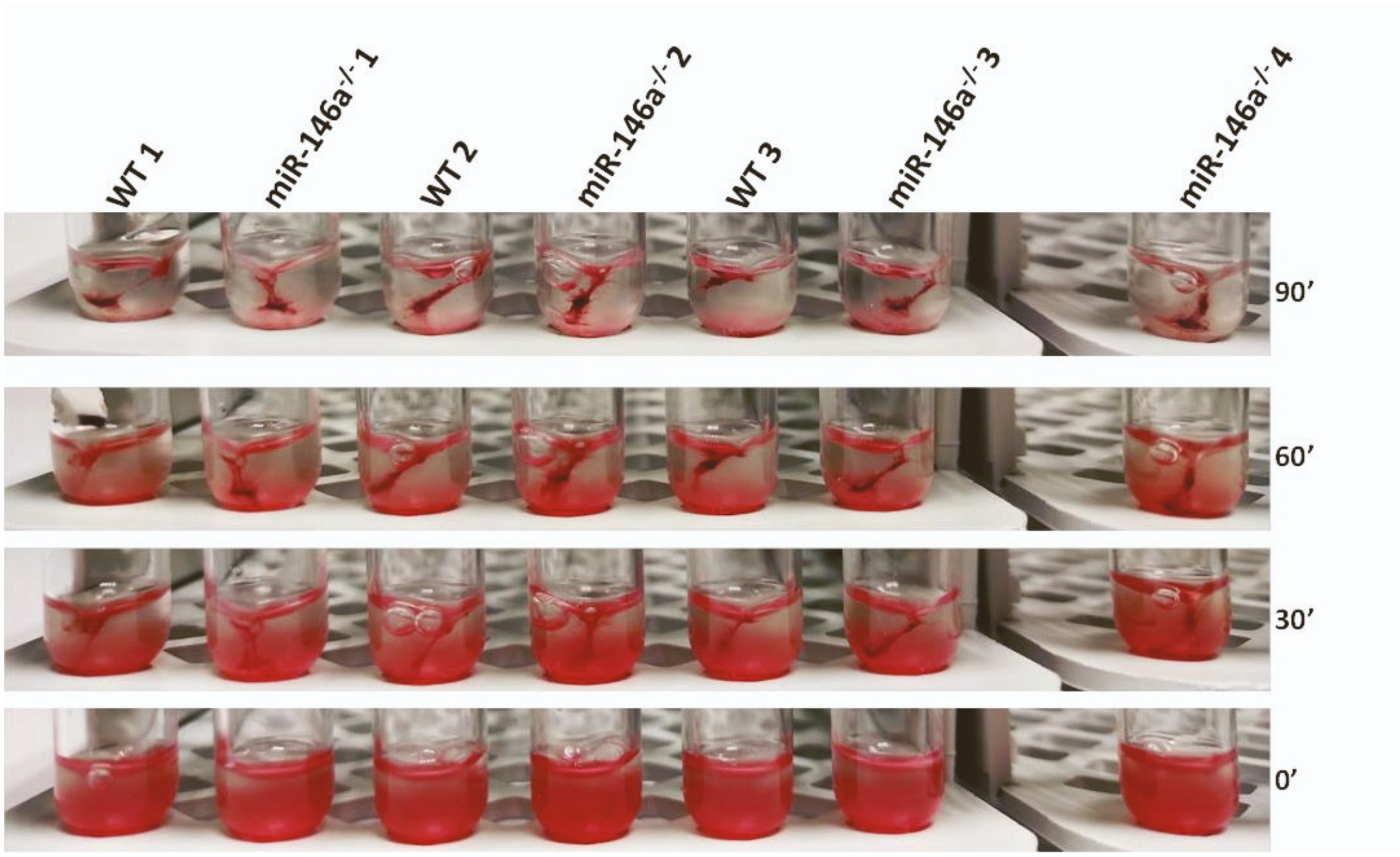

Figure S4. Clot retraction assay in WT *vs.* miR-146a$^{-/-}$ mice. $4x10^8$ washed platelets/mL were incubated in the presence of fibrinogen (2mg/mL) and thrombin (0.5 IU/mL) in Tyrode’s buffer + $CaCl_2$ (2 mM) during 90 min.

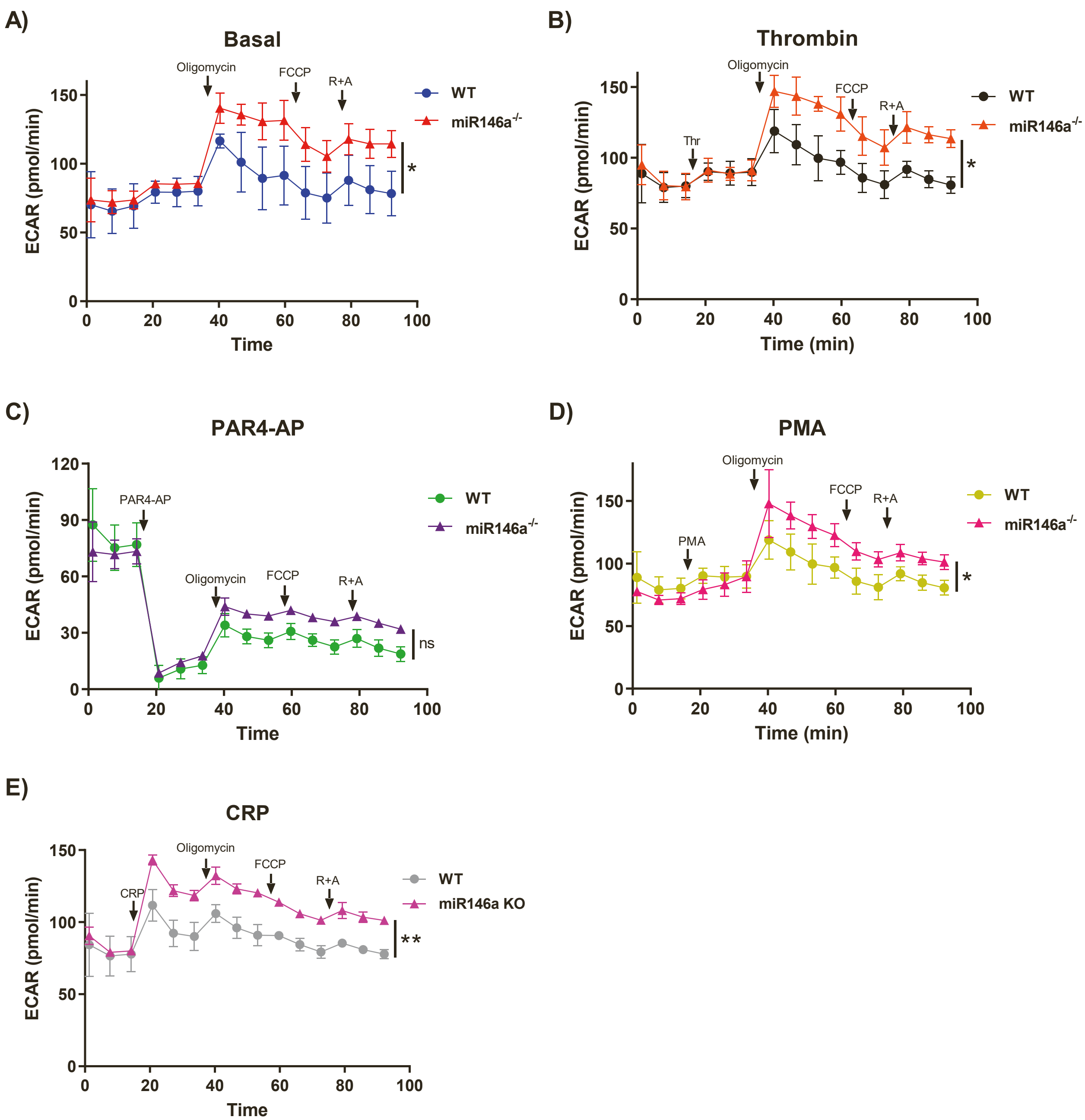

Figure S5. miR-146a$^{-/-}$ platelet showed elevated ECAR in resting and stimulated conditions. Extracellular acidification rate (ECAR) was determined by Seahorse Analyzer using Seahorse Cell Mito Stress Test kit at A) basal and (B-E) activated state with B) Thrombin (0.5IU/mL), C) PAR4-AP (500µM), D) PMA (30nM), E) CRP (10µg/mL) (pool of 6 mice/group, mean ± SD). The area under the curve (AUC) and its significance were calculated by 2-tailed *t*-test (*p<0.05, **p<0.01, ns=non-significant).

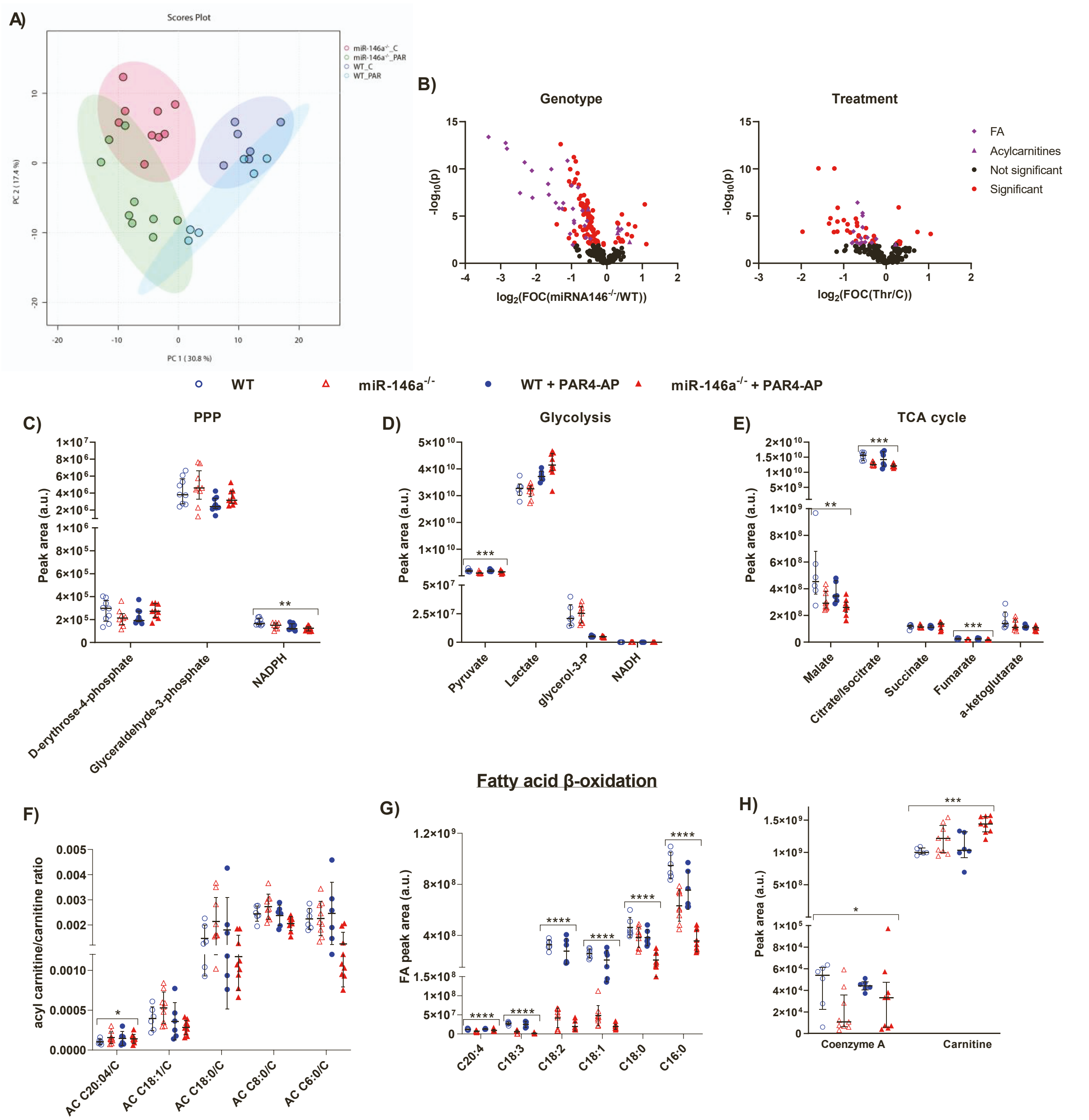

Figure S6. Metabolomics showed enhanced fatty acid β-oxidation (FAO) for miR-146a deficient platelets ± PAR4-AP. Untargeted metabolomics was performed in WT *vs*. miR-146a$^{-/-}$ platelets ± agonist 500µM PAR4-AP by Q Exactive Plus Hybrid Quadrupole-Orbitrap mass spectrometer (2-3 pools of 3 mice/group and technical triplicated). A) Principal component analysis (PCA), B) volcano plot of platelet metabolome. Main intermediates or products of C) pentose phosphate pathway (PPP), D) glycolysis, E) tricarboxylic acid cycle (TCA), F) acylcarnitines/carnitines (AC/C) ratio, G) fatty acid levels, H) coenzyme A and carnitines levels in resting or stimulated (Thr) platelets. The statistical significance was calculated by 2-way ANOVA *p<0.05; **p<0.01; ***p<0.001; ****p<0.0001, only the genotype statistical significance was represented in the graphs.

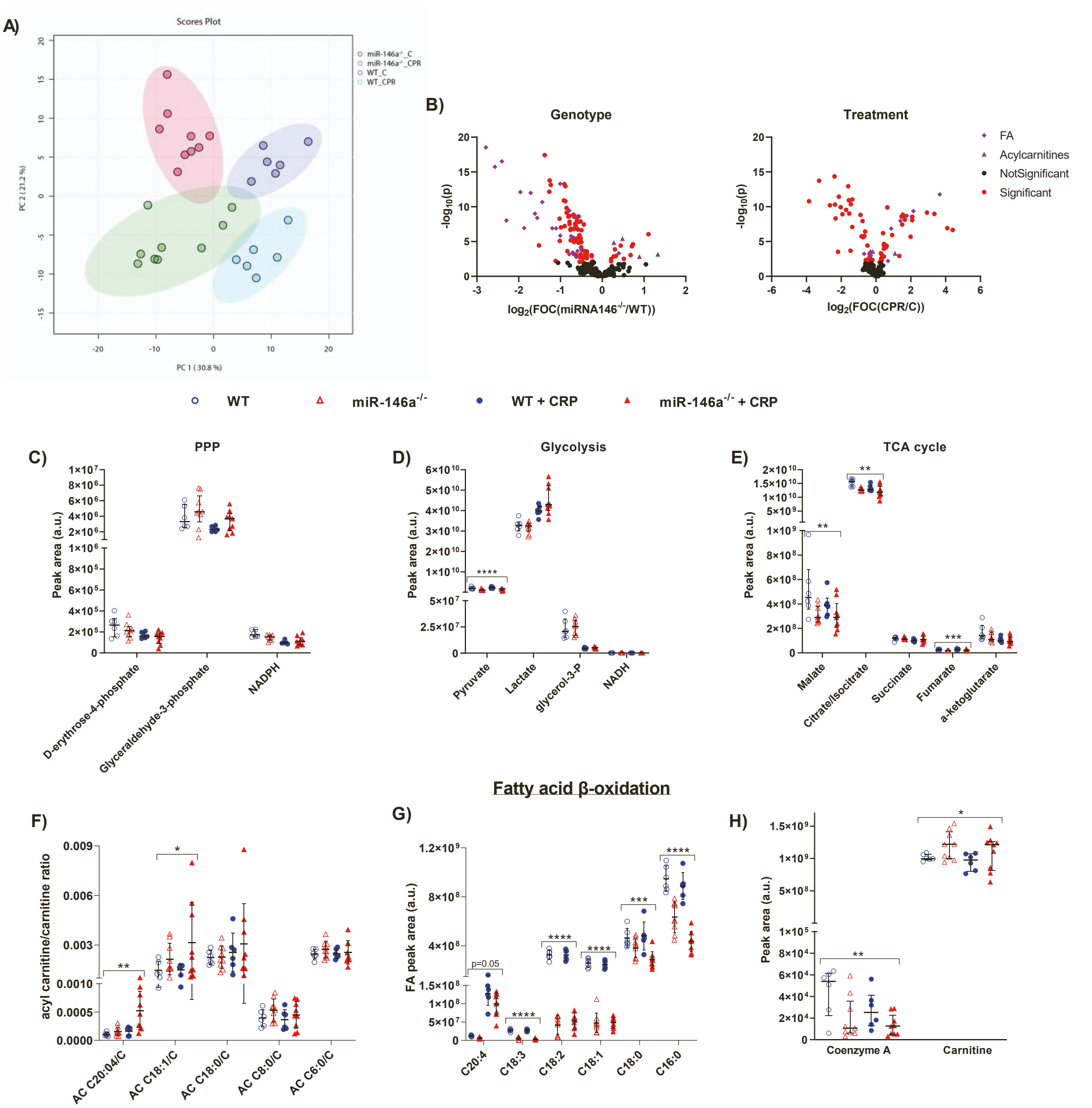

Figure S7. Metabolomics showed enhanced fatty acid β-oxidation for miR-146a deficient platelets ± CRP. Untargeted metabolomics was performed in WT *vs*. miR-146a$^{-/-}$ platelets ± agonist 10µg/mL CRP by Q Exactive Plus Hybrid Quadrupole-Orbitrap mass spectrometer (2-3 pools of 3 mice/group and technical triplicated). A) Principal component analysis (PCA), B) volcano plot of platelet metabolome. Main intermediates or products of C) pentose phosphate pathway (PPP), D) glycolysis and E) tricarboxylic acid cycle (TCA), F) acylcarnitines/carnitines (AC/C) ratio, G) fatty acid levels, H) coenzyme A and carnitines levels in resting or stimulated (Thr) platelets. The statistical significance was calculated by 2-way ANOVA *p<0.05; **p<0.01; ***p<0.001; ****p<0.0001, only the genotype statistical significance was represented in the graphs.

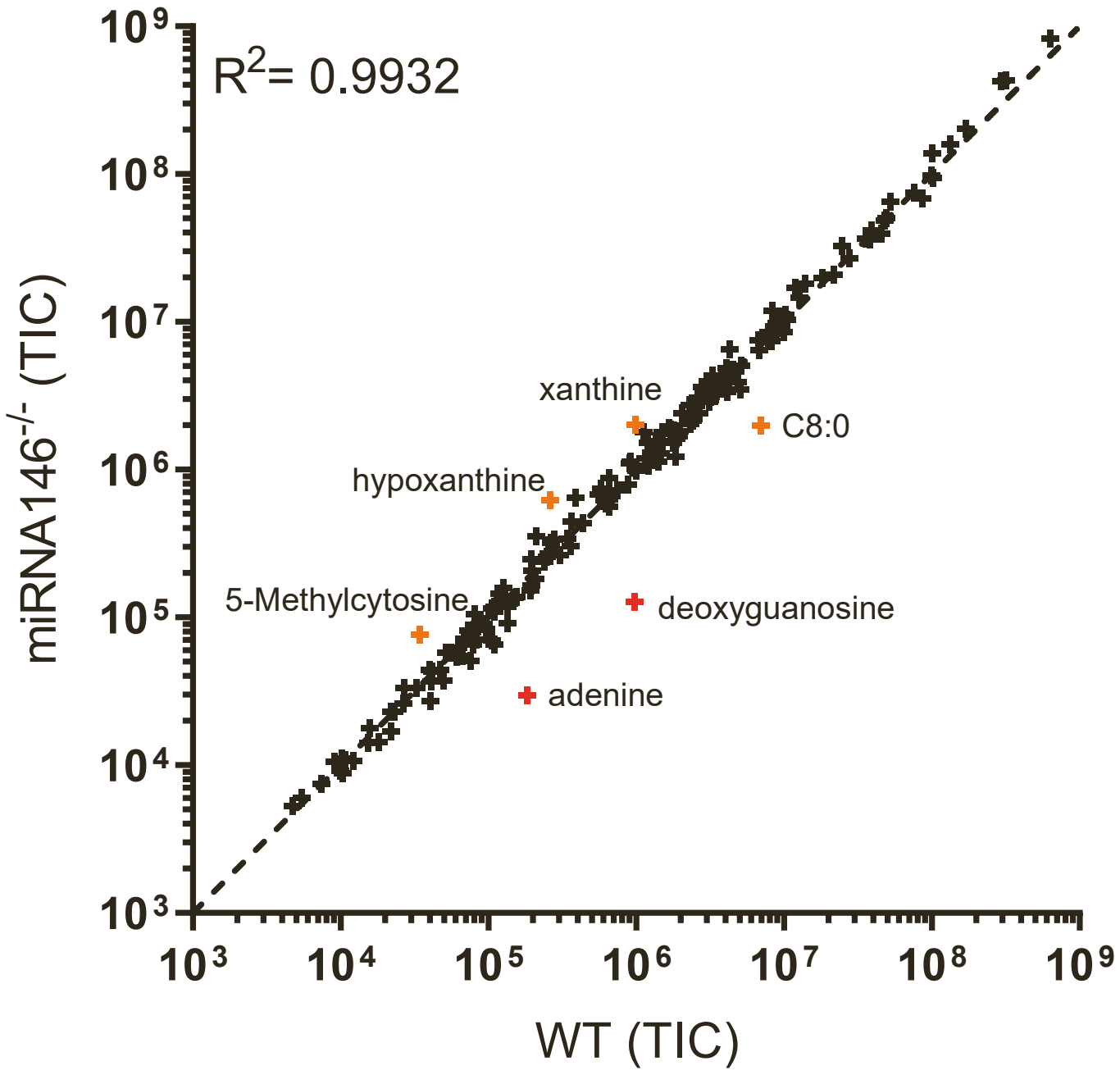

Figure S8. Serum metabolome profiles from WT and miR-146a$^{-/-}$ mice were similar. Untargeted metabolomics was performed in WT *vs.* miR-146a$^{-/-}$ serum by Quadrupole-Q Exactive Orbitrap mass spectrometer (mean, n = 6/group**).** Metabolites whose abundances differ by more than four-fold between conditions are highlighted in red, metabolites whose abundances differ by more than two-fold between conditions are highlighted in orange.

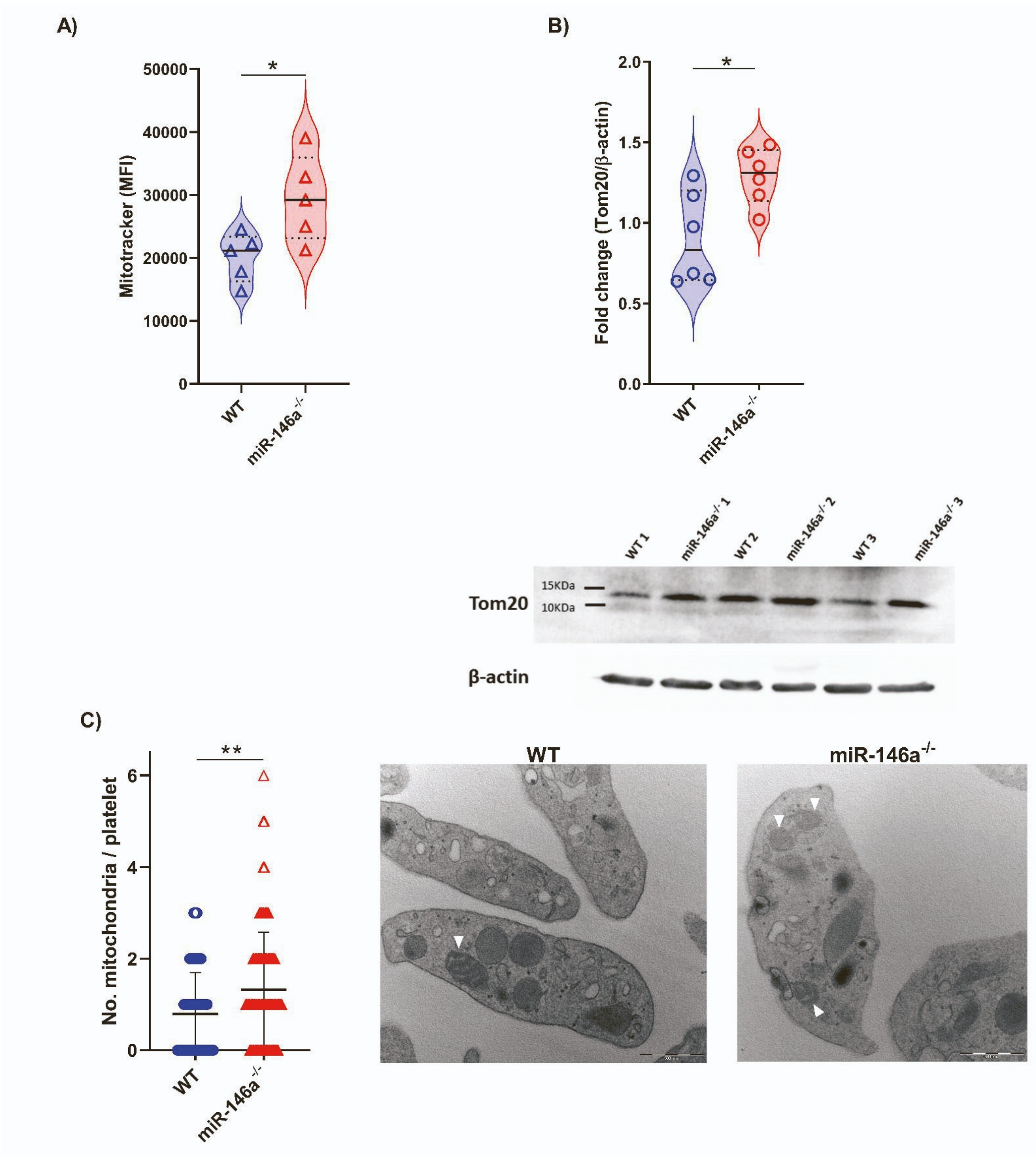

Figure S9. miR-146a deficiency increased mitochondrial mass in platelets. A) Mitotracker green (100nM) determination by flow cytometry, B) Tom20 detection by western blot in WT *vs*. miR-146a$^{-/-}$ resting platelets (a representative blot is shown) and the densitometry Tom20/β-actin was calculated. The statistical analyses were performed by 2-tailed *t*-test (*p<0.05, n=5-6 mice/group). C) Transmission electron microscopy was performed on miR-146a-deficient platelets *vs.* WT. The number of mitochondria per platelet was quantified for 40–50 platelets per mouse in randomly selected images. Scale bar: 500 nm. The Mann-Whitney U test was used for statistical analysis (**p<0.01).

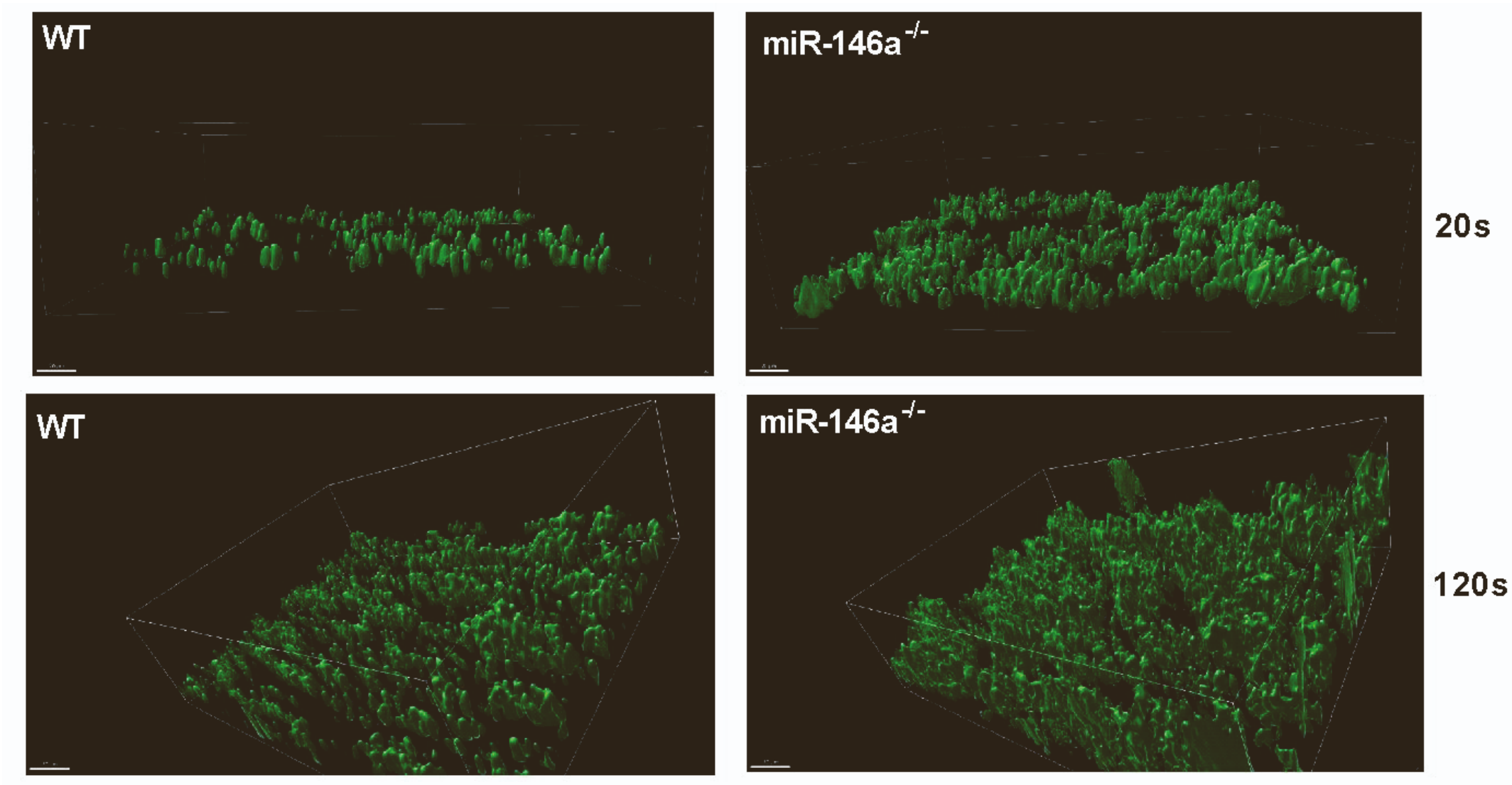

Figure S10. miR-146a$^{-/-}$ platelet hyperreactivity under flow. Whole blood from WT and miR-146$^{-/-}$ mice was perfused over collagen-coated microcapillary at a shear rate of 1100 s^{-1}. Platelets were labeled with $DiOC_6$, and thrombi volumes (μm^3) were calculated using Imaris software. Representative images of 3D aggregates formation at 20s and 120s are shown. Scale bar: 20µm.

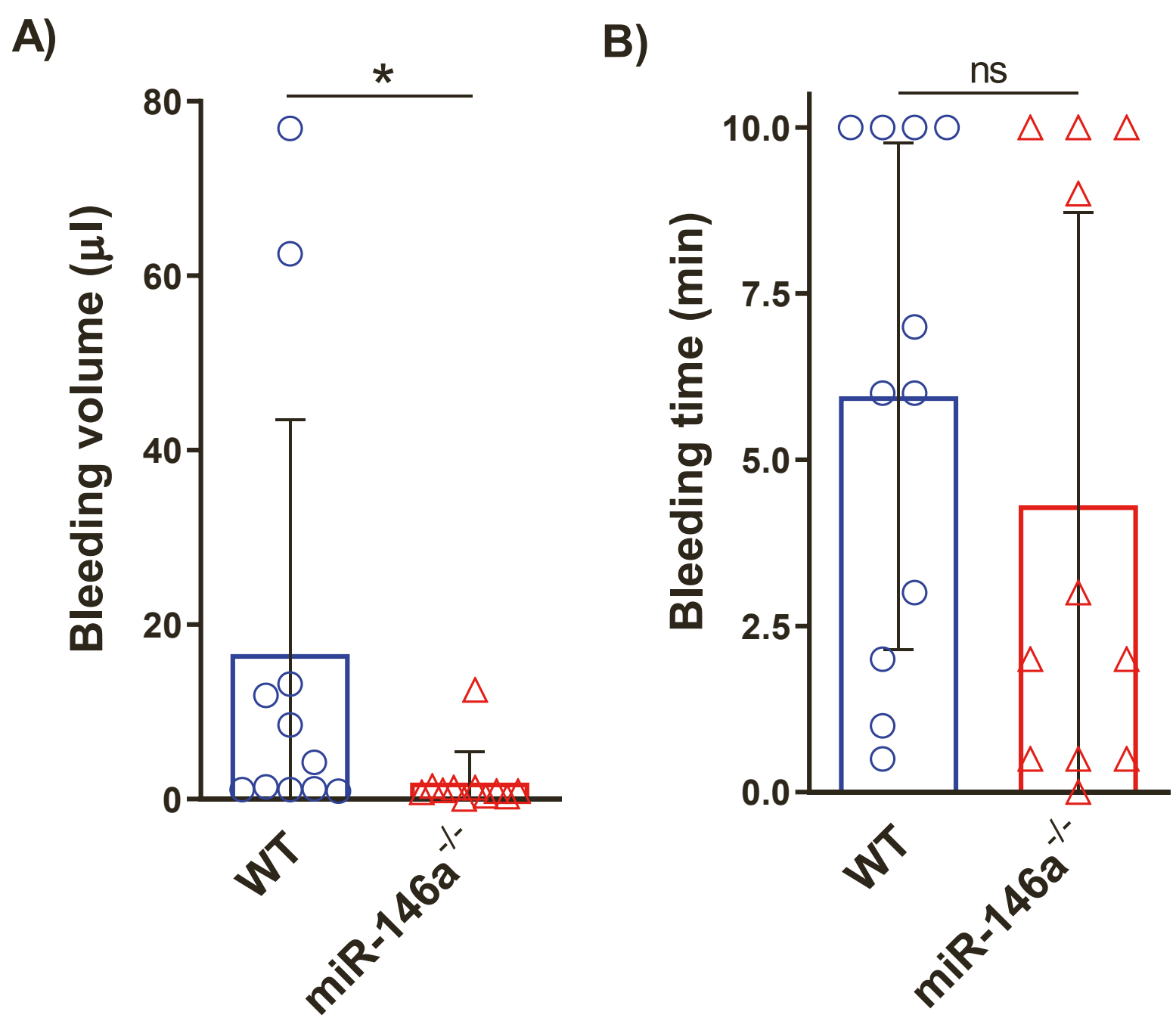

Figure S11. miR-146a deficient mice had reduced bleeding than WT mice. A) Bleeding volume was determined by analyzing the hemoglobin content of blood collected in saline tubes (n=11/group). B) Bleeding was monitored for a maximum of 10 min. The statistical significance was calculated by Mann-Whitney test (*p<0.05, ns=non-significant).

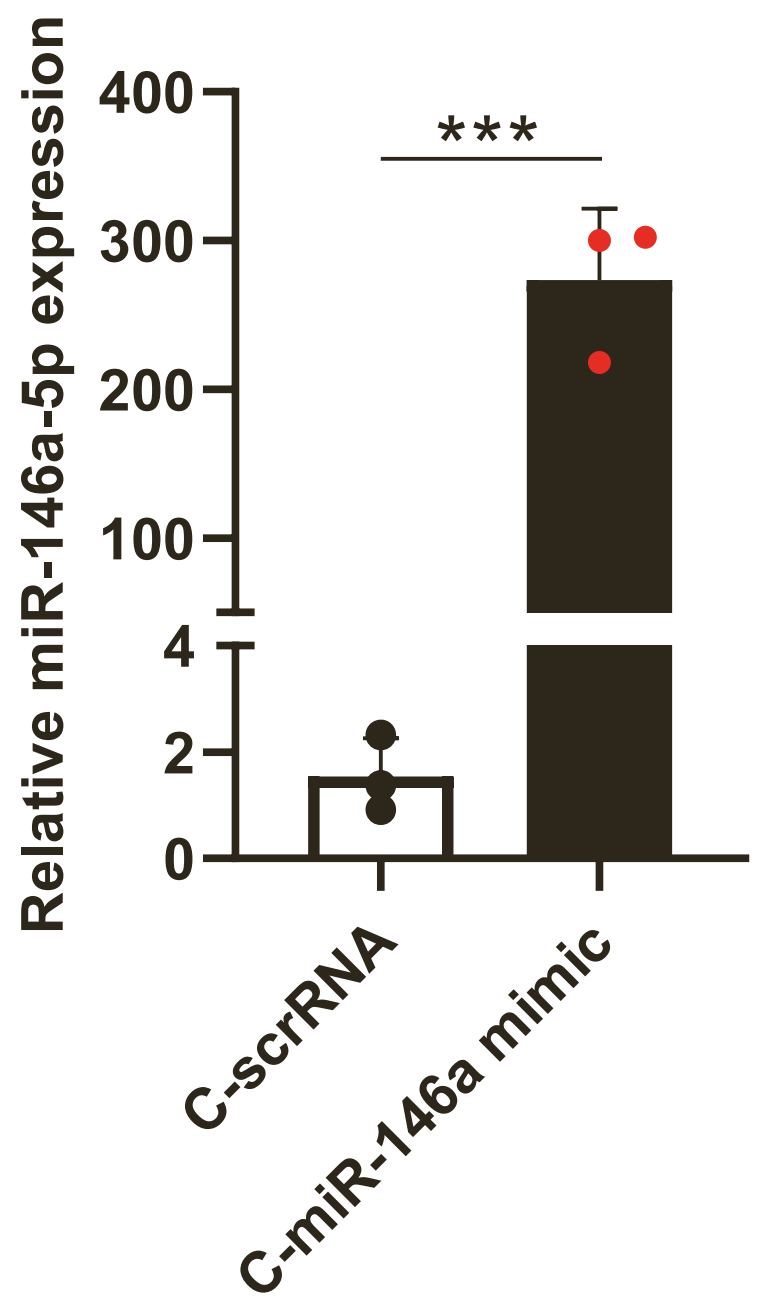

Figure S12. C-miR-146a mimic administration increased 213-fold miR-146a levels in platelets. C-miR-146a mimic and C-scrRNA as a control were intravenously injected for 3 days (10mg/kg). WT mice blood was collected from the cava vein, platelets were washed and then RT-qPCR for miR-146a and U6 was performed. The statistical significance was calculated by 2-tailed *t*-test (***p<0.001).

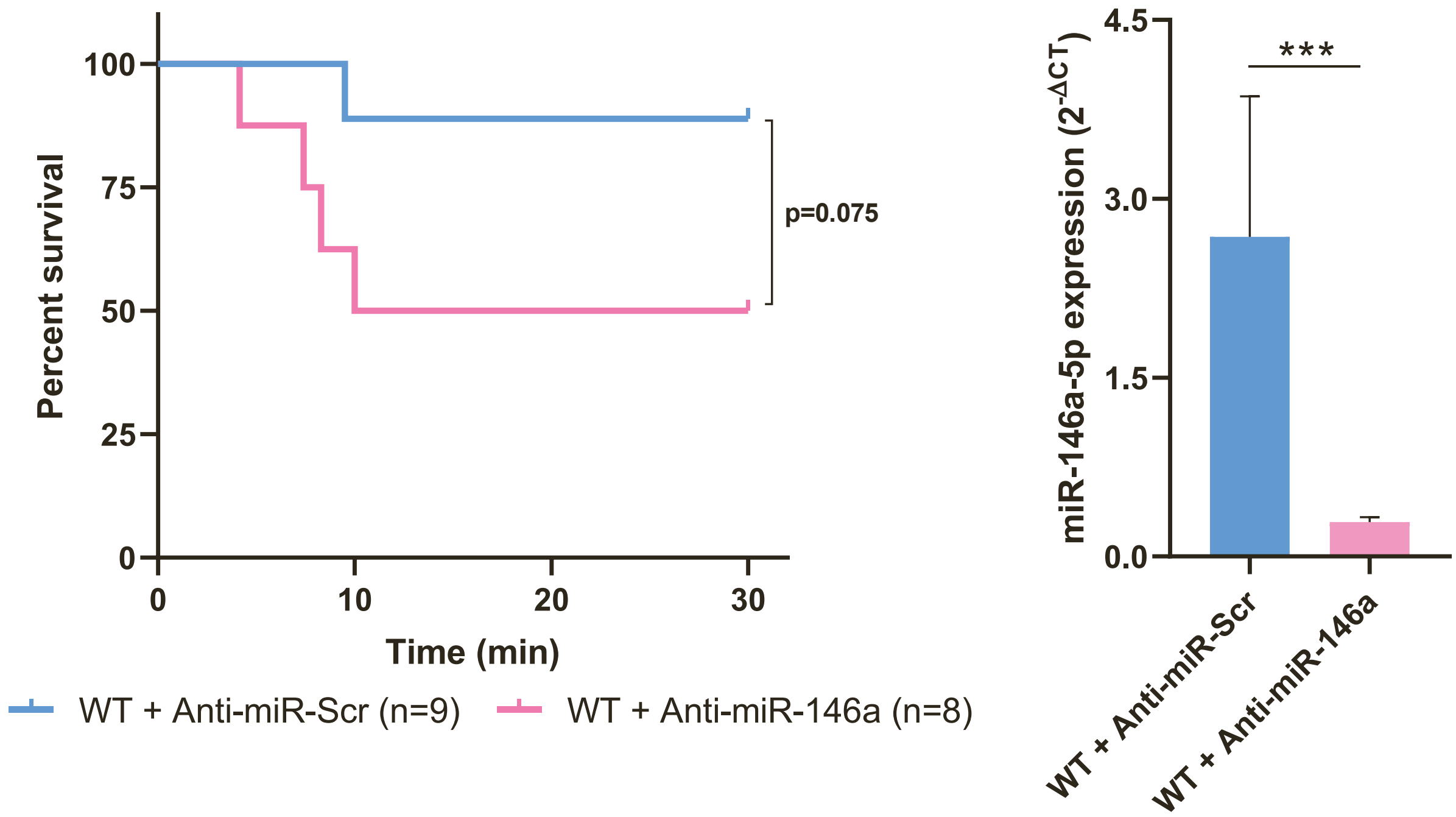

Figure S13. Anti-miR-146a administration decreased miR-146a levels in platelets and impaired the survival of WT mice after systemic thrombosis (PE). AntimiR-146a (n=8) and scrRNA (n=9) as a control were intravenously injected for 2 days (5 mg/Kg). WT mice blood was collected, platelets were washed and then RT-qPCR for miR-146a and U6 was performed. The statistical significance was calculated by Log rank Mantel-Cox test and by 2-tailed *t*-test (***p<0.01), respectively.

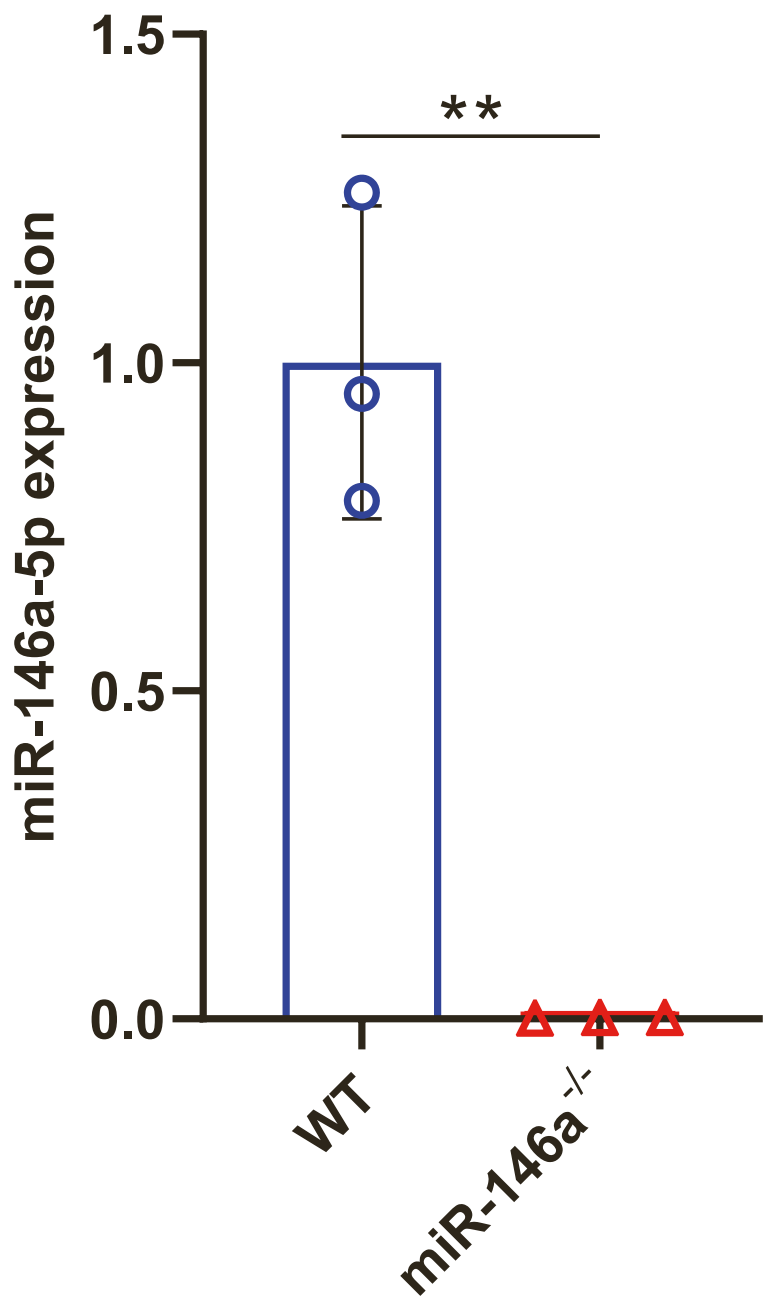

Figure S14. miR-146a levels in platelets from WT and miR-146a deficient mice. administration decreased 9.28-fold miR-146a levels in platelets. WT and miR-146a mice blood was collected, platelets were washed and then RT-qPCR for miR-146a and U6 was performed. The statistical significance was calculated by 2-tailed *t*-test (**p<0.01).

Table S1. Clinical features healthy controls

	rs2431697		
	**CC (n=11)**	**TT (n=10)**	**p-value**
**Age (years)**	41.2 ± 15.1	44.3 ± 12.8	0.6
**Platelets (x10^3/µl)**	237 ± 49	275 ± 79	0.2
**MPV (fl)**	10.9 ± 1.3	10.7 ± 1.1	0.7
**Hb (g/dl)**	14.0 ± 1.2	14.4 ± 2.2	0.6
**Hematocrit (%)**	42.2 ± 3.1	43.2 ± 5.1	0.6
**Neutrophils (x10^3/µl)**	4.0 ± 1.4	3.7 ± 1.2	0.5
**Monocytes (x10^3/µl)**	2.2 ± 0.8	2.0 ± 0.6	0.3
**Lymphocytes (x10^3/µl)**	0.7 ± 0.2	0.6 ± 0.1	0.5

Media ± SD from healthy blood donors homozygous for C or T allele of rs2431697. Their blood samples collected in BAPA or citrate tubes were used to perform T-TAS and platelet activation assays, respectively. P-values were calculated using 2-tailed *t*-test.

Video S1 legend

Microfluidics assay of WT and miR-146a-/- platelets. Mouse blood from WT and miR-146a-/- mice incubated with DiOC6 (2 µM) to label platelets was perfused into the collagen-coated microchannels at 1,100s^{-1}. Platelet adhesion and thrombus formation were visualized and recorded using a fluorescence microscope (Leica Microsystems).
