# Supplementary material for: miR-146a mimic therapy protects against platelet-mediated thrombosis
Source: Mol Ther. 2026 Jul 13;34(9):5333–51. doi: 10.1016/j.ymthe.2026.07.019 (PMC13555580; doi:10.1016/j.ymthe.2026.07.019)
Supplement: Supplementary file 2 [file mmc3.pdf]

Molecular Therapy
Original Article

miR-146a mimic therapy protects against platelet-mediated thrombosis

Sonia Águila,[1,2,3,12] Juan C. García-Cañaveras,[4,12] Laura Zapata-Martínez,[1] Ana B. Arroyo,[1] Nuria García-Barberá,[1] Salvador Carrillo-Tornel,[1] Pedro J. Guijarro-Carrillo,[1] Paula González-Jiménez,[5,6,7] Ana Latorre,[6] María L. Lozano,[1,2,3] Bernard Payrastre,[8,9] José Rivera,[1,2,3] Marie-Pierre Gratacap,[8] Raúl Méndez,[5,6,7,10] Agustín Lahoz,[4] Marcin Kortylewski,[11] Rocío González-Conejero,[1,2,3,13] and Constantino Martínez[1,3,13]

[1]Centro Regional de Hemodonación, Servicio de Hematología Hospital Universitario Morales Meseguer, IMIB Pascual Parrilla, 30003 Murcia, Spain; [2]Universidad de Murcia, 30100 Murcia, Spain; [3]ImuBi, Universidad de Murcia, 30100 Murcia, Spain; [4]Biomarkers and Precision Medicine Unit, Instituto de Investigación Sanitaria Fundación Hospital La Fe, 46026 Valencia, Spain; [5]Pneumology Department, La Fe University and Polytechnic Hospital, 46026 Valencia, Spain; [6]Respiratory Infections, Health Research Institute La Fe (IISLAFE), 46026 Valencia, Spain; [7]Medicine Department, University of Valencia. 46026 Valencia, Spain; [8]INSERM, UMR-S U1297 and University of Toulouse III, Institute of Cardiovascular and Metabolic Diseases (I2MC), CHU-Rangueil, 31432 Toulouse, France; [9]Laboratoire d'Hématologie, CHU de Toulouse, 31059 Toulouse Cedex, France; [10]Centro de Investigación Biomédica en Red de Enfermedades Respiratorias (CIBERES), Instituto de Salud Carlos III, 28029 Madrid, Spain; [11]Center for Gene Therapy and Department of Immuno-Oncology, Beckman Research Institute at City of Hope Comprehensive Cancer Center, Duarte, CA 91010, USA

miR-146a exhibits variable expression levels in humans due to rs2431697, which is associated with cardiovascular events. However, the functional mechanisms linking this genotype with the occurrence of thrombotic events remain unknown. Here, we found that miR-146a partial deficiency was associated with increased platelet activation and a higher incidence of cardiovascular events during hospitalization in patients with community-acquired pneumonia. Accordingly, platelets from *miR-146a*$^{-/-}$ mice were hyperreactive to agonists under static and flow conditions vs. wild type. Metabolomics indicated that *miR-146a*$^{-/-}$ platelets had elevated oxygen consumption rate and acyl-carnitine/carnitine ratio and reduced fatty acid levels. Consistently, *miR-146a*$^{-/-}$ platelets exhibited higher *Cpt1a* expression, a limiting enzyme of fatty acid β-oxidation. Platelet supplementation with palmitate or etomoxir, a Cpt1a inhibitor, increased or normalized *miR-146a*$^{-/-}$ platelets' response, respectively, demonstrating that this deficiency enhanced fatty acid β-oxidation. *In vivo*, *miR-146a*$^{-/-}$ mice demonstrated accelerated lung occlusion leading to a higher mortality rate in a systemic thrombotic model. Injection of a miR-146a mimic targeting myeloid cells in *miR-146a*$^{-/-}$ mice prevented platelet aggregation in lungs by fully protecting them against thrombosis. In summary, our findings show that miR-146a deficiency leads to platelet hyperreactivity through metabolic reprogramming. Elevating miR-146a levels may be a therapy for preventing cardiovascular events.

INTRODUCTION

Cardiovascular diseases (CVDs) stand as the foremost cause of global mortality, with nearly 19 million fatalities reported in 2019, predating the COVID-19 pandemic.[1] This toll is anticipated to rise, given the World Health Organization's projections of increased life expectancy. Platelet hyperreactivity is a pivotal phenomenon fostering the onset of thrombotic events.[2] Traditional pharmacological targets for inhibiting platelet activation encompass $P2Y_{12}$ and cyclooxygenase 1 (COX-1), thereby rendering dual antiplatelet therapy the gold standard prophylaxis for CVDs.[3] However, certain patients exhibit relative resistance to antiplatelet treatment, primarily manifested through high on-treatment platelet reactivity.[4,5] Moreover, the inherent bleeding risk associated with antiplatelet therapy poses a significant concern.[6] Hence, the pursuit of an ideal antiplatelet therapy persists.[3]

Our group and others have identified that patients harboring miR-146a rs2431697-T genotype and other miR-SNPs exhibit a heightened incidence of adverse cardiovascular events (CVEs) across diseases of varied etiologies, including atrial fibrillation, atherosclerosis, or community-acquired pneumonia (CAP), all characterized by a common thromboinflammatory component.[7–10] The preeminent

Received 2 October 2025; accepted 8 July 2026;
https://doi.org/10.1016/j.ymthe.2026.07.019.

[12]These authors contributed equally

[13]Senior author

Correspondence: Sonia Águila, Centro Regional de Hemodonación, Servicio de Hematología Hospital Universitario Morales Meseguer, IMIB Pascual Parrilla, 30003 Murcia, Spain.
E-mail: sonia.aguila@um.es
Correspondence: Rocío González-Conejero, Centro Regional de Hemodonación, Servicio de Hematología Hospital Universitario Morales Meseguer, IMIB Pascual Parrilla, 30003 Murcia, Spain.
E-mail: rociogon@um.es
Correspondence: Constantino Martínez, Centro Regional de Hemodonación, Servicio de Hematología Hospital Universitario Morales Meseguer, IMIB Pascual Parrilla, 30003 Murcia, Spain.
E-mail: constant@um.es

Published by Elsevier Inc. on behalf of The American Society of Gene and Cell Therapy.
This is an open access article under the CC BY license (http://creativecommons.org/licenses/by/4.0/).

Table 1. Clinical and biological features of the CAP patient cohort

	rs2431697			
	All (*n* = 163)	CC (*n* = 34)	CT+TT (*n* = 129)	*p* value
Age, median (IQR)	77 (69–83)	75 (64–83)	78 (70–83)	0.268
Female, *N* (%)	60 (36.8)	9 (26.5)	51 (39.5)	0.160
Diabetes mellitus, *N* (%)	60 (36.8)	16 (47.1)	44 (34.1)	0.164
Heart disease, *N* (%)	56 (34.4)	7 (20.6)	49 (38.0)	0.057
History coronary artery disease, *N* (%)	26 (16.0)	4 (11.8)	22 (17.1)	0.454
Hypertension, *N* (%)	97 (60.2)	22 (66.7)	75 (58.6)	0.398
Dyslipidemia, *N* (%)	80 (49.1)	14 (41.2)	66 (51.2)	0.300
Current smoking, *N* (%)	36 (22.1)	8 (23.5)	28 (21.7)	0.820
CURB65, median (IQR)	2 (1–3)	2 (1–2)	2 (1–3)	0.229
ASA, *N* (%)	40 (24.5)	9 (26.5)	31 (24.0)	0.353
Platelet $\times$ 10^9/L, median (IQR)	235 (177–304)	261 (181–367)	227 (174–280)	0.072
Mean platelet volume, median (IQR)	10.95 (10.3–11.7)	10.75 (9.9–11.1)	11.0 (10.3–11.9)	0.060
CVE during hospitalization,[a] *N* (%)	16 (9.8)	0 (0)	16 (12.4)	0.031[b]

CURB65, score for pneumonia severity (ranges from 0 to 5); ASA, aspirin; CVE, cardiovascular event.
[a]Acute coronary syndrome (*n* = 2), arrythmias (*n* = 11), heart failure (*n* = 4), and cerebrovascular accident (*n* = 1).
[b]Significant value: $p < 0.05$.

function ascribed to miR-146a is its regulatory role in immune response.[11,12] Numerous studies have implicated various microRNAs (miRNAs), such as miR-148a,[13] miR-223,[14] or miR-126,[15] as key players in platelet function and thrombosis. Despite the evident association between miR-146a levels and CVD,[16] its involvement in platelet function remains unexplored.

By employing miR-146a-deficient mice, the present work demonstrates a key role for miR-146a as a regulator of platelet activation via metabolic reprogramming, through enhanced fatty acid (FA) β-oxidation (FAO), fostering a prothrombotic phenotype. Importantly, miR-146a overexpression via miRNA mimic therapy confers complete protection against systemic collagen/epinephrine-induced thrombosis and the subsequent mortality (100% survival). Translation to humans showed that rs2431697-T allele carriers, who have decreased miR-146a levels,[17] exhibit platelet hyperreactivity, both in healthy subjects under *ex vivo* stimulation and in patients with CAP upon pathological stress.

Thus, our results underscore miR-146a as a therapy against thrombosis and CVD by targeting metabolism, thereby diverging from existing antiplatelet drugs.

RESULTS

The rs2431697-T allele induced greater baseline platelet reactivity over time and increased the risk of cardiovascular complications in patients with CAP

In humans, the rs2431697-T allele causes partial miR-146a deficiency,[17] as we confirmed in platelets from healthy donors (Figure S1), and it has been linked to CVEs in various thromboinflammatory diseases.[7,9,10] To further investigate this association, a cohort of patients with CAP was recruited and genotyped (*n* = 346). We found that the frequencies of the miR-SNP rs2431697 (T allele = 0.56 in Europeans) deviated from Hardy-Weinberg equilibrium (18.2% CC, 36.7% CT, and 45% TT, $p < 0.0001$) in the CAP cohort, indicating a lower rate of hospitalization among CC individuals compared to T-allele carriers.

Then, platelet activation state was assessed at different time points after hospitalization, i.e., days 1, 4–5, and 30 (Table 1; *n* = 163, consisting of 34 CC and 129 T-allele carriers). The results showed that basal P-selectin exposure was elevated in platelets from CT+TT patients over time compared to CC individuals (Figures 1A–1C), with statistically significant differences observed on days 1 and 4–5 (p = 0.033 and p = 0.013, respectively; Figures 1A and 1B). Importantly, T-allele carriers with CAP experienced a higher incidence of cardiovascular outcomes during hospitalization (12.4% CT+TT vs. 0% CC, p = 0.030) (Table 1).

Overall, partial miR-146a deficiency exacerbates platelet activation in thromboinflammatory diseases such as CAP, contributing to an increased incidence of CVE in affected patients. Thus, our findings demonstrate a significant and previously unreported association between a miR-SNP and platelet reactivity.

In healthy subjects, the presence of the rs2431697-TT genotype promotes platelet hyperactivation upon stimulation

After finding an association between platelet reactivity and rs2431697 in patients with CAP, we explored the impact of this miR-SNP on platelet function in healthy individuals. Peripheral whole blood was collected from individuals homozygous for either the C or T allele of the miR-SNP (see Table S1).

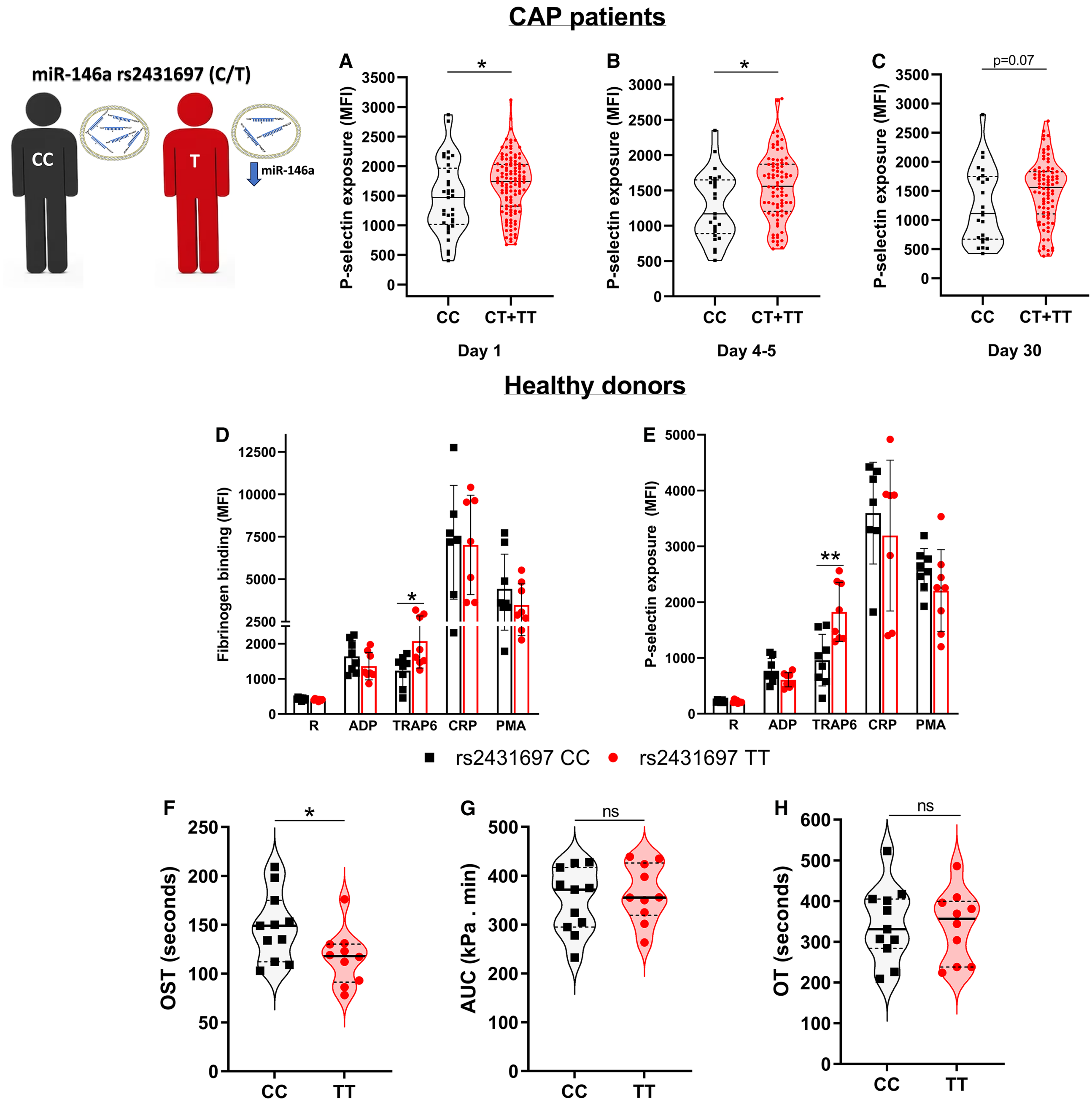

Figure 1. rs2431697-T allele promoted platelet activation in humans

The scheme shows decreased miR-146a levels by ∼50% in cells from rs2431697-T allele carriers. Basal platelet activation from the cohort of patients with community-acquired pneumonia (CAP) (n = 163) was measured by flow cytometry on days (A) 1 (33 CC and 117 CT+TT), (B) 4–5 (25 CC and 82 CT+TT), and (C) 30 (24 CC and 85 CT+TT) after hospital admission. Median fluorescence intensity (MFI) of P-selectin or CD62P was represented. A two-tailed t test was used to calculate statistical significance (*$p < 0.05$). (D and E) Platelets from healthy blood donors were activated using different agonists (10 μM ADP, 25 μM TRAP-6, 10 μg/mL CRP, and 100 nM PMA), in which (D) fibrinogen binding and (E) P-selectin exposure were determined by flow cytometry (n = 8/group). The total thrombus formation analysis system (T-TAS) was used to study platelet aggregation under shear conditions (n = 10–11/group). Whole blood was perfused over a collagen chip, registering pressure changes. (F) Occlusion start time (OST), (G) area under the curve (AUC), and (H) occlusion time (OT) were measured. The statistical analysis for these assays was performed by a two-tailed t test (*$p < 0.05$ and **$p < 0.01$). All samples were genotyped and classified by rs2431697.

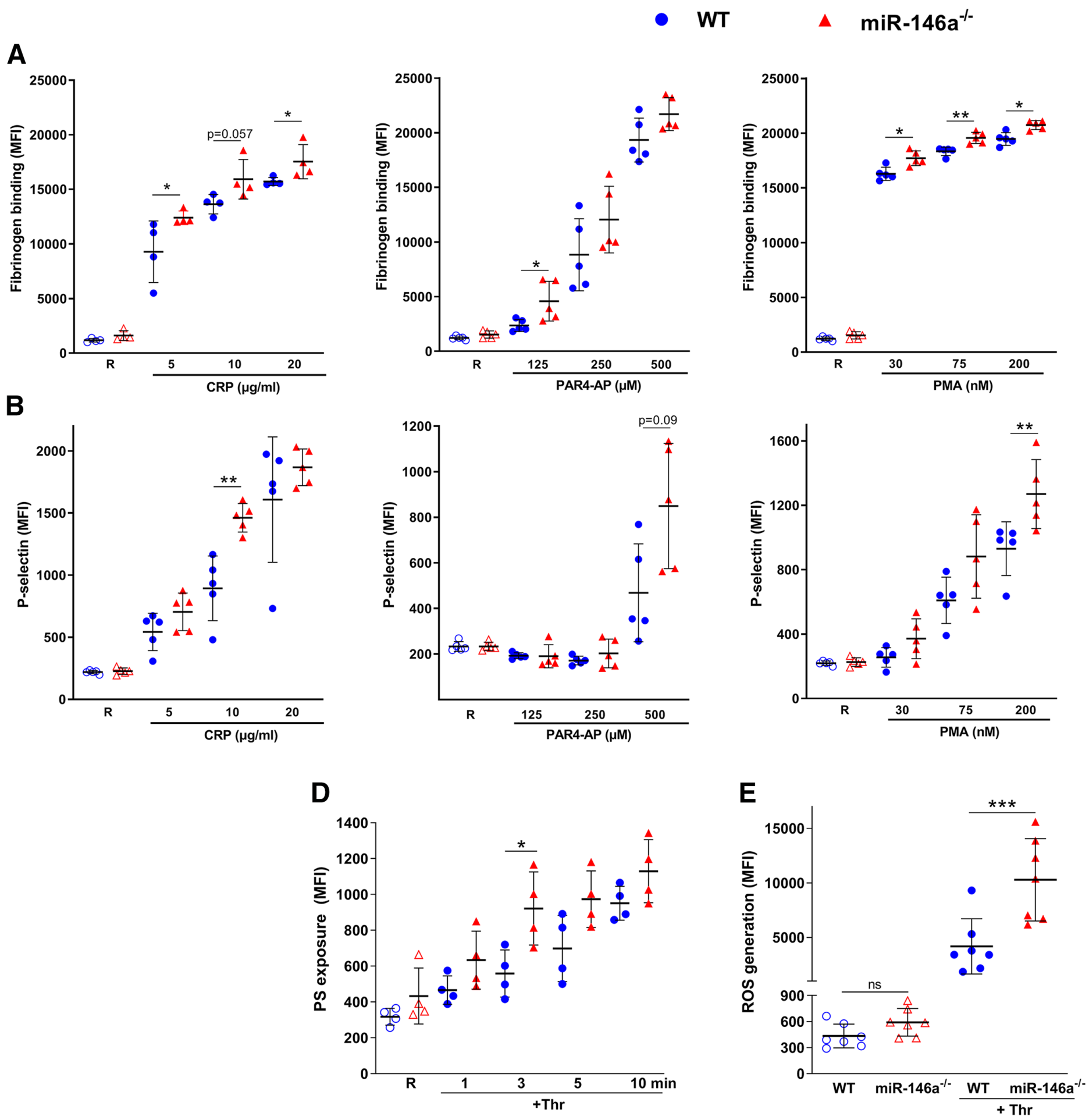

Figure 2. miR-146a deficiency caused platelet hyperactivation

Platelet activation was assessed in diluted blood from WT and *miR-146a*$^{-/-}$ mice with different agonists (collagen-related peptide [CRP], PAR4-agonist peptide [PAR4-AP], phorbol-12-myristate-13-acetate [PMA], and adenosine diphosphate [ADP]) and concentrations. (A) Fibrinogen binding and (B) P-selectin exposure were determined by flow cytometry. (C) The main receptor's expression was measured by flow cytometry. (D) Platelet phosphatidylserine (PS) exposure over time following thrombin (Thr) activation and (E) ROS (H_2DCFDA) generation at basal and stimulated state (Thr). For all assays, n = 4–9 mice/group were used (mean ± SD). Statistical analyses were performed using a two-tailed t test, a Mann-Whitney U test, or a one-way ANOVA (*$p < 0.05$, **$p < 0.01$, ***$p < 0.001$, and ****$p < 0.0001$).

The study revealed that individuals with the TT genotype exhibited significantly greater fibrinogen-binding ability upon TRAP-6 activation than those with the CC genotype (p = 0.018, Figure 1D). This heightened platelet reactivity was further evidenced by increased P-selectin exposure in response to the same agonist (p = 0.004, Figure 1E). Additionally, platelet reactivity was assessed under flow conditions using the total thrombus formation analysis system (T-TAS). Notably, individuals with the TT genotype displayed a shorter occlusion start time (OST) compared to those with the CC genotype (p = 0.035, Figure 1F), indicating accelerated initial aggregate formation. However, no significant differences were observed in occlusion time (OT) or the area under the curve (AUC) between the two groups (Figures 1G and 1H).

Our results indicate that reduced miR-146a expression in healthy individuals enhances platelet activation and expedites the formation of initial aggregates only under stimulation, suggesting a potential prothrombotic phenotype that might worsen under stress or pathological conditions.

miR-146a deficiency enhances platelet activation

To elucidate the underlying mechanisms of this observation in human platelets, *miR-146a*$^{-/-}$ mice were used. *miR-146a*$^{-/-}$ mice exhibited no alterations in platelet count or mean platelet volume compared to wild-type (WT) littermates (Figure S2). We then explored the impact of miR-146a deficiency on platelet activation. Platelets from both WT and *miR-146a*$^{-/-}$ mice were subjected to stimulation with increasing concentrations of various agonists (PAR4-AP, CRP, PMA, and ADP). *miR-146a*$^{-/-}$ platelets showed heightened fibrinogen binding (Figure 2A) and P-selectin exposure (Figure 2B) across all tested agonists. Then, we investigated whether this enhanced response was associated with differences in the

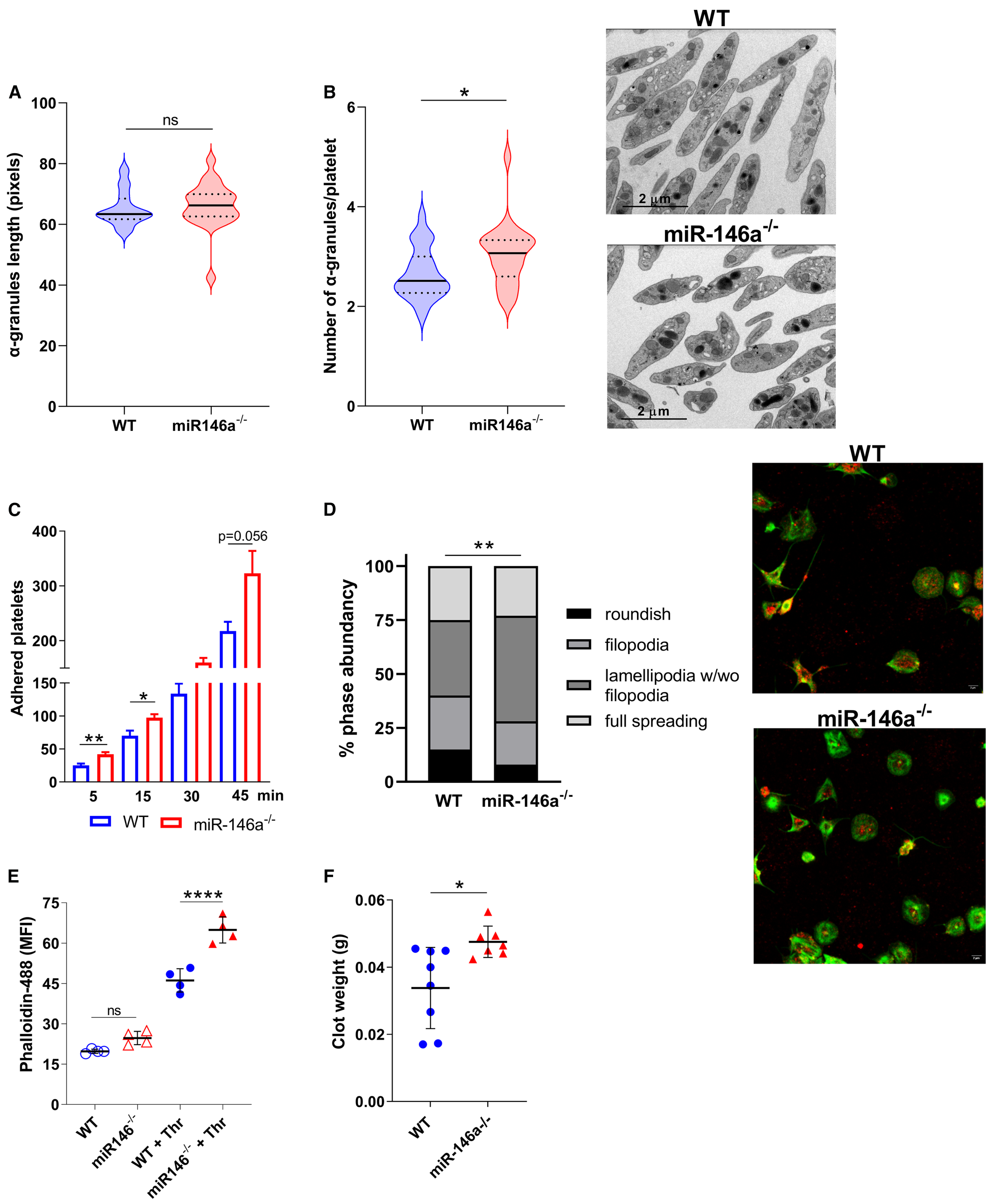

(legend on next page)

expression of platelet receptors. However, no differences were observed in the expression of the main platelet receptors (Figure 2C). Interestingly, $P2ry_1$ expression was significantly elevated in *miR-146a*$^{-/-}$ platelets compared to WT (Figure 2C, p = 0.001). $P2ry_1$, an essential G protein-coupled ADP receptor, initiates platelet activation induced by ADP, potentially explaining the increased fibrinogen binding observed in deficient platelets following ADP activation (Figure 2A, p = 0.039). Indeed, *in silico* prediction suggested that *P2ry1* mRNA could be a potential direct target for miR-146a (Figure S3A), which we further demonstrated *in vitro* using a luciferase assay in HEK293 T cells (Figure S3B). Furthermore, miR-146a-deficient platelets exhibited higher levels of phosphatidylserine (PS) exposure on the platelet surface as well as increased reactive oxygen species (ROS) generation compared to WT following thrombin (Thr) stimulation (p = 0.042 and p = 0.0003, respectively) (Figures 2D and 2E). Thus, miR-146a deficiency promotes a shift from the typical surveillance platelet phenotype toward a procoagulant phenotype upon activation by Thr.

Collectively, these findings underline that *miR-146a*$^{-/-}$ platelets display increased reactivity across various stimuli, highlighting the role of miR-146a in modulating platelet function.

Accelerated spreading and higher α-granule content in miR-146a-deficient platelets

Next, we evaluated platelet ultrastructure using transmission electron microscopy. While there were no observable differences in α-granule size (Figure 3A), miR-146a-deficient platelets exhibited a significantly higher number of α-granules compared to WT (p = 0.031, Figure 3B). Afterward, we investigated platelet cytoskeleton dynamics by assessing their spreading ability on a fibrinogen matrix and their F-actin levels following Thr activation. A greater number of miR-146a-deficient platelets adhered to and spread onto the fibrinogen matrix (Figure 3C), with increased lamellipodia phenotype compared to WT platelets (Figure 3D). Correspondingly, miR-146a-deficient platelets displayed elevated F-actin levels upon activation (Figure 3E, p < 0.0001). Furthermore, *miR-146a*$^{-/-}$ platelets formed larger clots than WT platelets (Figure 3F, p = 0.014). However, there was no significant acceleration in clot retraction (Figure S4). Therefore, miR-146a deficiency disturbs functional cytoskeletal dynamics, leading to enhanced platelet spreading and clot formation.

miR-146a$^{-/-}$ platelets exhibit enhanced mitochondrial respiration

Given the pleiotropic impact of miR-146a deficiency on platelet function observed in previous results, we explored the possibility of an alteration in platelet bioenergetics as a shared pathway. Platelets demonstrate considerable metabolic adaptability supported by oxidative phosphorylation (OXPHOS), glycolysis, and FAO. To investigate whether miR-146a-deficient platelets display a distinct bioenergetic profile, we utilized Seahorse extracellular flux analysis to monitor the oxygen consumption rate (OCR) and extracellular acidification rate (ECAR) in response to stimuli and/or mitochondrial modulators. Regarding OCR, *miR-146a*$^{-/-}$ platelets exhibited augmented maximal respiration capacity (MRC) and spare capacity (SC) compared to WT platelets in resting conditions (p < 0.0001 for both; Figure 4A), indicating that the mitochondria in miR-146a-deficient platelets had a higher respiration rate when faced with metabolic challenges (Figure 4A).

Remarkably, these differences in OCR were magnified after stimulation with various agonists (Figures 4B–4E). MRC was higher in miR-146a-deficient platelets compared to WT platelets under activated conditions. Whereas WT platelets exhibited a depleted SC, *miR-146a*$^{-/-}$ platelets retained a functional SC following Thr and PMA activation (p < 0.0001 for both, Figures 4B and 4D). Regarding other agonists, *miR-146a*$^{-/-}$ platelets displayed less severe SC depletion vs. WT platelets after activation (Figures 4C and 4E).

Nonetheless, no differences were observed in non-mitochondrial respiration between WT and miR-146a-deficient platelets (Figures 4A–4E). These findings point out that in *miR-146a*$^{-/-}$ platelets, mitochondria play a crucial role in enhancing their ability to respond to various challenges, potentially explaining the hyperreactive phenotype of these platelets. Additionally, *miR-146a*$^{-/-}$ platelets exhibited significantly elevated ECAR compared to WT platelets under both conditions, indicating more efficient compensatory flux through glycolysis in *miR-146a*$^{-/-}$ platelets (Figure S5).

Thus, miR-146a-deficient platelets demonstrate a higher energetic profile at rest and under stimulated conditions, characterized by elevated OCR, MRC, and SC.

Enhanced FA oxidation and *Cpt1a* expression in miR-146a-deficient platelets

To elucidate the specific metabolic pathways underlying the differences in the bioenergetic profiles of WT and *miR-146a*$^{-/-}$ platelets, we carried out an untargeted metabolomics analysis in resting and activated platelets (Thr, PAR-4-AP, and CRP). Initially, we performed a non-supervised analysis of the data. The scores plot of the principal-component analysis (PCA) clearly depicted a separation of the data into four groups (i.e., WT control, WT treated,

Figure 3. α-Granules and cytoskeleton alterations in miR-146a-deficient platelets

Washed platelets from WT and miR-146a-deficient mice were characterized by transmission electron microscopy (TEM). (A) α-Granule length and (B) α-granule count were quantified in ≥100 platelets; scale bar: 2 μm. Spreading assays: F-actin (green) and β-tubulin (red) were labeled, (C) number of adhered platelets at different time points and (D) phenotype of spread platelets at 45 min were quantified in 2–3 images (1,870 × 1,860), and statistical significance was determined by multiple unpaired *t* tests; scale bar 2 μm. (E) Platelet F-actin content after thrombin (Thr) activation for 2 min. (F) Clot retraction assay; clot weight was measured after 150 min. The results are represented as mean ± SD (n = 3–8 mice/group in all experiments), and the significances were calculated by a two-tailed *t* test, a Mann-Whitney U test, or a one-way ANOVA (*p < 0.05, **p < 0.01, ***p < 0.001, and ****p < 0.0001) depending on the assay.

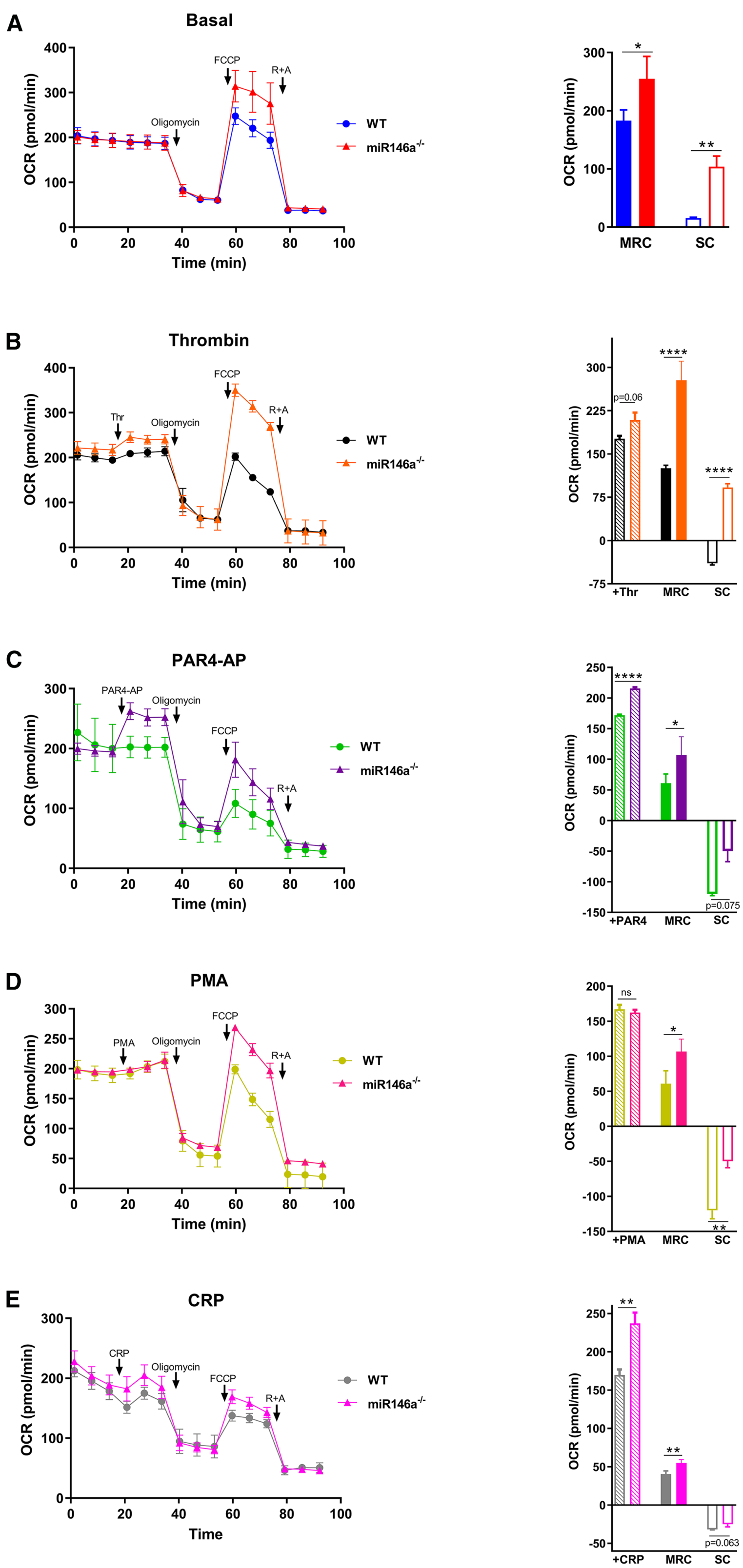

Figure 4. *miR-146a*$^{-/-}$ platelets presented greater mitochondrial respiration

Oxygen consumption rate (OCR) was determined by Seahorse Analyzer using the Cell Mito Stress Test kit. OCR, maximal respiratory capacity (MRC), and spare capacity (SC) in platelets from WT vs. *miR-146a*$^{-/-}$ were calculated at the (A) basal and (B–E) activated states with (B) Thr (0.5 IU/mL), (C) PAR-4AP (500 μM), (D) PMA (200 nM), and (E) CRP (20 μg/mL) (pool of 6 mice/group, mean ± SD). The significance was calculated by a two-tailed *t* test or a Mann-Whitney U test (**p* < 0.05, ***p* < 0.01, ****p* < 0.001, and *****p* < 0.0001).

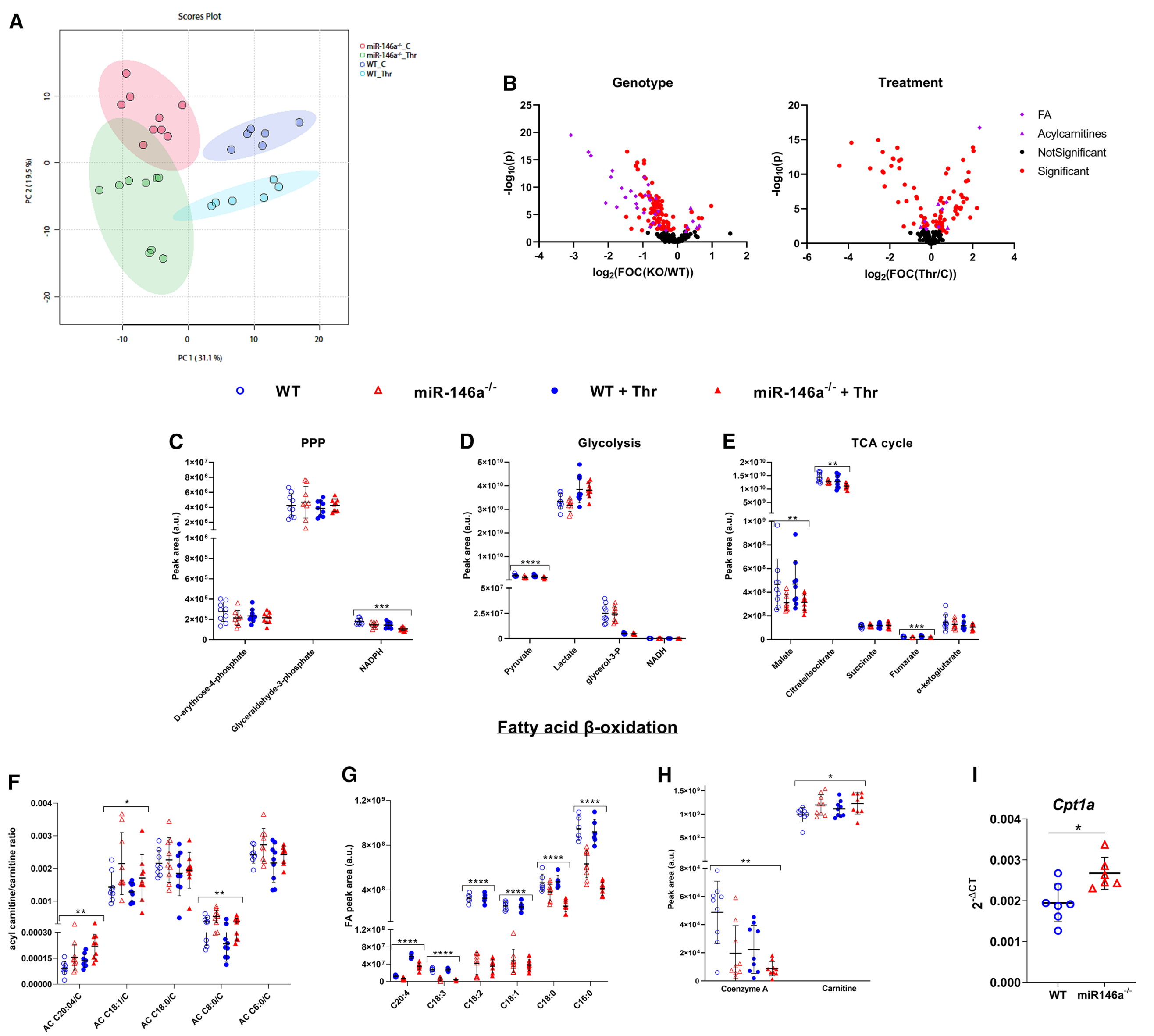

Figure 5. Metabolomics showed enhanced fatty acid β-oxidation for miR-146a-deficient platelets

Untargeted metabolomics was performed in WT vs. *miR-146a*$^{-/-}$ platelets ± agonist 0.5 IU/mL Thr by Q Exactive Plus Hybrid Quadrupole-Orbitrap mass spectrometer (2–3 pools of 3 mice/group and technical triplicates). (A) Principal-component analysis (PCA) and (B) volcano plot of platelet metabolome. Main intermediates or products of (C) pentose phosphate pathway (PPP), (D) glycolysis, and (E) tricarboxylic acid cycle (TCA). (F) Acylcarnitine/carnitine (AC/C) ratio, (G) fatty acid levels, and (H) coenzyme A and carnitine levels in resting or stimulated (Thr) platelets. (I) Expression of *Cpt1a* mRNA measured by qPCR ($n = 6$–7 pools of 3–4 mice/group), and the significance was determined by a two-tailed *t* test (*$p < 0.05$). For the remaining assays, statistical significance was calculated by a two-way ANOVA (*$p < 0.05$, **$p < 0.01$, ***$p < 0.001$, and ****$p < 0.0001$); the genotype statistical significance is represented in graphs.

miR-146a$^{-/-}$ control, and *miR-146a*$^{-/-}$ treated) in every tested stimulus. Hence, the first principal component primarily accounted for the variance due to genotype (∼30% explained variance), while the second component accounted for the variance due to agonist treatment (∼20% explained variance) (Figures 5A, S6A, and S7A). To identify the metabolic signature driving these changes, we used a two-way ANOVA and identified metabolites with differential abundances attributable to genotype or treatment. In particular, FAs and acyl-carnitines, key metabolites involved in FAO, exhibited differential abundances not only between WT and *miR-146a*$^{-/-}$ resting platelets but also after stimulation (Figures 5B, S6B, and S7B).

Analysis of the most relevant pathways in platelets revealed alterations to the pentose-phosphate pathway (PPP), glycolysis, and

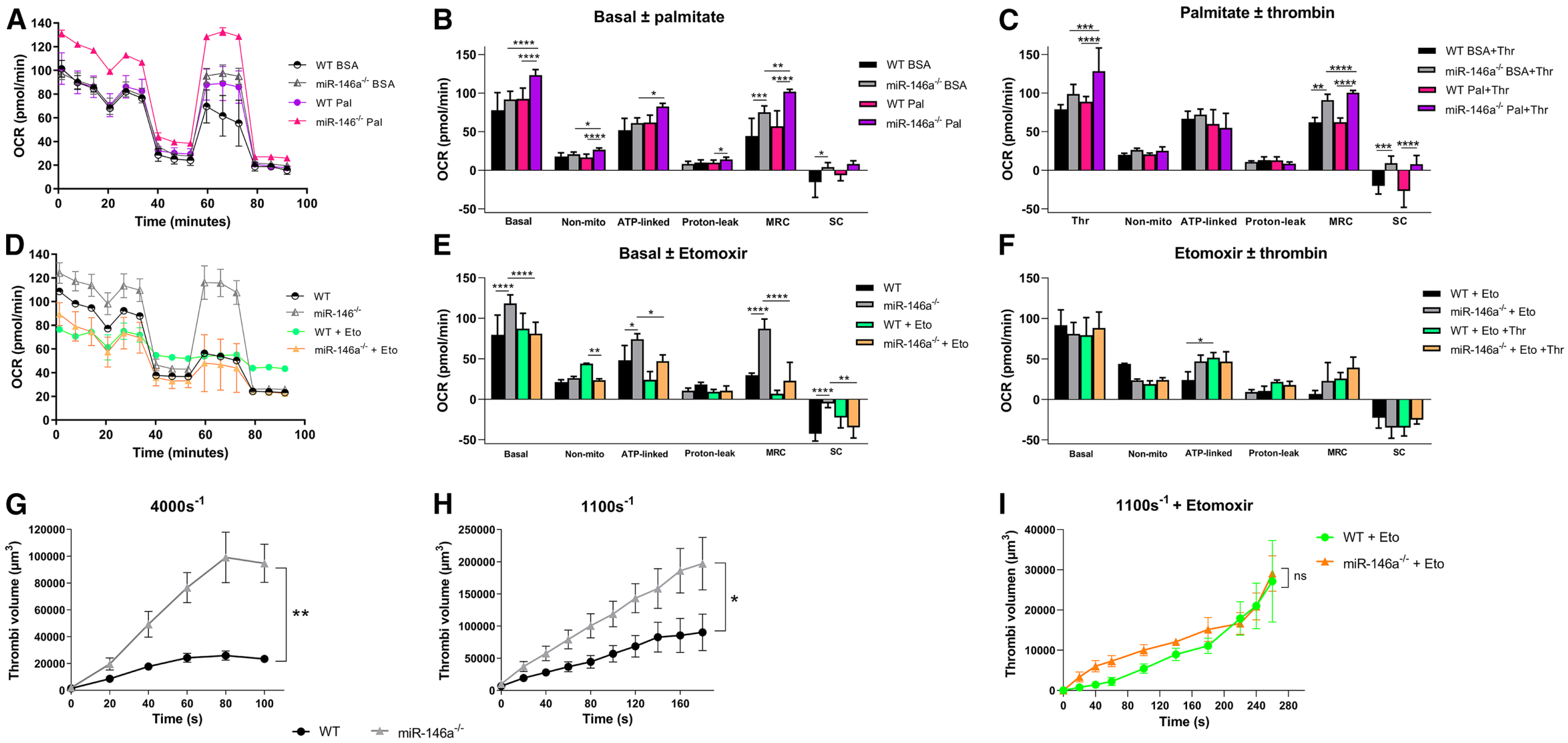

Figure 6. Fatty acid metabolism supported higher respiration in *miR-146a*$^{-/-}$ platelets

Oxygen consumption rate (OCR) was determined by Seahorse Analyzer using the Cell Mito Stress Test kit. WT or *miR-146a*$^{-/-}$ platelets were incubated with palmitate conjugated with BSA (Pal, 150 μM), a long-chain fatty acid, BSA as a control, or etomoxir (Eto; 5 μM), a specific Cpt1a inhibitor. (A) OCR profile in the presence or absence of palmitate for resting platelets. (B) Basal respiration, non-mitochondrial respiration, ATP production, proton-leak, maximal respiratory capacity (MRC), and spare capacity (SC) were calculated. (D) OCR profile in the presence or absence of Eto of WT vs. *miR-146a*$^{-/-}$ resting platelets. (E) Basal respiration, non-mitochondrial respiration, ATP production, proton leak, MRC, and SC were calculated. For stimulated (0.5 IU/mL Thr) platelets, basal respiration, non-mitochondrial respiration, ATP production, proton leak, MRC, and SC were determined in the presence or absence of palmitate (C) or following Eto (F) incubation without Thr (n = 2–3 mice/group and condition; values are mean ± SD). The significance was calculated by one-way ANOVA (*$p < 0.05$, **$p < 0.01$, ***$p < 0.001$, and ****$p < 0.0001$). Whole blood was perfused over a collagen-coated microcapillary at a shear rate of 4,000 (G) or 1,100 (H) s^{-1}. (I) Whole blood was perfused over a collagen-coated microchannel at 1,100 s^{-1} after 30 min of Eto incubation (4 μM). Platelets were labeled with $DiOC_6$, and thrombus volumes (μm^3) were calculated using Imaris software; values are mean ± SEM for independent experiments. For the statistical analysis, the area under the curve of each mouse was determined and analyzed by a two-tailed Mann-Whitney U test or *t* test (n = 4–10 mice/group; *$p < 0.05$, **$p < 0.01$, and ns, non-significant).

tricarboxylic acid cycle (TCA) between genotypes, albeit not in relation to Thr (Figures 5C–5E), PAR4-AP (Figures S6C–S6E), or CRP (Figures S7C–S7E) activation. Interestingly, several free FA levels were decreased, while total carnitines (free carnitine and acyl-carnitines) and acyl-carnitine/carnitine (AC/C) ratio were increased in *miR-146a*$^{-/-}$ platelets compared to WT, including some of the most abundant species. FA levels further decreased upon activation of miR-146a-deficient platelets (except for arachidonic acid [C20:4]), suggesting their utilization as an energy source (Figures 5F, 5G, S6F, S6G, S7F, and S7G). Moreover, free coenzyme A levels, a key cofactor in FA catabolism, were lower in *miR-146a*$^{-/-}$ platelets and further decreased under stimulation (Figures 5H, S6H, and S7H). In contrast, the serum metabolite profiles of *miR-146a*$^{-/-}$ and WT mice did not show substantial differences (R^2 = 0.993; Figure S8), supporting that changes in FA levels and AC/C ratio were intrinsic to platelets and not due to systemic differences in nutrient availability.

Taken together, these results show that miR-146a deficiency induces metabolic reprogramming in platelets characterized by enhanced FAO. A critical rate-limiting enzyme for FAO is CPT1A, located in the outer mitochondrial membrane, which mediates the conjugation of FA to carnitine for their import into the mitochondria. Consistently, *miR-146a*$^{-/-}$ platelets exhibited higher *Cpt1a* expression compared to WT platelets (Figure 5I, p = 0.011).

We hypothesized that the observed higher *Cpt1a* expression and AC/C ratio in miR-146a-deficient platelets could be related to an increased mitochondrial mass. Accordingly, we measured mitochondrial content using two different methods: MitoTracker labeling (Figure S9A) and Tom20 detection by western blot (Figure S9B). Both assays revealed a significant increase in mitochondrial mass in *miR-146a*$^{-/-}$ platelets compared to WT platelets (p = 0.029 and p = 0.017, respectively; Figure S9), which was confirmed by detecting a greater number of mitochondria per platelet in miR-146a-deficient platelets compared to WT (p = 0.0022, Figure S9C).

Palmitate or Cpt1a inhibitor supplementation delineates the OCR profile of *miR146a*$^{-/-}$ platelets

Building upon the metabolomics profile of miR-146a-deficient platelets and their heightened expression of *Cpt1a*, we analyzed whether these platelets metabolized FAs more efficiently than WT platelets.

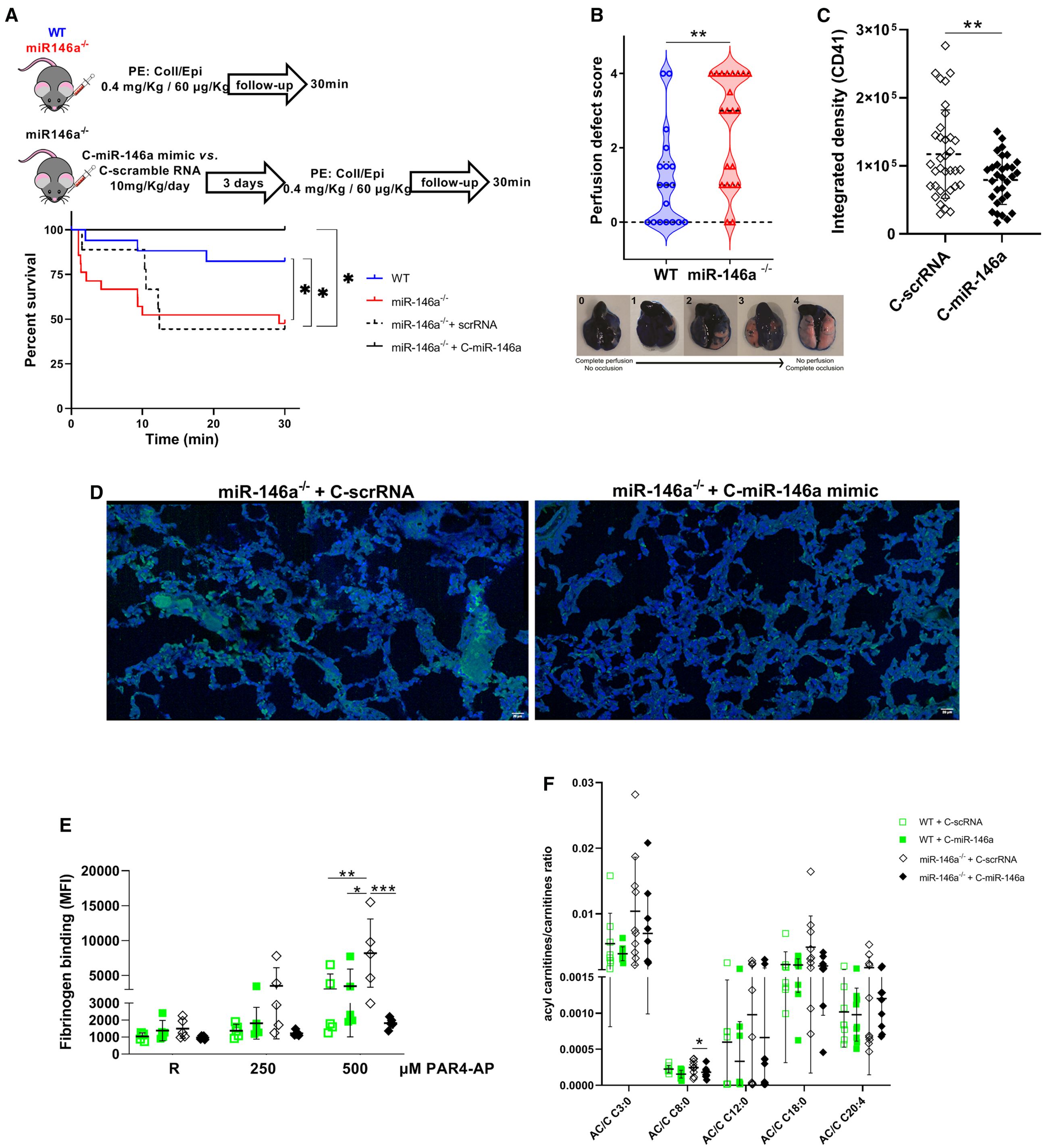

Figure 7. miR-146a mimic therapy led to survival after pulmonary embolism in *miR-146a*$^{-/-}$ mice

Pulmonary embolism (PE) was induced by injecting collagen and epinephrine in WT and *miR-146a*$^{-/-}$ mice (n = 17–21 mice/group). (A) Percentage of survival of these mice and *miR-146a*$^{-/-}$ mice following miR-146a mimic administration (n = 9 mice/group) for 3 days before PE induction was monitored 30 min after PE challenge. Log-rank Mantel-Cox test was applied to determine the statistical significance. (B) Perfusion defect score of lungs was calculated by Evans Blue injection in the heart of WT and *miR-146a*$^{-/-}$ mice following the respiratory arrest. A Mann-Whitney U test was used for statistical analysis. (C) Quantification of integrated density of platelet aggregates (CD41) in lungs from miR-146a-deficient mice treated with miRNA therapy (C-scrRNA or C-miR-146a mimic) after PE induction. At least 3 lung sections were analyzed per mouse

(legend continued on next page)

For this purpose, we performed new extracellular flux analyses using bovine serum albumin (BSA)-conjugated palmitate as a substrate. Palmitate exerted an influence on the OCR profile for both genotypes. However, OCR was significantly higher in *miR-146a*$^{-/-}$ platelets compared to WT following FA addition ($p < 0.0001$; Figures 6A and 6B), thereby amplifying the differences in MRC compared with basal conditions ($p < 0.0001$, Figures 6A and 6B). Interestingly, the presence of the Cpt1a inhibitor etomoxir[18] led to a reduction in OCR in both genotypes, and the differences in mitochondrial respiration between *miR-146a*$^{-/-}$ and WT platelets were nullified (Figures 6D and 6E).

In Thr-stimulated platelets, the addition of palmitate further accentuated the preceding differences in OCR observed after Thr injection ($p < 0.0001$, Figure 6C). Thus, Thr activation did not alter OCR in the presence of etomoxir (Figure 6F) as previously shown (Figure 4B). Collectively, our results demonstrate that miR-146a-deficient platelets promote a metabolic shift that favors FAO as an important energy source.

FA oxidation drives *miR-146a*$^{-/-}$ platelet hyperreactivity under flow

To assess whether augmented FAO contributes to hyperreactivity in *miR-146a*$^{-/-}$ platelets, whole blood previously labeled with $DiOC_6$ to label platelets was perfused into microfluidics channels coated with collagen under pathological (4,000 s^{-1}) and physiological (1,100 s^{-1}) arterial shear-stress conditions. *miR-146a*$^{-/-}$ platelets exhibited larger thrombi and more rapid thrombus formation than WT platelets under both pathological ($p = 0.0056$, Figure 6G) and physiological ($p = 0.038$; Figures 6H and S10; Video S1) flow conditions, confirming the prothrombotic potential of miR-146a deficiency. When whole blood was pretreated with etomoxir, *miR-146a*$^{-/-}$ and WT platelets demonstrated diminished ability to form aggregates under physiological flow, and the differences between them disappeared (Figure 6I). Hence, miR-146a deficiency in platelets leads to enhanced thrombus formation ability under flow, primarily fueled by increased FAO. Thus, these findings underscore the significance of FAO as a pivotal pathway for platelet activation and aggregation under both static (Figures 2 and 3) and flow (Figures 6G–6I) conditions.

miR-146a mimic therapy avoids mortality by reducing platelet aggregation

Following the characterization of miR-146a deficiency's role in platelet hyperreactivity *in vitro*, we examined its *in vivo* implications. Initially, primary hemostasis was assessed via tail bleeding assay, revealing that *miR-146a*$^{-/-}$ mice exhibited significantly lower bleeding volume ($p = 0.017$, Figure S11A) and slightly shorter bleeding times ($p = 0.280$, Figure S11B) compared to WT mice. Next, we used a model of systemic collagen/epinephrine-induced thrombosis that provokes platelet activation leading to pulmonary thromboembolism death.[19] This model is extensively used to understand the role played by platelet receptor and other effector molecules,[20,21] including miRNAs,[22] as well as the impact of antithrombotic drugs.[23] Platelet activation was induced by injecting collagen and epinephrine in WT and miR-146a knockout mice. *miR-146a*$^{-/-}$ mice displayed a significantly lower survival rate than WT mice (47.6% vs. 82.3%, $p = 0.024$, Figure 7A) post-pulmonary embolism (PE) induction. Correspondingly, the lungs of deficient mice exhibited significantly higher scores for perfusion defects than those of WT mice ($p = 0.008$, Figure 7B).

To restore or elevate miR-146a levels, miR-146a-deficient mice were treated with a miR-146a mimic oligonucleotide conjugated to a scavenger receptor/Toll-like receptor 9 agonist (C-miR-146a).[24] The systemic C-miR-146a administration resulted in a 213-fold increase in miR-146a levels in circulating platelets compared to WT basal levels ($p = 0.0006$, Figure S12). C-miR-146a and control scrambled RNA (C-scr) were intravenously injected for 3 days prior to PE induction. Importantly, miR-146a-deficient mice treated with C-miR-146a were fully protected against PE-induced mortality compared to *miR-146a*$^{-/-}$ mice treated with C-scr (100% vs. 44.4% survival rate, respectively, $p = 0.015$, Figure 7A) and did not present significant platelet aggregates in lungs ($p = 0.0055$; Figures 7C and 7D). Furthermore, we evaluated platelet reactivity in WT and *miR-146a*$^{-/-}$ mice injected with the oligonucleotide. The administration of C-miR-146a to *miR-146a*$^{-/-}$ mice reduces platelet reactivity in response to PAR4-AP, even decreasing their fibrinogen binding ability compared to WT (Figure 7E). However, C-miR-146a injection into WT mice did not alter the platelet activation or AC/C ratios (Figures 7E and 7F). Interestingly, C-miR-146a injection into miR-146a-deficient mice also decreased the AC/C ratios of platelets to levels similar to those of WT platelets (Figure 7F).

Thus, miR-146a deficiency in platelets induces a metabolic reprogramming favoring FAO, exacerbating their response and leading to a thrombotic phenotype with reduced survival rates in a PE model. Importantly, C-miR-146a mimic therapy reverses the platelet phenotype, offering a promising therapeutic approach for individuals with partial miR-146a deficiency due to miR-146a functional SNPs.

The role of miR-146a in platelet activation and aggregation *in vivo* was further confirmed using an opposite strategy involving the administration of anti-miR-146a to reduce levels of miR-146a (Figure S13). The survival rate of these WT mice injected with anti-miR-146a following systemic platelet activation induction was 50.0% vs. 88.9% in WT mice injected with anti-miR-scr (Figure S13).

(mean ± SD, n = 4–6 mice/group, two-tailed t test). (D) Representative immunofluorescence images (1,875 × 980) of the previous lung sections stained with FITC anti-CD41 antibody and DAPI for the nucleus. Scale bar 20 μm. (E) Platelets from WT and *miR-146a*$^{-/-}$ mice + miRNA therapy (C-scr or C-miR-146a mimic) were collected and studied by flow cytometry following PAR4-AP stimulation. (F) The acyl carnitine/carnitine (AC/C) ratio was also determined by Q Exactive Plus Hybrid Quadrupole-Orbitrap mass spectrometer in these platelets.

DISCUSSION

miRNAs influence several key cellular functions in various pathophysiological conditions.[25–27] In particular, miR-146a plays a pivotal role in modulating innate and adaptive immunity,[12,28,29] and the rs2431697-T allele, which reduces miR-146a expression by 50%,[10,17] has been associated with autoimmune diseases such as rheumatoid arthritis, systemic lupus erythematosus, or psoriasis.[30–32] Furthermore, our group has demonstrated that the rs2431697-TT genotype predicts the progression of myelofibrosis in a study involving 967 patients with myeloproliferative neoplasm.[33] Importantly, the presence of the rs2431697-T allele has been linked to the occurrence of CVEs in several thromboinflammatory diseases,[7,8,10] though the precise mechanisms remain elusive. While miRNAs are recognized as crucial elements in platelet biology,[34,35] no data were available concerning the role of miR-146a in platelet function and how its partial deficiency may contribute to CVEs in this setting.

We observed higher platelet activation in rs2431697-T patients with CAP compared to rs2431697-CC individuals. Patients with CAP with the rs2431697-T allele exhibited significantly elevated P-selectin exposure in resting platelets compared to rs2431697-CC on days 1 and 4–5 of hospitalization. Although the levels of miR-146a were not specifically measured in these patients, our data support that they may influence the activation status of platelets during the early stages of the disease based on the measurement of this miRNA in platelets from healthy subjects (Figure S1) and previous publications.[10,17,31] Intriguingly, the presence of the rs2431697-T allele was also associated with an increased incidence of CVE during the hospitalization of patients with CAP, highlighting the association between miR-146a levels and cardiovascular complications in a thromboinflammatory context. Additionally, platelets from rs2431697-TT healthy subjects were hyperreactive, particularly upon stimulation in static and flow conditions. Indeed, low miRNA levels have been associated with subthreshold pathological effects that only become apparent upon stress.[36,37] In the present study, our results show that in healthy individuals, the presence of the rs2431697-T allele only provokes a mild hyperreactive platelet phenotype when platelets are challenged with agonists, in contrast to patients with CAP, where platelet hyperactivation in rs2431697-T individuals is clear without the addition of exogenous activators, just induced by the pathological situation.

To clearly elucidate how miR-146a is implicated in platelet function, we performed our study in miR-146a-deficient mice. A thrombotic model based on widespread platelet activation was performed by administering collagen/epinephrine. Consistent with our findings in humans, miR-146a-deficient mice exhibited impaired survival and a higher lung perfusion defect score after systemic thrombosis induction compared to WT mice. Thus, the administration of miR-146a mimic, specifically targeting myeloid cells, conferred complete protection against death in these mice by avoiding platelet aggregate formation in lungs, reducing platelet activation, and restoring AC/C ratios. Recently, *in vivo* miR-146a mimic therapy has shown promise in repressing ovarian growth, sporadic colorectal cancer development, and myeloid leukemia progression through inflammation control.[24,38,39] Furthermore, miR-146a mimics have been tested in inflammatory conditions such as psoriasis, atherosclerosis, or aging.[40–42]

However, a theoretical limitation of miR-146a replacement therapy is the possibility of unintended repression of transcripts sharing seed-region complementarity, which could lead to unanticipated biological effects, especially upon systemic administration. In addition, chemical stabilization and conjugation strategies, while essential for *in vivo* use, may alter miRNA strand selection, RISC loading, or intracellular distribution. Importantly, several aspects of our C-miR146a mimic design were intended to minimize such risks: (1) the CpG(D19) conjugation enabled preferential uptake of C-miR146a by scavenger receptor/TLR9-expressing myeloid cells, substantially limiting exposure of non-target cell types (e.g., T cells) *in vivo*. This restriction is a key for off-target mitigation and reduces transcriptome-wide perturbations in irrelevant tissues. (2) Consistent with established design principles, chemical modifications were confined predominantly to the passenger strand (e.g., phosphorothioation and 2′-O-methylation) to preserve native guide-strand sequence properties and reduce the risk of altered seed-region behavior or aberrant strand usage. (3) The pharmacokinetic profile of C-miR146a resulted in transient target engagement,[24] thereby limiting prolonged or cumulative off-target effects. Our further optimization of the CpG-miR146 mimic strategy will include limited chemical modifications of both guide and passenger miRNA strands, as both can contribute to off-target effects as well as potency and durability of the on-target therapeutic effect. The off-target binding by the original vs. modified miRNA variants will be assessed using miR-eCLIP sequencing to directly identify bound target mRNAs.[43] We believe that our findings, together with ongoing advances in computational design and network-informed optimization,[44] support the continued development of miR-146a-based therapies with an increasingly favorable safety profile.

Importantly, the beneficial impact of miR-146a mimic therapy on other myeloid cells, such as neutrophils or monocytes, may also mitigate thromboinflammatory processes.[10,45] Our findings provide a rationale for the use of miR-146a mimic as an antiplatelet drug that drives metabolic regulation. Indeed, the role of platelet metabolism reprogramming in thrombotic diseases is a novel topic with a potential translational impact. Only one recent publication has demonstrated the link between platelet metabolic shift and its response in disease.[46]

Regarding metabolism and miR-146a, it has been reported that miR-146a targets dihydrolipoyl succinyltransferase (DLST) in cardiomyocytes. miR-146a deficiency rescued DLST expression, which was decreased in a model of pressure overload, counteracting cardiac hypertrophy and dysfunction while preserving oxidative fluxes in these cells.[47] *In silico* analysis indicated that miR-146a has potential targets in mitochondria; however, DLST, CPT1a, and other targets directly involved in FAO were not among them.[48] Regardless, we found that

miR-146a restrains OCR in platelets by directly or indirectly regulating *Cpt1a* and acyl-carnitine levels, subsequently affecting FAO and FA levels, as evidenced by metabolomics analysis. While the main energetic sources for platelet activation remain unclear, platelet function does not seem to be entirely compromised by the absence of a specific fuel, as platelets can easily switch between various sources.[49] OXPHOS and aerobic glycolysis have been identified as the main metabolic pathways to trigger and support platelet activation.[50,51] Lipid membrane remodeling has been shown to be crucial for megakaryocyte maturation, platelet formation, and platelet activation.[52,53] Indeed, cytoplasmic PLA_2 is involved in this FA mobilization that feeds TCA via FAO by acetyl-CoA production to attend to the energy demand for platelet activation.[54] Our data showed that the addition of palmitate, a long-chain FA, enhanced platelets' OCR in both genotypes, although miR-146a-deficient platelets exhibited a more pronounced increase. Thus, WT and miR-146a-deficient platelets displayed significantly impaired mitochondrial function, as well as activation and aggregation under flow, in the presence of etomoxir (CPT1a inhibitor), indicating that FA combustion is critical for maintaining platelet reactivity in both genotypes, with greater impairment observed in miR-146-deficient platelets. FAO inhibition with etomoxir completely abrogated hyperactivation in *miR-146a*$^{-/-}$ platelets to levels observed in WT platelets, as shown by microfluidics and OCR data. Taken together, these data demonstrate that exacerbated FAO driven by miR-146a deficiency leads to platelet hyperreactivity and a prothrombotic state. Previous studies have shown that FAO is fundamental for a complete human platelet activation; however, the concentrations of etomoxir commonly used ($\geq$10–100 μM) have strong off-target effects independent of CPT1A, causing oxidative stress in the cell.[18,55,56]

On the other hand, lipid synthesis also plays a role in platelet activation. Constitutive activation of acetyl-CoA carboxylase upregulates specific phospholipid pools and thromboxane A2 formation, thereby enhancing platelet response to collagen without impacting FAO.[57] In conditions of a high-fat diet, miR-146a deficiency did not affect hepatic mRNA expression of FA synthase or FA binding proteins. However, it did increase the expression of the phospholipid and cholesterol transporter *Abcg1*.[58] Conversely, no changes in glucose metabolism were detected,[58] which is consistent with our metabolomics data showing no significant alterations in glucose metabolism related to platelet activation in miR-146a-deficient platelets. In contrast, decreased FA levels combined with an increased AC/C ratio and *Cpt1a* expression indicate exacerbated FAO, which improved the ability of miR-146a-deficient platelets to respond to energetic demands. This was demonstrated by OCR measurement in the presence of various agonists, along with palmitate addition. Additionally, it has been suggested that CPT1A may influence mitochondrial morphology, inducing their fission when CPT1A expression increases in cancer cells.[59] However, we observed a mild and significant increase in mitochondrial mass, which translates into a greater number of mitochondria in *miR-146a*$^{-/-}$ platelets compared to WT platelets. Furthermore, these platelets also exhibited an increased α-granule count and accelerated shape changes in static conditions, supporting the observed thrombus growth and stability in the microfluidics assay.

Although we observed preferential uptake of the myeloid cell-targeted miR-146a mimic by platelets, which are the main players in the thrombus initiation and in the activation of other immune cells, the impact of this drug on other cells, such as monocytes and neutrophils, cannot be discarded. We showed the cellular mechanism of miR-146a through FAO together with an upregulation of *Cpt1a* as a potential cause of this metabolic dysregulation. However, given the pleiotropic effect of miRNAs, the search for additional targets that may help better define the molecular mechanism of miR-146a in relation to metabolism reprogramming is necessary in future studies. Finally, to demonstrate the role of the miR-SNPs, rs2431697, in the platelet phenotype, we use a real-world cohort that has some limitations, such as advanced age and comorbidities, including previous CVEs. However, the availability of such a large cohort with a thromboinflammatory disease in which platelet reactivity was measured during several days is unusual and of invaluable importance concerning the information that it can provide.

Taken together, these findings suggest that myeloid cell-targeted miR-146a mimic therapy could be a promising approach to treating patients with thromboinflammatory diseases, mitigating platelet hyperactivation as observed in patients with CAP. Moreover, platelets have important functions beyond hemostasis, as they interact with immune cells and possess immune functions themselves.[60,61] Therefore, increasing miR-146a levels may regulate platelet activation and the thromboinflammatory response, ultimately reducing cardiovascular risk. Overall, our data identify miR-146a as the only miRNA currently described to regulate platelet metabolism and, subsequently, its activation, supporting the idea that targeting metabolism as a therapeutic strategy toward more effective and safer antiplatelet treatments.

MATERIALS AND METHODS

Patients with CAP

The study received approval from the ethical committee of La Fe University and Polytechnic Hospital (2016/0486) and was conducted in accordance with the principles outlined in the 1964 Declaration of Helsinki and its subsequent amendments.

Consecutive patients admitted with CAP at La Fe University and Polytechnic Hospital were enrolled (n = 346). Circulating platelet P-selectin exposure was assessed on days 1, 4–5, and 30 after hospital admission in 163 patients by flow cytometry (EPICS-XL MLC, Beckman Coulter, Spain). Citrated whole blood was diluted with HBSS buffer, incubated (20 min, room temperature) with antibodies (CD61-PE, CD62-FITC, Immunostep, Salamanca, Spain), and further diluted. At least 10,000 platelets were counted, selected by size and forward scatter, and confirmed by CD61 positivity. Exposure of P-selectin was measured by CD62 binding and expressed as median fluorescence intensity (MFI). DNA was extracted from the

buffy coat using the Puregene Blood kit (Qiagen, Barcelona, Spain), and rs2431697 of miR-146a was genotyped using the TaqMan SNP Genotyping Assays C_26693319_10 (Thermo Fisher Scientific). The inclusion criteria of patients with CAP were a diagnosis of pneumonia based on a new radiologic infiltrate with at least two compatible clinical symptoms (dyspnea, cough, sputum production, chest pain, fever, chills, and confusion) and age $\geq$ 18 years. Exclusion criteria were admission in the previous 15 days, residence in a nursing home, immunosuppressive treatments or conditions, and human immunodeficiency virus infection. All patients who met the eligibility criteria or their next of kin provided written informed consent. Collected data included demographic variables, comorbidities, analytical parameters at admission, and prior treatments. Initial severity was assessed by the pneumonia severity index and the pneumonia severity score CURB65. The outcomes considered were length of stay (LOS), CVEs (in-hospital and 1 year), and mortality (in-hospital and 1 year). CVEs were previously defined.[62]

Briefly, the occurrence of CVEs was considered if any of the following appeared during hospitalization or in the 1 year follow-up: acute coronary syndrome (AMI or unstable angina), new or worsening heart failure, *de novo* or recurrent arrhythmia requiring hospital admission or emergency department care, cerebrovascular accident (stroke or transient ischemic attack), and/or venous thromboembolic disease (deep venous thrombosis and/or PE).

miR-146a quantification in platelets

Platelets from healthy individuals and platelets from WT mice and *miR-146a*$^{-/-}$ with or without the injection of oligonucleotides (C-miR-146a or C-scr, anti-miR-146a or anti-miR-scr) were washed with Tyrode's buffer (TB). Total RNA was isolated using TRIzol Reagent (Thermo Fisher Scientific, Madrid, Spain). miR-146a cDNA was synthesized using individual miRNA-specific RT primers (000468) and the TaqMan MicroRNA Reverse Transcription Kit (Thermo Fisher Scientific) and amplified using Premix ExTaq from Takara (Saint-Germain-en-Laye, France). miR-146a relative abundance was calculated using U6 (001141) and miR-451a (001973) to normalize for red blood cell contamination ($2^{-\Delta Ct(U6\text{-}miR146a)}/2^{-\Delta Ct(U6\text{-}miR451a)}$). Comparisons of means between groups were carried out using the one-tailed unpaired Mann-Whitney U test and a ROUT test (Q = 1%) to identify outliers.

Human platelet activation assay

Platelet activation was assessed using fibrinogen Alexa Fluor 488 (Thermo Fisher Scientific), PE anti-CD62P antibody (eBioscience, Madrid, Spain), and APC anti-mouse CD41a antibody (eBioscience). Whole blood was diluted in TB (20,000 plts/μL), and platelets were stimulated with various concentrations of agonists, including ADP (2.5 μM), TRAP-6 (12.5 μM), CRP (1 μg/mL), and PMA (100 nM). After 20 min of incubation at room temperature, reactions were stopped with 2% PFA. Data were acquired using a BD Accuri C6 Flow Cytometer (BD Biosciences, Madrid, Spain), and the MFI was calculated.

T-TAS

The healthy donor study (EST-08/20) was approved by CEIC-Hospital U. Morales Meseguer in accordance with the principles of the 1964 Declaration of Helsinki and its subsequent amendments. Peripheral whole blood from healthy donors was collected in benzylsulfonyl-d-arg-pro-4-amidinobenzylamide (BAPA) tubes. 300 μL were applied to PL chips coated with type I collagen in the T-TAS device following the manufacturer's instructions (Fujimori Kogyo, Tokyo, Japan). Changes in flow pressure were monitored to obtain parameters including OST, OT, and AUC.

Mice

Mouse care and handling were conducted in accordance with European Commission guidelines enforced in Spanish law under Real Decreto 53/2013 and were approved by the institutional ethics review board and Agriculture Department, CARM (A13180504).

Global *miR-146a*$^{-/-}$ mice (The Jackson Laboratory, Bar Harbor, ME) were backcrossed for >8 generations in a C57BL/6J background. WT littermates served as controls for comparative studies (Figure S14). All mice were 8–14 weeks old and of both sexes.

Mouse blood collection and washed platelet preparation

Blood was collected from mice under isoflurane anesthesia via the cava vein into acid citrate dextrose (ACD) (10%) or heparin (50 IU/mL, ROVI, Madrid, Spain), depending on the assay requirements. To isolate platelets, 1 mL of whole blood was diluted in TB at pH 6.8 (137 mM NaCl, 2 mM KCl, 12 mM $NaHCO_3$, 0.3 mM Na_2HPO_4, 1 mM $MgCl_2$, 5 mM HEPES, and 5.5 mM glucose) and centrifuged at 300 $\times$ *g* for 4 min. The platelet-rich fraction was collected into fresh tubes and centrifuged at 1,000 $\times$ *g* for 6 min after the addition of PGI_2 (1 μg/mL). The resulting platelet pellet was resuspended in TB (pH 7.4). Platelets were then counted and allowed to rest for 30–45 min before subsequent experiments.

Assessment of mouse platelet activation by flow cytometry

Platelet activation was assessed as described above. Samples were stimulated with various concentrations of different agonists (CRP, PAR4-AP, PMA, and ADP). After 20 min of incubation at room temperature, reactions were halted with 2% PFA. Data were acquired, and MFI for fibrinogen binding and P-selectin exposure was calculated.

FITC anti-mouse monoclonal antibodies were employed to detect platelet receptors: anti-CD41/CD61 (GPIIb/IIIa), CD42b (GPIb), CD49 (GPIa), anti-GPVI (Emfret Analytics, Eibelstadt, Germany), and anti-$P2Y_1$ receptor (Alomone, Jerusalem, Israel).

PS exposure (detected using Annexin V, Invitrogen) was monitored over time following stimulation with 0.5 IU/mL Thr (Merck, Barcelona, Spain) in washed platelets supplemented with 2.5 mM $CaCl_2$. Additionally, ROS generation was determined using 10 μM H_2DCFDA (Thermo Fisher Scientific) in the absence or presence of Thr. Samples were analyzed by flow cytometry.

Luciferase assay

PCR product (390 bp) containing a fragment of *P2ry1* 3′ UTR with miR-146a binding site was subcloned into luciferase reporter plasmid pMIR-REPORT (Life Technologies, Madrid, Spain) in HindIII/SpeI sites (F_GCGCGCACTAGTAATGACTGGAGATCT GGGAGA/R_GCGCGCAAGCTTTATGGAGTGCTGAGCTGT GG). A mutant of pMIR-REPORT-p2ry1 with a 2-nt deletion in the miR-146a-5p seed region-binding site was generated using QuikChange site-directed mutagenesis kit (Agilent Technologies, Santa Clara, CA) (F_CATGAGTCTTACCACTTGAGCT CTGAAAATCTCAAAAGAC/R_GTCTTTTGAGATTTTCAG AGCTCAAGTGGTAAGACTCATG). Sanger sequencing was performed to check for the deletion in the seed sequence. HEK293 T cells were co-transfected with 250 ng/well pMIR-REPORT, 25 ng/well of Renilla luciferase control plasmid (pRL-TK; Promega, Madison, WI), 100 nM C-miR146a mimic or C-scr, using Lipofectamine LTX (Life Technologies) according to the manufacturer's instructions. 24 h after transfection, firefly and renilla luciferase activities were measured using the Dual-Luciferase Reporter Assay (Promega, Madison, WI) in a SpectraMax iD3 luminometer (Molecular Devices, San Jose, CA).

Platelet spreading

Washed mouse platelets (20,000 cells/μL) were allowed to spread on fibrinogen-coated coverslips after stimulation with Thr (0.2 IU/mL) for 5, 15, 30, and 45 min at 37°C. Spread platelets were fixed with 2% PFA, permeabilized, and blocked with 2% BSA/0.1% Triton X-100 in PBS for 1 h. The primary antibody anti-tubulin β1 (1/500, Merck) was incubated for 1 h and washed. Subsequently, the coverslips were incubated with 10 μM Alexa Fluor 488 phalloidin (Thermo Fisher Scientific) and 1/1,000 secondary antibody goat anti-mouse IgG Alexa Fluor 546 (Invitrogen, Madrid, Spain). Platelets were visualized using a Stellaris confocal microscope (63×) (Leica Microsystems, Barcelona, Spain). Statistical analysis of the spread percentage of WT and miR-146a-deficient platelets observed at different spreading stages was performed using multiple unpaired *t* test.

F-actin polymerization

F-actin polymerization was determined as described.[63] Briefly, 3×10^5/μL washed platelets were incubated in TB or 0.1 IU/mL Thr (Merck) for 2 min. Then, the activation was stopped by 3.2% PFA in PHEM buffer for 10 min, and 10 μM phalloidin Alexa Fluor 488 (Thermo Fisher Scientific) was incubated in 0.1% Triton X-100 solution for 30 min, and platelets were analyzed by flow cytometry.

Clot retraction assay

Whole blood collected in 4% citrate was used to prepare platelet-rich plasma (PRP), which was adjusted to 4×10^8 platelets/mL in 200 μL TB + 2 mM $CaCl_2$. Platelets were then stimulated with 2 mg/mL fibrinogen (Merck) and 5 IU/mL of Thr and incubated at 37°C for up to 1.5 h, along with 4 μL of erythrocytes to enhance the contrast of the clot.

Transmission electron microscopy

Isolated PRP was fixed with 5% glutaraldehyde (Bio-Rad, Alcobendas, Spain) for 45 min at room temperature. After washing, a post-fixation step was performed by incubating them with 1% osmium tetroxide (Merck) for 2 h at 4°C, followed by treatment with 2% uranyl acetate veronal for 2 h at 4°C. Samples were then dehydrated with a series of ethanol solutions and propylene oxide before being embedded in Epon (Taab Laboratories, Reading, UK). Embedded samples were sectioned with an Ultracut E ultramicrotome (Reichert, Vienna, Austria) and stained with uranyl acetate and lead citrate (Merck). Platelet sections were observed with a JEOL 1011 transmission electron microscope (JEOL USA, Peabody, MA, USA). For the analysis, 150 randomly selected platelet sections were used to measure platelet size and the number of α-granules per platelet. The number of mitochondria per platelet was also quantified in randomly selected images (40–50 platelets per mouse).

Seahorse extracellular flux

Washed platelets were seeded in 96-well plates for Seahorse extracellular flux analysis (Agilent, Madrid, Spain). Platelets were diluted to 1×10^7 in XF DMEM media supplemented with 1 mM pyruvate, 5.5 mM glucose, and 2 mM L-glutamine (pH 7.4). Various stimulants, including Thr (0.5 IU/mL), PAR-4-AP (500 μM), PMA (200 nM), and CRP (20 μg/mL), were injected to stimulate platelets before subsequent injections of oligomycin, FCCP, rotenone, and antimycin A.

A Mito stress test was also performed with the addition of BSA-palmitate (150 μM) and BSA (FA-free) alone as a control or 5 μM etomoxir (Merck), with etomoxir being incubated for 30 min before running at 37°C.

Mitochondrial mass quantification

Mitochondria in washed platelets were measured using 100 nM MitoTracker Green (Invitrogen) labeling by flow cytometry or anti-TOM20 antibody (Cell Signaling Tech., Barcelona, Spain) by western blot. Densitometry of Tom20/β-actin was calculated using Image Lab software (Bio-Rad).

Untargeted metabolomics

Washed platelets ($1–2 \times 10^7$) were stimulated with Thr (0.5 IU/mL), PAR4-AP (500 μM), and CRP (20 μg/mL) for 5 min in TB. Then, methanol:acetonitrile (50:50) + 0.5% formic acid was added, vortexed, incubated for 3 min in ice, and neutralized with 15% ammonium bicarbonate. Samples were stored at −80°C and analyzed using a quadrupole-orbitrap mass spectrometer (Q Exactive, Thermo Fisher Scientific) coupled to hydrophilic interaction chromatography via electrospray ionization. Liquid chromatography (LC) separation was on a XBridge BEH Amide column (2.1 × 150 mm, 2.5 μm particle size; Waters, Milford, MA) using a gradient of solvent A (20 mM ammonium acetate, 20 mM ammonium hydroxide in 95:5 water:acetonitrile [pH 9.45]) and solvent B (acetonitrile). The flow rate was 150 μL/min, the column temperature was 25°C, the autosampler temperature was 5°C, and the injection volume was 10 μL. The LC

gradient was 0 min, 90% B; 2 min, 85% B; 3 min, 75% B; 7 min, 75% B; 8 min, 70% B; 9 min, 70% B; 10 min, 50% B; 12 min, 50% B; 13 min, 25% B; 14 min, 25% B; 16 min, 0% B; 21 min, 0% B; 22 min, 90% B; and 25 min, 90% B. The autosampler temperature was 5°C, and the injection volume was 10 μL. The mass spectrometer was operated in negative ion mode to scan from *m/z* 70 to 700 at 1 Hz and a resolving power of 140,000 and an automatic gain control (AGC) target of 3×10^6 and from *m/z* 650 to 1,200 at 1 Hz and a resolving power of 140,000, an AGC target of 5×10^6, and a maximum injection time of 500 ms. Data were acquired in centroid mode.[64] Data were processed with ElMaven (v.0.0.12.1, Elucidata),[65] using windows of 10 ppm and retention time as previously determined using a library of standards.[66] Raw data of metabolomics were deposited in Zenodo (https://zenodo.org) under accession number 13693362.

Untargeted metabolomics analysis in mouse serum

Serum samples were extracted with 40 volumes of methanol:water (80:20) and incubated for at least 20 min at −80°C. Before LC-mass spectrometry (LC-MS) analysis, the samples were centrifuged twice (4°C, 15 min, 15,000 × *g*), and the clean supernatants were transferred to LC vials. Sample analysis was performed using a quadrupole-orbitrap mass spectrometer (Q Exactive, Thermo Fisher Scientific) coupled to hydrophilic interaction chromatography via electrospray ionization as previously described in the main materials and methods section.

RNA isolation and quantitative real-time PCR

Total RNA was extracted using TRIzol reagent from pooled whole-blood samples obtained from three mice. Reverse transcription (RT) was performed according to the manufacturer's instructions of MultiScribe Reverse Transcriptase (Invitrogen). *Cpt1a* expression (F_CCGGACTCCGCTCGCT/R_CATCTTGAGTGGTGACCGAGT) was detected by quantitative real-time PCR using a Real Time LC480 PCR system (Roche Applied Science). The $2^{-\Delta Ct}$ method was employed to calculate the relative abundance of mRNA compared with the endogenous control (GADPH).

Microfluidics assays

Microfluidics device channels were coated with collagen (100 μg/mL, Collagen Reagens HORM Suspension, Takeda, Linz, Austria) and incubated for 1 h, then blocked with a solution of 3% BSA FA-free (Merck) in PBS without Ca^{2+} and Mg^{2+}, followed by washing. Mouse blood was collected from the cava vein in heparin (50 IU/mL), and $DiOC_6$ (2 μM) was used to label platelets. The blood was then perfused into the microchannels at two shear rates: 1,100 and 4,000 s^{-1}. Platelet adhesion and thrombus formation were visualized and recorded using a fluorescence microscope (Leica Microsystems). Thrombus volumes were calculated using Imaris software (Oxford Instruments, High Wycombe, UK). Additionally, whole blood was tested after incubation with etomoxir (5 μM) for 30 min at 37°C.

Tail bleeding assay

Mice were anesthetized, and their tail tips were amputated (5 mm) before being immediately immersed in saline at 37°C. Bleeding was monitored for a maximum of 10 min or until cessation (defined as no re-bleeding within 1 min). The bleeding volume was determined by the hemoglobin (Hb) content of the collected blood in saline tubes. These tubes were then centrifuged, and erythrocytes were lysed using Qiagen's lysis solution (Qiagen, Madrid, Spain). Absorbance was measured at 575 nm using a microplate reader (Biotek Synergy, Agilent Technologies). For calculation purposes, a standard curve generated with known blood Hb concentrations was utilized.

Systemic thrombosis

Mice were anesthetized with a ketamine (100 mg/kg) and xylazine (10 mg/kg) cocktail. Collagen (0.4 mg/kg, Chrono-log, Oxford, UK) and 60 μg/kg of epinephrine (Diagnostica Stago, Barcelona, Spain) were retro-orbitally administered after dilution in saline. Time to death was monitored for 30 min, with death defined as respiratory arrest persisting for at least 2 min. Additionally, mice were used to evaluate pulmonary occlusion by perfusing them with 1% Evans Blue (Merck) via the right ventricle. Lungs were photographed and blindly scored by two independent observers using a reference scale (0 = perfusion; 4 = complete occlusion) (Figure 7B). The intraclass correlation coefficient (ICC) was used to assess the agreement between the two observers.

C-miR146a mimic therapy and anti-miR-146a administration

The design and characterization of the C-miR146a mimic oligonucleotide were described before.[24] The oligonucleotides at a dose of 10 mg/kg (100 μL diluted in saline solution) were intravenously injected for 3 days before inducing PE in *miR-146a*$^{-/-}$ mice. On the third day, 2 h after the injection, collagen and epinephrine were administered to induce PE. The time to death was recorded for 30 min, and then the lungs were fixed with 4% PFA. Subsequently, specimens were washed with PBS, mounted on aluminum stubs, and embedded with OCT compound (Tissue-Tek). The lungs were cut into 10 μm sections using a cryostat (Leica CM3050S, Leica Microsystems) at −20°C, and platelets were labeled for immunofluorescence analysis (CD41a, BioLegend, CA, USA) and visualized using a Stellaris confocal microscope (63×) (Leica Microsystems, Barcelona, Spain).

For a different assay, the C-miR146a and the scramble (C-scr) mimic oligonucleotides were intravenously injected as described above. In this case, whole blood was collected in heparin. The diluted whole blood was prepared as previously described, and platelet activation was studied after PAR4-AP activation. The remaining blood was used to wash platelets for metabolomics studies as described earlier.

The oligonucleotides of anti-miR-146a or -scr at a dose of 5 mg/kg (100 μL diluted in saline solution) were intravenously injected for 2 days in WT mice before inducing a systemic thrombosis. On the second day, 2 h after the injection, collagen and epinephrine were administered to induce PE. The time to death was recorded for 30 min, and then the lungs were fixed with 4% PFA for hematoxylin-eosin staining.

Statistical analysis

The statistical analysis was conducted using GraphPad Prism 9 (GraphPad Software) and SPSS 22.0 for Windows (IBM). Continuous variables were reported as mean ± SD or median (interquartile range [IQR]), while categorical variables were expressed as percentages. For comparisons of categorical variables between groups, the χ^2 test was employed. Numerical variables were compared using either a two-tailed Student's *t* test or a Mann-Whitney U test, depending on the distribution of the data. In cases involving multiple comparisons, either an ordinary one-way ANOVA or a Kruskal-Wallis test was utilized. $p < 0.05$ were considered statistically significant, with significance levels indicated as follows: $^{*}p < 0.05$, $^{**}p < 0.01$, $^{***}p < 0.001$, and $^{****}p < 0.0001$. Additionally, the Hardy-Weinberg equilibrium was assessed for the genotype frequencies in the CAP cohort. Unsupervised multivariate analysis was performed by PCA on log-transformed, mean-centered, and unit-variance-scaled data using Metaboanalyst.

DATA AND CODE AVAILABILITY

Raw data of metabolomics were deposited in Zenodo (https://zenodo.org) under accession number 13693362. Further information and requests for resources and reagents should be directed and will be fulfilled by the lead contact, Constantino Martínez (constant@um.es).

ACKNOWLEDGMENTS

This study has been funded by Instituto de Salud Carlos III (ISCIII) through the projects "PI20/00136" and "PI23/00027" and co-funded by the European Union; Fundación Séneca - Agencia de Ciencia y Tecnología de la Región de Murcia (22104/PI/22, 23022/GERM/25, and 23054/GERM/25); Sociedad Española de Trombosis y Hemostasia (López Borrasca 2020-SETH); MICIU/AEI/10.13039/501100011033 and FEDER, UE (PID2022-137251OA-I00); and contracts from ISCIII (S.A.: CD18/00044 and CP21/00053 from ISCIII, RYC2023-043714-I from MICIU/AEI/10.13039/501100011033, and FSE+ and ISTH-EHA training fellowship 2019; L.Z.-M.: FI21/00065); SETH (S.C.-T.), and Conselleria de Sanidad Universal y Salud Pública, Generalitat Valenciana (J.C.G.-C.: DEI-01/20-C). We would like to thank María García and Teresa Coronado from the Microscopy Core (Universidad de Murcia), the Genotoul Imaging facilities (INSERM U1297, Toulouse), Ernesto Cuenca-Zamora (IMIB Pascual Parrilla), Nathaly Freire-Sánchez, and Beatriz Martínez-García for their excellent technical assistance. The authors also thank the nurses from Centro Regional de Hemodonación for the blood collection, as well as healthy donors and patients with CAP for their kind participation in the study.

AUTHOR CONTRIBUTIONS

S.A. designed and performed the research, analyzed the data, and drafted the manuscript. J.C.G.-C., L.Z.-M., and A.B.A. designed and performed the research and analyzed the data. P.J.G.-C. and N.G.-B. performed the research. P.G.-J., A. Latorre, and R.M. recruited the patients and analyzed the clinical data. M.L.L. and B.P. critically revised the manuscript and analyzed the data. J.R. designed the research, analyzed the data, and critically revised the manuscript. A. Lahoz designed the research and analyzed the data. M.K. contributed vital new reagents and critically revised the manuscript. R.G.-C. and C.M. supervised and designed the research and drafted the manuscript. The order of the co-first authors was assigned based on their contributions to the study. All authors approved the final version.

DECLARATION OF INTERESTS

The authors declare no competing interests.

SUPPLEMENTAL INFORMATION

Supplemental information can be found online at https://doi.org/10.1016/j.ymthe.2026.07.019.

REFERENCES

1. Roth, G.A., Mensah, G.A., Johnson, C.O., Addolorato, G., Ammirati, E., Baddour, L.M., Barengo, N.C., Beaton, A.Z., Benjamin, E.J., Benziger, C.P., et al. (2020). Global Burden of Cardiovascular Diseases and Risk Factors, 1990-2019: Update From the GBD 2019 Study. J. Am. Coll. Cardiol. *76*, 2982–3021. https://doi.org/10.1016/j.jacc.2020.11.010.

2. Martin, J.F., Kristensen, S.D., Mathur, A., Grove, E.L., and Choudry, F.A. (2012). The causal role of megakaryocyte–platelet hyperactivity in acute coronary syndromes. Nat. Rev. Cardiol. *9*, 658–670. https://doi.org/10.1038/nrcardio.2012.131.

3. Gawaz, M., Geisler, T., and Borst, O. (2023). Current concepts and novel targets for antiplatelet therapy. Nat. Rev. Cardiol. *20*, 583–599. https://doi.org/10.1038/s41569-023-00854-6.

4. Metharom, P., Berndt, M.C., Baker, R.I., and Andrews, R.K. (2015). Current state and novel approaches of antiplatelet therapy. Arterioscler. Thromb. Vasc. Biol. *35*, 1327–1338. https://doi.org/10.1161/ATVBAHA.114.303413.

5. Gachet, C. (2015). Antiplatelet drugs: which targets for which treatments? J. Thromb. Haemost. *13*, S313–S322. https://doi.org/10.1111/jth.12947.

6. Harris, J., Pouwels, K.B., Johnson, T., Sterne, J., Pithara, C., Mahadevan, K., Reeves, B., Benedetto, U., Loke, Y., Lasserson, D., et al. (2023). Bleeding risk in patients prescribed dual antiplatelet therapy and triple therapy after coronary interventions: the ADAPTT retrospective population-based cohort studies. Health Technol. Assess. *27*, 1–257. https://doi.org/10.3310/MNJY9014.

7. Roldán, V., Arroyo, A.B., Salloum-Asfar, S., Manzano-Fernández, S., García-Barberá, N., Marín, F., Vicente, V., González-Conejero, R., and Martínez, C. (2014). Prognostic role of MIR146A polymorphisms for cardiovascular events in atrial fibrillation. Thromb. Haemost. *112*, 781–788. https://doi.org/10.1160/th14-01-0092.

8. Wang, Y., Wang, X., Li, Z., Chen, L., Zhou, L., Li, C., and Ouyang, D.S. (2017). Two Single Nucleotide Polymorphisms (rs2431697 and rs2910164) of miR-146a Are Associated with Risk of Coronary Artery Disease. Int. J. Environ. Res. Public Health *14*, 514. https://doi.org/10.3390/ijerph14050514.

9. Arroyo, A.B., de los Reyes-García, A.M., Rivera-Caravaca, J.M., Valledor, P., García-Barberá, N., Roldán, V., Vicente, V., Martínez, C., and González-Conejero, R. (2018). MiR-146a Regulates Neutrophil Extracellular Trap Formation That Predicts Adverse Cardiovascular Events in Patients With Atrial Fibrillation. Arterioscler. Thromb. Vasc. Biol. 38, 892–902.

10. Arroyo, A.B., Fernández-Pérez, M.P., del Monte, A., Águila, S., Méndez, R., Hernández-Antolín, R., García-Barber, N., de los Reyes-García, A.M., González-Jiménez, P., Arcas, M.I., et al. (2021). miR-146a is a pivotal regulator of neutrophil extracellular trap formation promoting thrombosis. Haematologica *106*, 1636–1646. https://doi.org/10.3324/haematol.2019.240226.

11. Taganov, K.D., Boldin, M.P., Chang, K.J., and Baltimore, D. (2006). NF-kappaB-dependent induction of microRNA miR-146, an inhibitor targeted to signaling proteins of innate immune responses. Proc. Natl. Acad. Sci. USA *103*, 12481–12486. https://doi.org/10.1073/pnas.0605298103.

12. Boldin, M.P., Taganov, K.D., Rao, D.S., Yang, L., Zhao, J.L., Kalwani, M., Garcia-Flores, Y., Luong, M., Devrekanli, A., Xu, J., et al. (2011). miR-146a is a significant brake on autoimmunity, myeloproliferation, and cancer in mice. J. Exp. Med. *208*, 1189–1201. https://doi.org/10.1084/jem.20101823.

13. Zhou, Y., Abraham, S., Andre, P., Edelstein, L.C., Shaw, C.A., Dangelmaier, C.A., Tsygankov, A.Y., Kunapuli, S.P., Bray, P.F., and McKenzie, S.E. (2015). Anti-miR-148a regulates platelet FcγRIIA signaling and decreases thrombosis in vivo in mice. Blood *126*, 2871–2881. https://doi.org/10.1182/blood-2015-02-631135.

14. Elgheznawy, A., Shi, L., Hu, J., Wittig, I., Laban, H., Pircher, J., Mann, A., Provost, P., Randriamboavonjy, V., and Fleming, I. (2015). Dicer cleavage by calpain determines platelet microRNA levels and function in diabetes. Circ. Res. *117*, 157–165. https://doi.org/10.1161/CIRCRESAHA.117.305784.

15. Kaudewitz, D., Skroblin, P., Bender, L.H., Barwari, T., Willeit, P., Pechlaner, R., Sunderland, N.P., Willeit, K., Morton, A.C., Armstrong, P.C., et al. (2016). Association of MicroRNAs and YRNAs With Platelet Function. Circ. Res. *118*, 420–432. https://doi.org/10.1161/CIRCRESAHA.114.305663.

16. Arroyo, A.B., Águila, S., Fernández-Pérez, M.P., Reyes-García, A.M.d.L., Reguilón-Gallego, L., Zapata-Martínez, L., Vicente, V., Martínez, C., and González-Conejero,

R. (2021). miR-146a in Cardiovascular Diseases and Sepsis: An Additional Burden in the Inflammatory Balance? Thromb. Haemost. *121*, 1138–1150. https://doi.org/10.1055/a-1342-3648.

17. Löfgren, S.E., Frostegård, J., Truedsson, L., Pons-Estel, B.A., D'Alfonso, S., Witte, T., Lauwerys, B.R., Endreffy, E., Kovács, L., Vasconcelos, C., et al. (2012). Genetic association of miRNA-146a with systemic lupus erythematosus in Europeans through decreased expression of the gene. Genes Immun. *13*, 268–274. https://doi.org/10.1038/gene.2011.84.

18. O'Connor, R.S., Guo, L., Ghassemi, S., Snyder, N.W., Worth, A.J., Weng, L., Kam, Y., Philipson, B., Trefely, S., Nunez-Cruz, S., et al. (2018). The CPT1a inhibitor, etomoxir induces severe oxidative stress at commonly used concentrations. Sci. Rep. *8*, 6289. https://doi.org/10.1038/s41598-018-24676-6.

19. Westrick, R.J., Winn, M.E., and Eitzman, D.T. (2007). Murine models of vascular thrombosis (Eitzman series). Arterioscler. Thromb. Vasc. Biol. *27*, 2079–2093. https://doi.org/10.1161/ATVBAHA.107.142810.

20. Nieswandt, B., Schulte, V., Bergmeier, W., Mokhtari-Nejad, R., Rackebrandt, K., Cazenave, J.P., Ohlmann, P., Gachet, C., and Zirngibl, H. (2001). Long-term antithrombotic protection by in vivo depletion of platelet glycoprotein VI in mice. J. Exp. Med. *193*, 459–469. https://doi.org/10.1084/jem.193.4.459.

21. Požgajová, M., Sachs, U.J.H., Hein, L., and Nieswandt, B. (2006). Reduced thrombus stability in mice lacking the alpha2A-adrenergic receptor. Blood *108*, 510–514. https://doi.org/10.1182/blood-2005-12-4835.

22. Rowley, J.W., Chappaz, S., Corduan, A., Chong, M.M.W., Campbell, R., Khoury, A., Manne, B.K., Wurtzel, J.G.T., Michael, J.V., Goldfinger, L.E., et al. (2016). Dicer1-mediated miRNA processing shapes the mRNA profile and function of murine platelets. Blood *127*, 1743–1751. https://doi.org/10.1182/blood-2015-07-661371.

23. Hechler, B., Magnenat, S., Zighetti, M.L., Kassack, M.U., Ullmann, H., Cazenave, J.P., Evans, R., Cattaneo, M., and Gachet, C. (2005). Inhibition of platelet functions and thrombosis through selective or nonselective inhibition of the platelet P2 receptors with increasing doses of NF449 [4,4',4'',4'''-(carbonylbis(imino-5,1,3-benzenetriylbis-(carbonylimino)))tetrakis-benzene-1,3-disulfonic acid octasodium salt]. J. Pharmacol. Exp. Ther. *314*, 232–243. https://doi.org/10.1124/jpet.105.084673.

24. Su, Y.L., Wang, X., Mann, M., Adamus, T.P., Wang, D., Moreira, D.F., Zhang, Z., Ouyang, C., He, X., Zhang, B., et al. (2020). Myeloid cell-targeted miR-146a mimic inhibits NF-κB-driven inflammation and leukemia progression in vivo. Blood *135*, 167–180. https://doi.org/10.1182/blood.2019002045.

25. Rippo, M.R., Olivieri, F., Monsurrò, V., Prattichizzo, F., Albertini, M.C., and Procopio, A.D. (2014). MitomiRs in human inflamm-aging: a hypothesis involving miR-181a, miR-34a and miR-146a. Exp. Gerontol. *56*, 154–163. https://doi.org/10.1016/j.exger.2014.03.002.

26. Caravia, X.M., and López-Otín, C. (2015). Regulatory Roles of miRNAs in Aging. Adv. Exp. Med. Biol. *887*, 213–230. https://doi.org/10.1007/978-3-319-22380-3_11.

27. Agbu, P., and Carthew, R.W. (2021). MicroRNA-mediated regulation of glucose and lipid metabolism. Nat. Rev. Mol. Cel Biol. *22*, 425–438. https://doi.org/10.1038/s41580-021-00354-w.

28. Etzrodt, M., Cortez-Retamozo, V., Newton, A., Zhao, J., Ng, A., Wildgruber, M., Romero, P., Wurdinger, T., Xavier, R., Geissmann, F., et al. (2012). Regulation of monocyte functional heterogeneity by miR-146a and Relb. Cell Rep. *1*, 317–324. https://doi.org/10.1016/j.celrep.2012.02.009.

29. Yang, L., Boldin, M.P., Yu, Y., Liu, C.S., Ea, C.K., Ramakrishnan, P., Taganov, K.D., Zhao, J.L., and Baltimore, D. (2012). miR-146a controls the resolution of T cell responses in mice. J. Exp. Med. *209*, 1655–1670. https://doi.org/10.1084/jem.20112218.

30. Zhang, L.L., Wu, X.X., Wang, X.F., Di, D.S., Huang, Q., Liu, R.S., Shuai, Z.W., Ye, D.Q., and Leng, R.X. (2021). Genetic variant in microRNA-146a gene is associated with risk of rheumatoid arthritis. Ann. Med. *53*, 824–829. https://doi.org/10.1080/07853890.2021.1933163.

31. Hou, G., Harley, I.T.W., Lu, X., Zhou, T., Xu, N., Yao, C., Qin, Y., Ouyang, Y., Ma, J., Zhu, X., et al. (2021). SLE non-coding genetic risk variant determines the epigenetic dysfunction of an immune cell specific enhancer that controls disease-critical microRNA expression. Nat. Commun. *12*, 135. https://doi.org/10.1038/s41467-020-20460-1.

32. Muñoz-Aceituno, E., Butrón-Bris, B., Ovejero-Benito, M.C., Sahuquillo-Torralba, A., Baniandrés Rodríguez, O., Herrera-Acosta, E., Rivera-Diaz, R., Ferran, M., Sánchez-Carazo, J.L., Riera-Monroig, J., et al. (2024). Pharmacogenetic biomarkers for secukinumab response in psoriasis patients in real-life clinical practice. J. Eur. Acad. Dermatol. Venereol. *38*, 1783–1790. https://doi.org/10.1111/jdv.19782.

33. Ferrer-Marín, F., Arroyo, A.B., Bellosillo, B., Cuenca, E.J., Zamora, L., Hernández-Rivas, J.M., Hernández-Boluda, J.C., Fernandez-Rodriguez, C., Luño, E., García Hernandez, C., et al. (2020). miR-146a rs2431697 identifies myeloproliferative neoplasm patients with higher secondary myelofibrosis progression risk. Leukemia *34*, 2648–2659. https://doi.org/10.1038/s41375-020-0767-3.

34. Lindsay, C.R., and Edelstein, L.C. (2016). MicroRNAs in Platelet Physiology and Function. Semin. Thromb. Hemost. *42*, 215–222. https://doi.org/10.1055/s-0035-1570077.

35. Bhatlekar, S., Manne, B.K., Basak, I., Edelstein, L.C., Tugolukova, E., Stoller, M.L., Cody, M.J., Morley, S.C., Nagalla, S., Weyrich, A.S., et al. (2020). miR-125a-5p regulates megakaryocyte proplatelet formation via the actin-bundling protein L-plastin. Blood *136*, 1760–1772. https://doi.org/10.1182/blood.2020005230.

36. Mendell, J.T., and Olson, E.N. (2012). MicroRNAs in stress signaling and human disease. Cell *148*, 1172–1187. https://doi.org/10.1016/j.cell.2012.02.005.

37. Chivukula, R.R., Shi, G., Acharya, A., Mills, E.W., Zeitels, L.R., Anandam, J.L., Abdelnaby, A.A., Balch, G.C., Mansour, J.C., Yopp, A.C., et al. (2014). An essential mesenchymal function for miR-143/145 in intestinal epithelial regeneration. Cell *157*, 1104–1116. https://doi.org/10.1016/j.cell.2014.03.055.

38. Garo, L.P., Ajay, A.K., Fujiwara, M., Gabriely, G., Raheja, R., Kuhn, C., Kenyon, B., Skillin, N., Kadowaki-Saga, R., Saxena, S., and Murugaiyan, G. (2021). MicroRNA-146a limits tumorigenic inflammation in colorectal cancer. Nat. Commun. *12*, 2419. https://doi.org/10.1038/s41467-021-22641-y.

39. Chen, R., Coleborn, E., Bhavsar, C., Wang, Y., Alim, L., Wilkinson, A.N., Tran, M.A., Irgam, G., Atluri, S., Wong, K., et al. (2023). miR-146a inhibits ovarian tumor growth in vivo via targeting immunosuppressive neutrophils and enhancing CD8+ T cell infiltration. Mol. Ther. Oncolytics *31*, 100725. https://doi.org/10.1016/j.omto.2023.09.001.

40. Li, K., Ching, D., Luk, F.S., and Raffai, R.L. (2015). Apolipoprotein E enhances microRNA-146a in monocytes and macrophages to suppress nuclear factor-κB-driven inflammation and atherosclerosis. Circ. Res. *117*, e1–e11. https://doi.org/10.1161/CIRCRESAHA.117.305844.

41. Srivastava, A., Nikamo, P., Lohcharoenkal, W., Li, D., Meisgen, F., Xu Landén, N., Ståhle, M., Pivarcsi, A., and Sonkoly, E. (2017). MicroRNA-146a suppresses IL-17-mediated skin inflammation and is genetically associated with psoriasis. J. Allergy Clin. Immunol. *139*, 550–561. https://doi.org/10.1016/j.jaci.2016.07.025.

42. Gong, H., Chen, H., Xiao, P., Huang, N., Han, X., Zhang, J., Yang, Y., Li, T., Zhao, T., Tai, H., et al. (2022). miR-146a impedes the anti-aging effect of AMPK via NAMPT suppression and NAD+/SIRT inactivation. Signal. Transduct. Target. Ther. *7*, 66. https://doi.org/10.1038/s41392-022-00886-3.

43. Manakov, S.A., Shishkin, A.A., Yee, B.A., Shen, K.A., Cox, D.C., Park, S.S., Foster, H.M., Chapman, K.B., Yeo, G.W., and Van Nostrand, E.L. (2022). Scalable and deep profiling of mRNA targets for individual microRNAs with chimeric eCLIP. Preprint at bioRxiv. https://doi.org/10.1101/2022.02.13.480296.

44. Bereczki, Z., Benczik, B., Balogh, O.M., Marton, S., Puhl, E., Pétervári, M., Váczy-Földi, M., Papp, Z.T., Makkos, A., Glass, K., et al. (2025). Mitigating off-target effects of small RNAs: conventional approaches, network theory and artificial intelligence. Br. J. Pharmacol. *182*, 340–379. https://doi.org/10.1111/bph.17302.

45. Cheng, H.S., Besla, R., Li, A., Chen, Z., Shikatani, E.A., Nazari-Jahantigh, M., Hammoutène, A., Nguyen, M.A., Geoffrion, M., Cai, L., et al. (2017). Paradoxical Suppression of Atherosclerosis in the Absence of microRNA-146a. Circ. Res. *121*, 354–367. https://doi.org/10.1161/CIRCRESAHA.116.310529.

46. He, F., Laranjeira, A.B., Kong, T., Lin, S., Ashworth, K.J., Liu, A., Lasky, N.M., Fisher, D.A., Cox, M.J., Fulbright, M.C., et al. (2024). Multiomic profiling reveals metabolic alterations mediating aberrant platelet activity and inflammation in myeloproliferative neoplasms. J. Clin. Invest. *134*, 172256. https://doi.org/10.1172/JCI172256.

47. Heggermont, W.A., Papageorgiou, A.P., Quaegebeur, A., Deckx, S., Carai, P., Verhesen, W., Eelen, G., Schoors, S., Van Leeuwen, R., Alekseev, S., et al. (2017). Inhibition of MicroRNA-146a and Overexpression of Its Target Dihydrolipoyl

Succinyltransferase Protect Against Pressure Overload-Induced Cardiac Hypertrophy and Dysfunction. Circulation *136*, 747–761. https://doi.org/10.1161/circulationaha.116.024171.

48. Dasgupta, N., Peng, Y., Tan, Z., Ciraolo, G., Wang, D., and Li, R. (2015). miRNAs in mtDNA-less cell mitochondria. Cell Death Discov. *1*, 15004. https://doi.org/10.1038/cddiscovery.2015.4.

49. Kulkarni, P.P., Ekhlak, M., and Dash, D. (2023). Energy metabolism in platelets fuels thrombus formation: Halting the thrombosis engine with small-molecule modulators of platelet metabolism. Metabolism *145*, 155596. https://doi.org/10.1016/j.metabol.2023.155596.

50. Ravi, S., Chacko, B., Sawada, H., Kramer, P.A., Johnson, M.S., Benavides, G.A., O'Donnell, V., Marques, M.B., and Darley-Usmar, V.M. (2015). Metabolic plasticity in resting and thrombin activated platelets. PLoS One *10*, e0123597. https://doi.org/10.1371/journal.pone.0123597.

51. Aibibula, M., Naseem, K.M., and Sturmey, R.G. (2018). Glucose metabolism and metabolic flexibility in blood platelets. J. Thromb. Haemost. *16*, 2300–2314. https://doi.org/10.1111/jth.14274.

52. de Jonckheere, B., Kollotzek, F., Münzer, P., Göb, V., Fischer, M., Mott, K., Coman, C., Troppmair, N.N., Manke, M.C., Zdanyte, M., et al. (2023). Critical shifts in lipid metabolism promote megakaryocyte differentiation and proplatelet formation. Nat. Cardiovasc. Res. *2*, 835–852. https://doi.org/10.1038/s44161-023-00325-8.

53. Barrachina, M.N., Pernes, G., Becker, I.C., Allaeys, I., Hirsch, T.I., Groeneveld, D.J., Khan, A.O., Freire, D., Guo, K., Carminita, E., et al. (2023). Efficient megakaryopoiesis and platelet production require phospholipid remodeling and PUFA uptake through CD36. Nat. Cardiovasc. Res. *2*, 746–763. https://doi.org/10.1038/s44161-023-00305-y.

54. Slatter, D.A., Aldrovandi, M., O'Connor, A., Allen, S.M., Brasher, C.J., Murphy, R.C., Mecklemann, S., Ravi, S., Darley-Usmar, V., and O'Donnell, V.B. (2016). Mapping the Human Platelet Lipidome Reveals Cytosolic Phospholipase A2 as a Regulator of Mitochondrial Bioenergetics during Activation. Cell Metab. *23*, 930–944. https://doi.org/10.1016/j.cmet.2016.04.001.

55. Yao, C.H., Liu, G.Y., Wang, R., Moon, S.H., Gross, R.W., and Patti, G.J. (2018). Identifying off-target effects of etomoxir reveals that carnitine palmitoyltransferase I is essential for cancer cell proliferation independent of β-oxidation. Plos Biol. *16*, e2003782. https://doi.org/10.1371/journal.pbio.2003782.

56. Divakaruni, A.S., Hsieh, W.Y., Minarrieta, L., Duong, T.N., Kim, K.K.O., Desousa, B.R., Andreyev, A.Y., Bowman, C.E., Caradonna, K., Dranka, B.P., et al. (2018). Etomoxir Inhibits Macrophage Polarization by Disrupting CoA Homeostasis. Cel Metab. *28*, 490–503.e7. https://doi.org/10.1016/j.cmet.2018.06.001.

57. Lepropre, S., Kautbally, S., Octave, M., Ginion, A., Onselaer, M.B., Steinberg, G.R., Kemp, B.E., Hego, A., Wéra, O., Brouns, S., et al. (2018). AMPK-ACC signaling modulates platelet phospholipids and potentiates thrombus formation. Blood *132*, 1180–1192. https://doi.org/10.1182/blood-2018-02-831503.

58. Javidan, A., Jiang, W., Okuyama, M., Thiagarajan, D., Yang, L., Moorleghen, J.J., Muniappan, L., and Subramanian, V. (2019). miR-146a Deficiency Accelerates Hepatic Inflammation Without Influencing Diet-induced Obesity in Mice. Sci. Rep. *9*, 12626. https://doi.org/10.1038/s41598-019-49090-4.

59. Zhu, Y., Wang, Y., Li, Y., Li, Z., Kong, W., Zhao, X., Chen, S., Yan, L., Wang, L., Tong, Y., and Shao, H. (2023). Carnitine palmitoyltransferase 1A promotes mitochondrial fission by enhancing MFF succinylation in ovarian cancer. Commun. Biol. *6*, 618. https://doi.org/10.1038/s42003-023-04993-x.

60. Von Hundelshausen, P., and Weber, C. (2007). Platelets as immune cells: bridging inflammation and cardiovascular disease. Circ. Res. *100*, 27–40. https://doi.org/10.1161/01.RES.0000252802.25497.b7.

61. Jackson, S.P., Darbousset, R., and Schoenwaelder, S.M. (2019). Thromboinflammation: challenges of therapeutically targeting coagulation and other host defense mechanisms. Blood *133*, 906–918. https://doi.org/10.1182/blood-2018-11-882993.

62. Menéndez, R., Méndez, R., Aldás, I., Reyes, S., Gonzalez-Jimenez, P., España, P.P., Almirall, J., Alonso, R., Suescun, M., Martinez-Dolz, L., and Torres, A. (2019). Community-Acquired Pneumonia Patients at Risk for Early and Long-term Cardiovascular Events Are Identified by Cardiac Biomarkers. Chest *156*, 1080–1091. https://doi.org/10.1016/j.chest.2019.06.040.

63. Scheller, I., Beck, S., Göb, V., Gross, C., Neagoe, R.A.I., Aurbach, K., Bender, M., Stegner, D., Nagy, Z., and Nieswandt, B. (2022). Thymosin β4 is essential for thrombus formation by controlling the G-actin/F-actin equilibrium in platelets. Haematologica *107*, 2846–2858. https://doi.org/10.3324/haematol.2021.278537.

64. Ghergurovich, J.M., García-Cañaveras, J.C., Wang, J., Schmidt, E., Zhang, Z., TeSlaa, T., Patel, H., Chen, L., Britt, E.C., Piqueras-Nebot, M., et al. (2020). A small molecule G6PD inhibitor reveals immune dependence on pentose phosphate pathway. Nat. Chem. Biol. *16*, 731–739. https://doi.org/10.1038/s41589-020-0533-x.

65. Agrawal, S., Kumar, S., Sehgal, R., George, S., Gupta, R., Poddar, S., Jha, A., and Pathak, S. (2019). El-MAVEN: A Fast, Robust, and User-Friendly Mass Spectrometry Data Processing Engine for Metabolomics. Methods Mol. Biol. *1978*, 301–321. https://doi.org/10.1007/978-1-4939-9236-2_19.

66. Chen, L., Lu, W., Wang, L., Xing, X., Chen, Z., Teng, X., Zeng, X., Muscarella, A.D., Shen, Y., Cowan, A., et al. (2021). Metabolite discovery through global annotation of untargeted metabolomics data. Nat. Methods *18*, 1377–1385. https://doi.org/10.1038/s41592-021-01303-3.

YMTHE, Volume *34*

Supplemental Information

miR-146a mimic therapy protects against platelet-mediated thrombosis

Sonia Águila, Juan C. García-Cañaveras, Laura Zapata-Martínez, Ana B. Arroyo, Nuria García-Barberá, Salvador Carrillo-Tornel, Pedro J. Guijarro-Carrillo, Paula González-Jiménez, Ana Latorre, María L. Lozano, Bernard Payrastre, José Rivera, Marie-Pierre Gratacap, Raúl Méndez, Agustín Lahoz, Marcin Kortylewski, Rocío González-Conejero, and Constantino Martínez

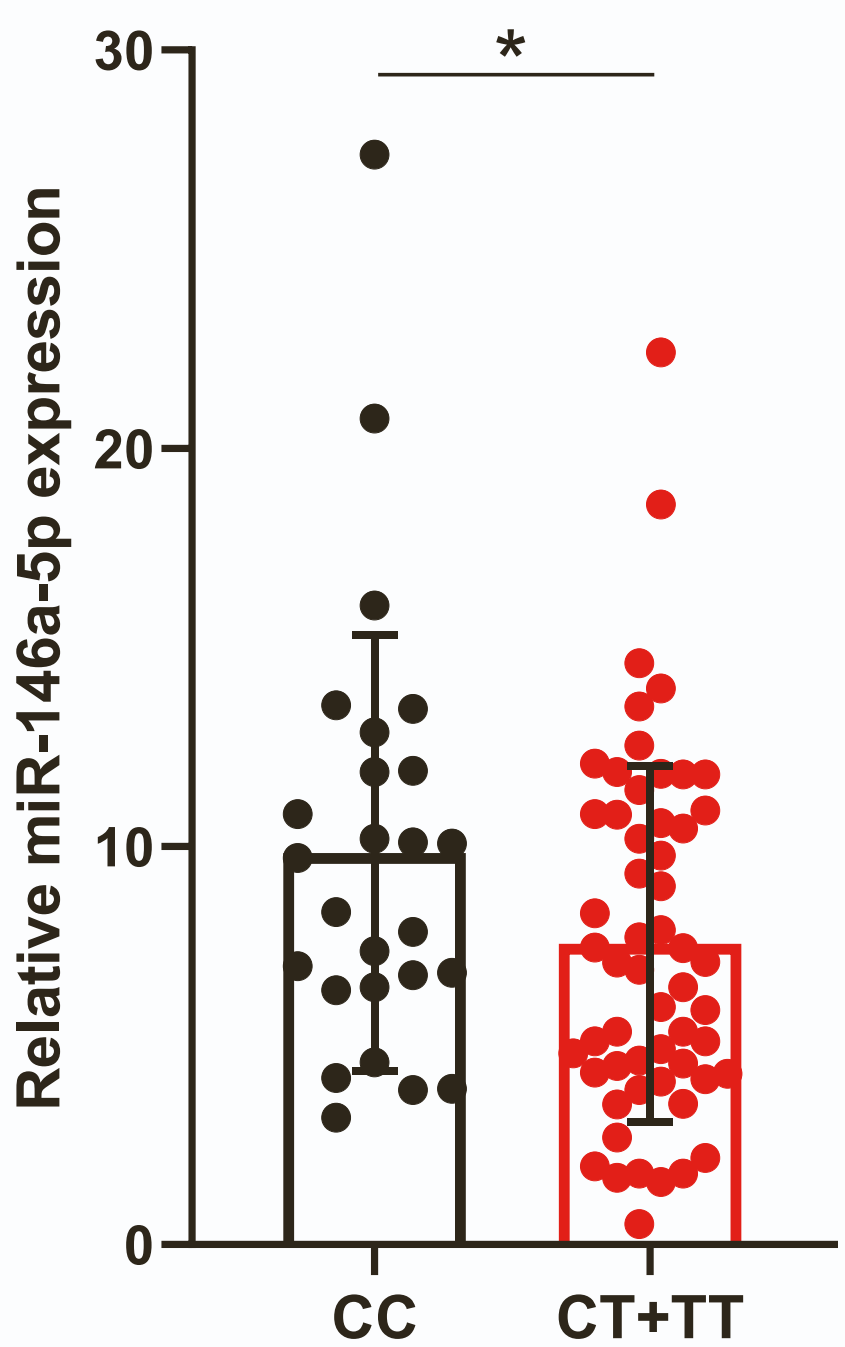

Figure S1. The presence of T-allele of rs2431697 decreased miR-146a in human platelets. RNA was extracted from washed platelets of 82 healthy donors' blood samples (CC=26, CT=26, TT=30). miR-146a were quantified by RT-qPCR. The statistical significance was calculated by Mann Whitney U test (*p<0.05).

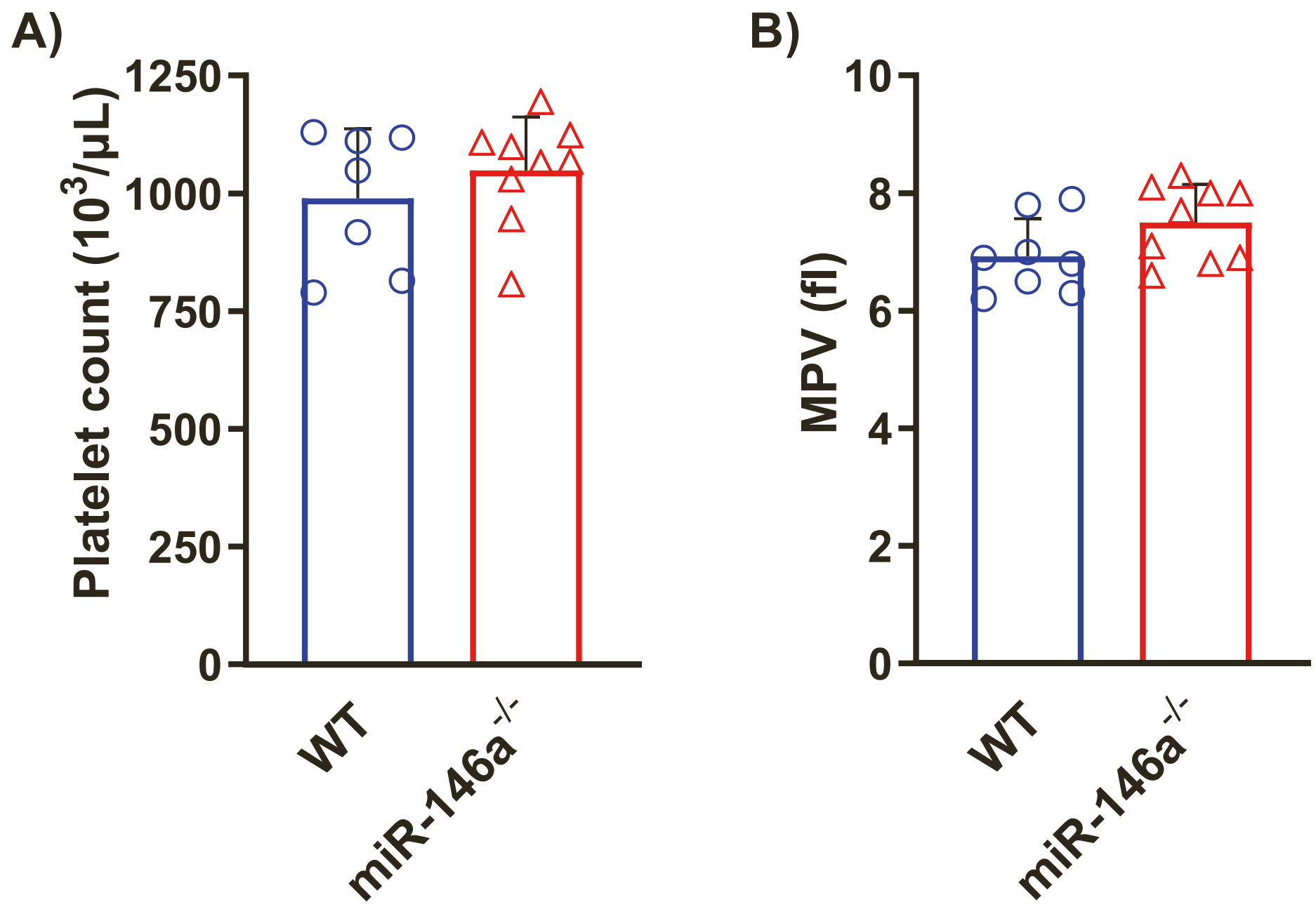

Figure S2. No significant differences in platelet count or volume between WT and miR-146a deficient mice. A) Platelet count and B) mean platelet volume (MPV) were measured (ADVIA 120 Hematology system) in 10 weeks-old mice (n=8 per group).

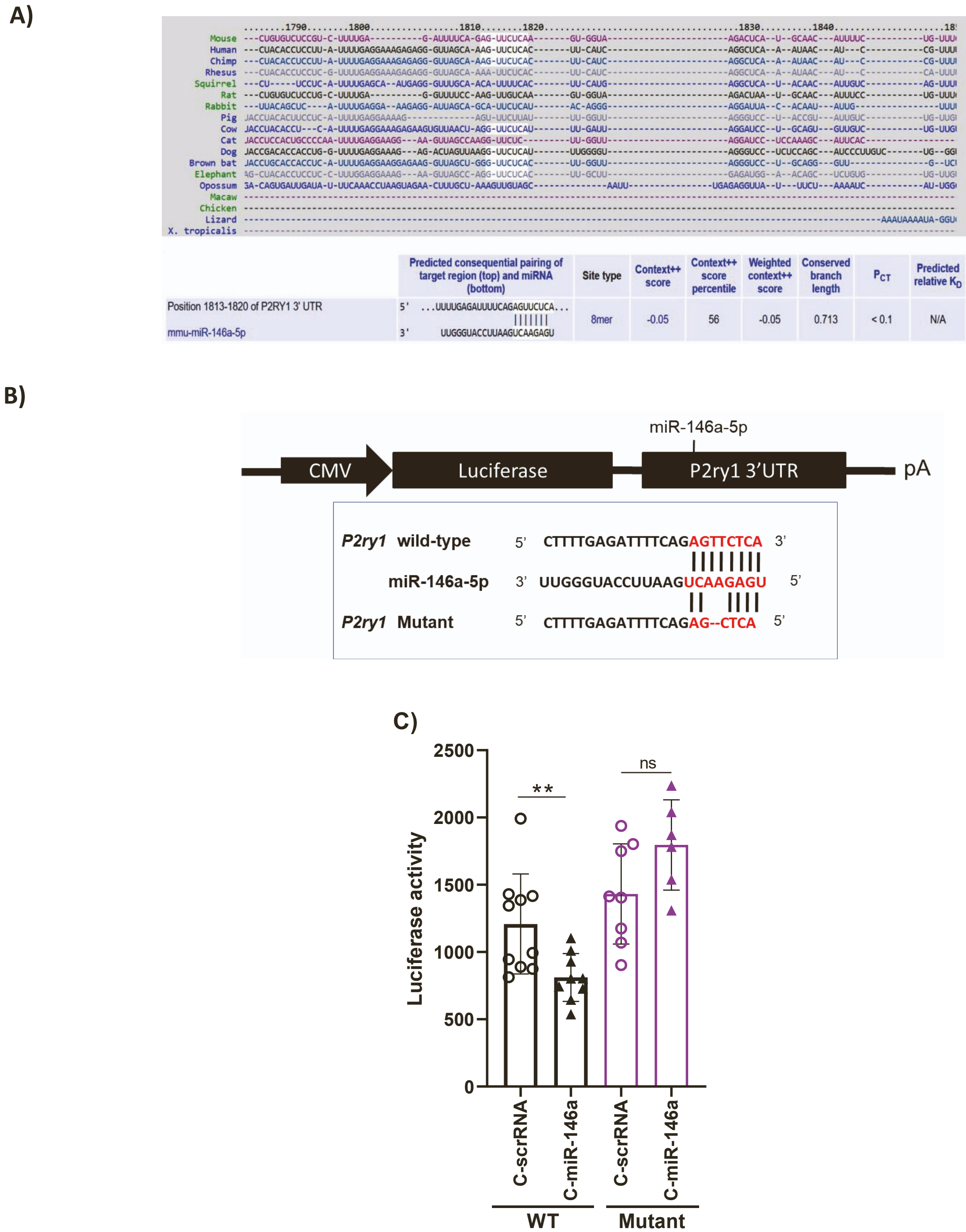

Figure S3. *P2ry1* gene is target of miR-146a. A) *P2ry1* mRNA is an *in-silico* target of miR-146a. TargetScanHuman software (https://www.targetscan.org/vert_80/) predicts that nucleotides (8mer) of 3'UTR region of *P2ry1* mRNA paired to miR-146a. B) Schematic diagram of the luciferase reporter plasmids containing a 390-nt fragment of the *P2ry1* wild-type (WT) 3'UTR with the predicted conserved miR-146a binding site, or a mutant *P2ry1* 3'UTR in which two nucleotides within the miR-146a-5p seed-matching region were deleted. C) Luciferase activity in HEK-293T cells co-transfected with the different vectors and a pRL-TK vector to normalize the data, and C-miR-146a mimic (C-miR-146a) or scramble RNA control (C-scrRNA) [n=2, 3-5 replicates/experiment]. The statistical significance was calculated by 2-tailed *t*-test (**p<0.01).

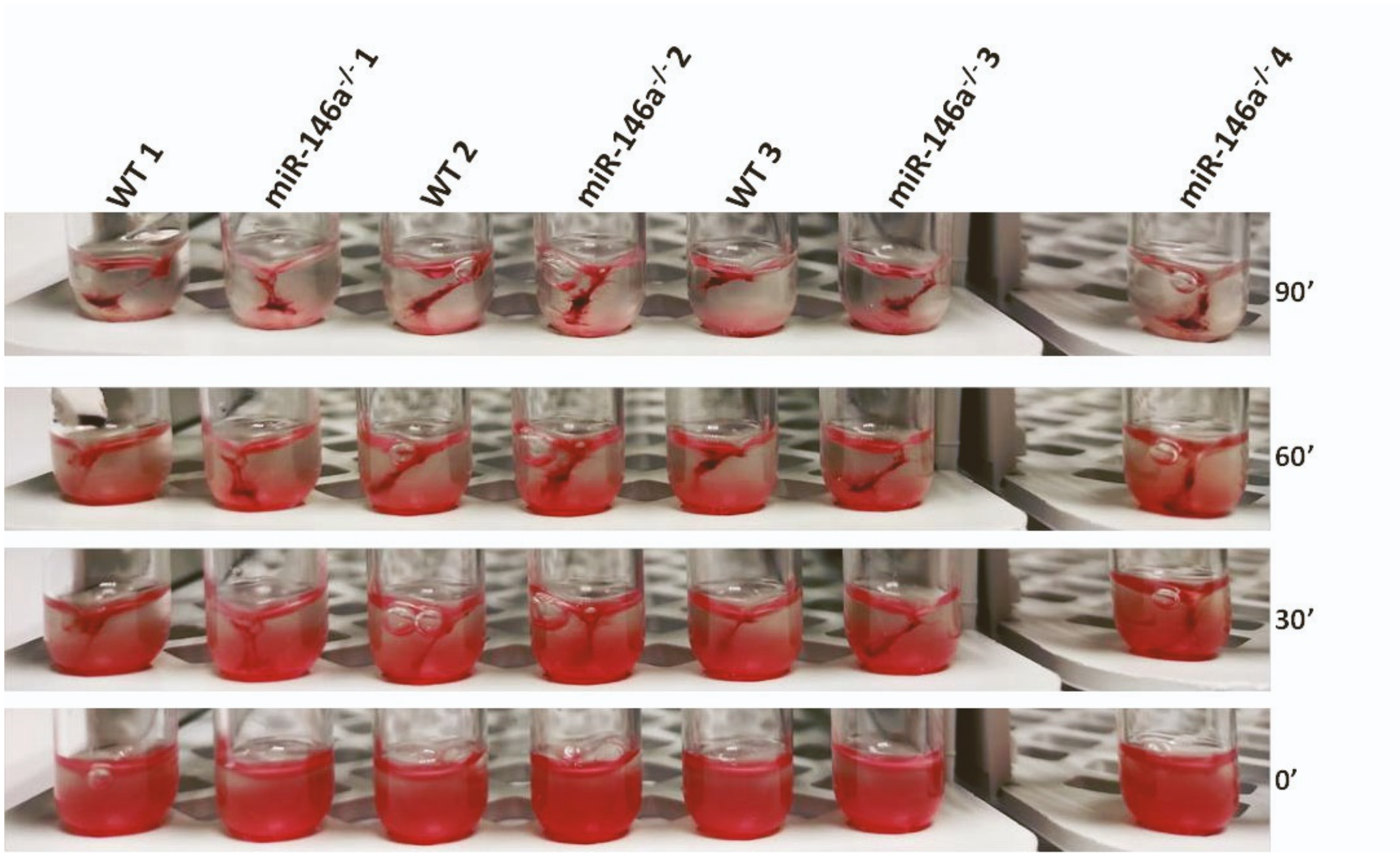

Figure S4. Clot retraction assay in WT *vs.* miR-146a$^{-/-}$ mice. $4x10^8$ washed platelets/mL were incubated in the presence of fibrinogen (2mg/mL) and thrombin (0.5 IU/mL) in Tyrode's buffer + $CaCl_2$ (2 mM) during 90 min.

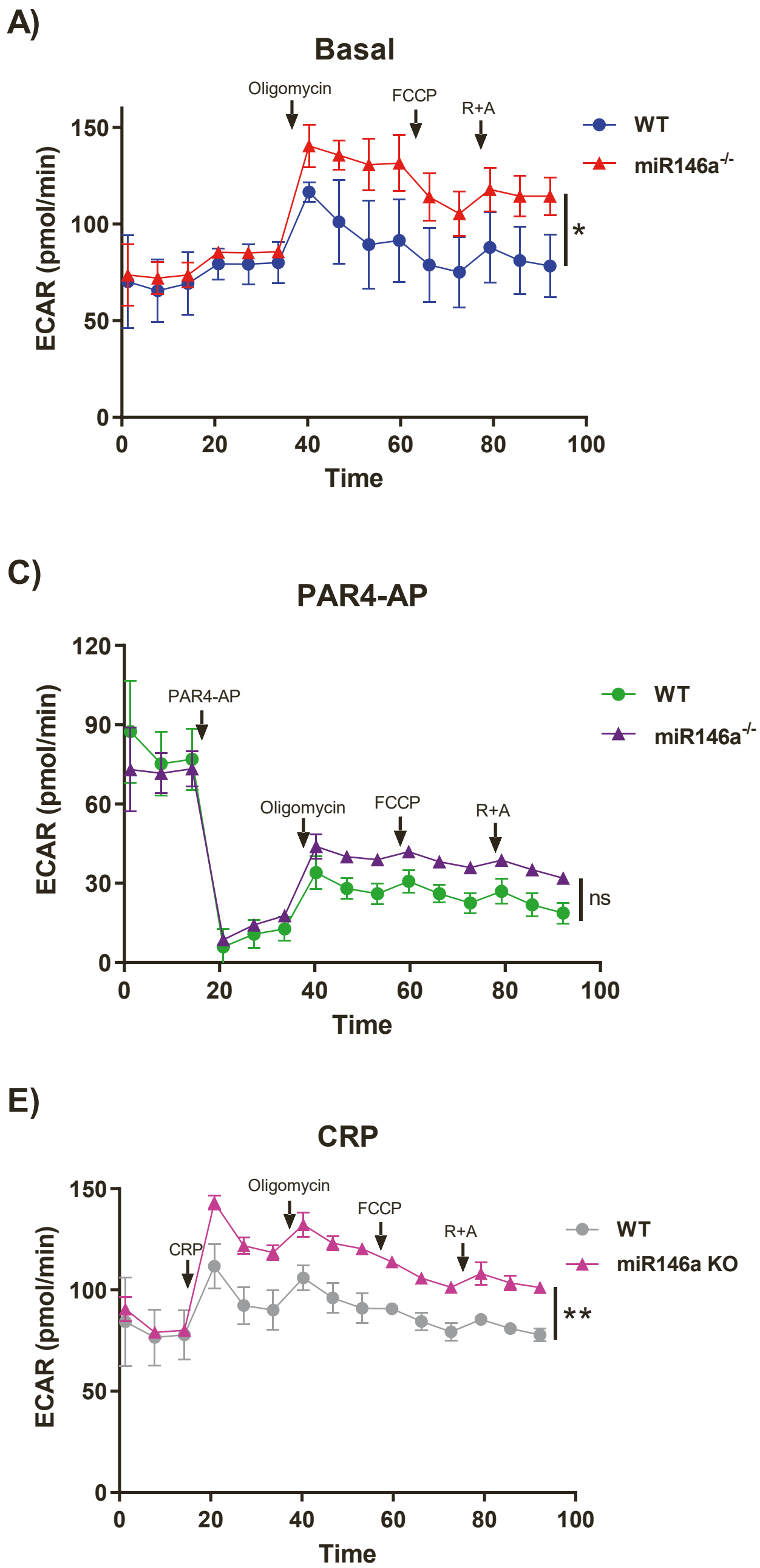

Figure S5. miR-146a$^{-/-}$ platelet showed elevated ECAR in resting and stimulated conditions. Extracellular acidification rate (ECAR) was determined by Seahorse Analyzer using Seahorse Cell Mito Stress Test kit at A) basal and (B-E) activated state with B) Thrombin (0.5IU/mL), C) PAR4-AP (500µM), D) PMA (30nM), E) CRP (10µg/mL) (pool of 6 mice/group, mean ± SD). The area under the curve (AUC) and its significance were calculated by 2-tailed *t*-test (*p<0.05, **p<0.01, ns=non-significant).

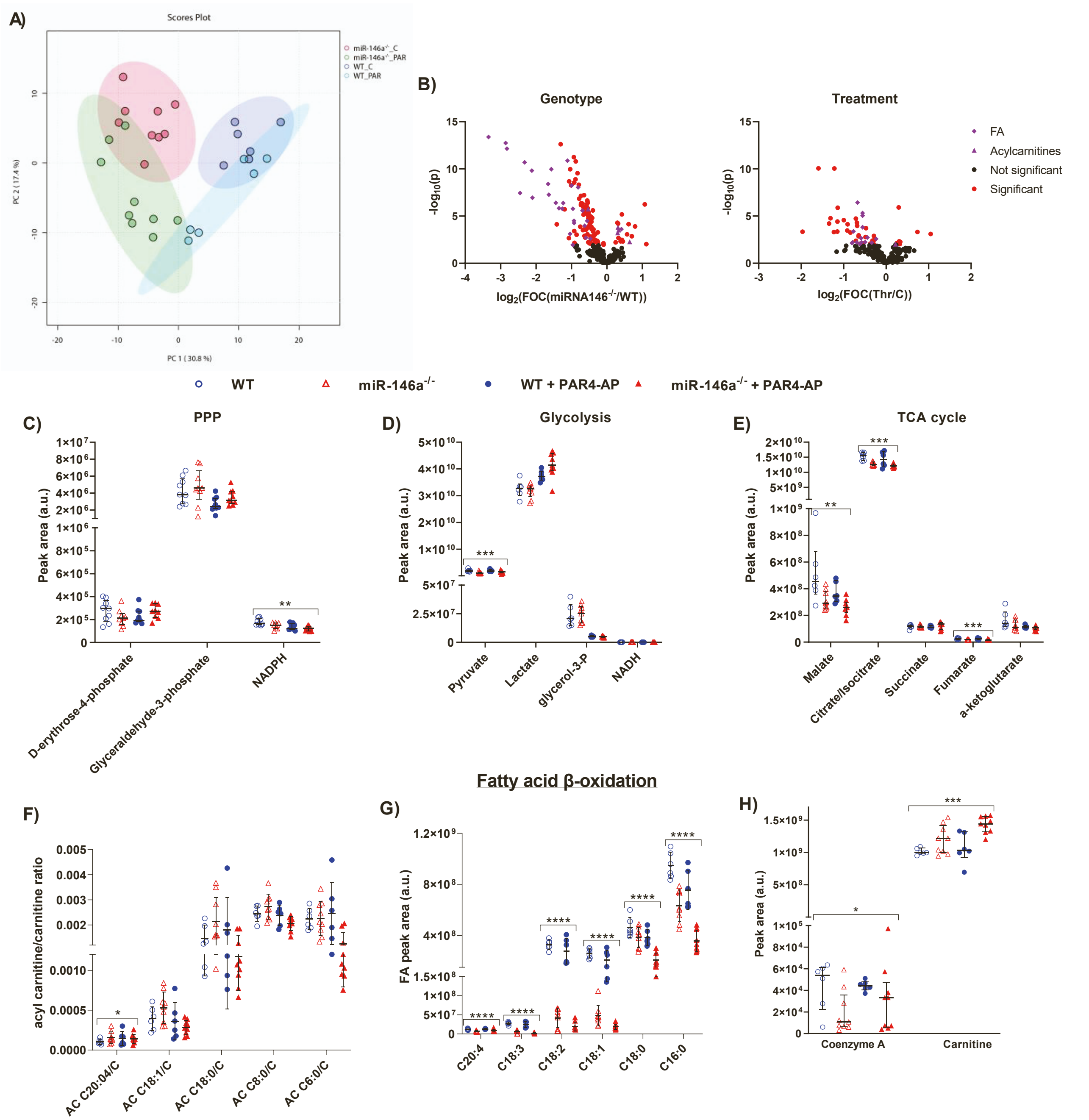

Figure S6. Metabolomics showed enhanced fatty acid β-oxidation (FAO) for miR-146a deficient platelets ± PAR4-AP. Untargeted metabolomics was performed in WT *vs*. miR-146a$^{-/-}$ platelets ± agonist 500µM PAR4-AP by Q Exactive Plus Hybrid Quadrupole-Orbitrap mass spectrometer (2-3 pools of 3 mice/group and technical triplicated). A) Principal component analysis (PCA), B) volcano plot of platelet metabolome. Main intermediates or products of C) pentose phosphate pathway (PPP), D) glycolysis, E) tricarboxylic acid cycle (TCA), F) acylcarnitines/carnitines (AC/C) ratio, G) fatty acid levels, H) coenzyme A and carnitines levels in resting or stimulated (Thr) platelets. The statistical significance was calculated by 2-way ANOVA *p<0.05; **p<0.01; ***p<0.001; ****p<0.0001, only the genotype statistical significance was represented in the graphs.

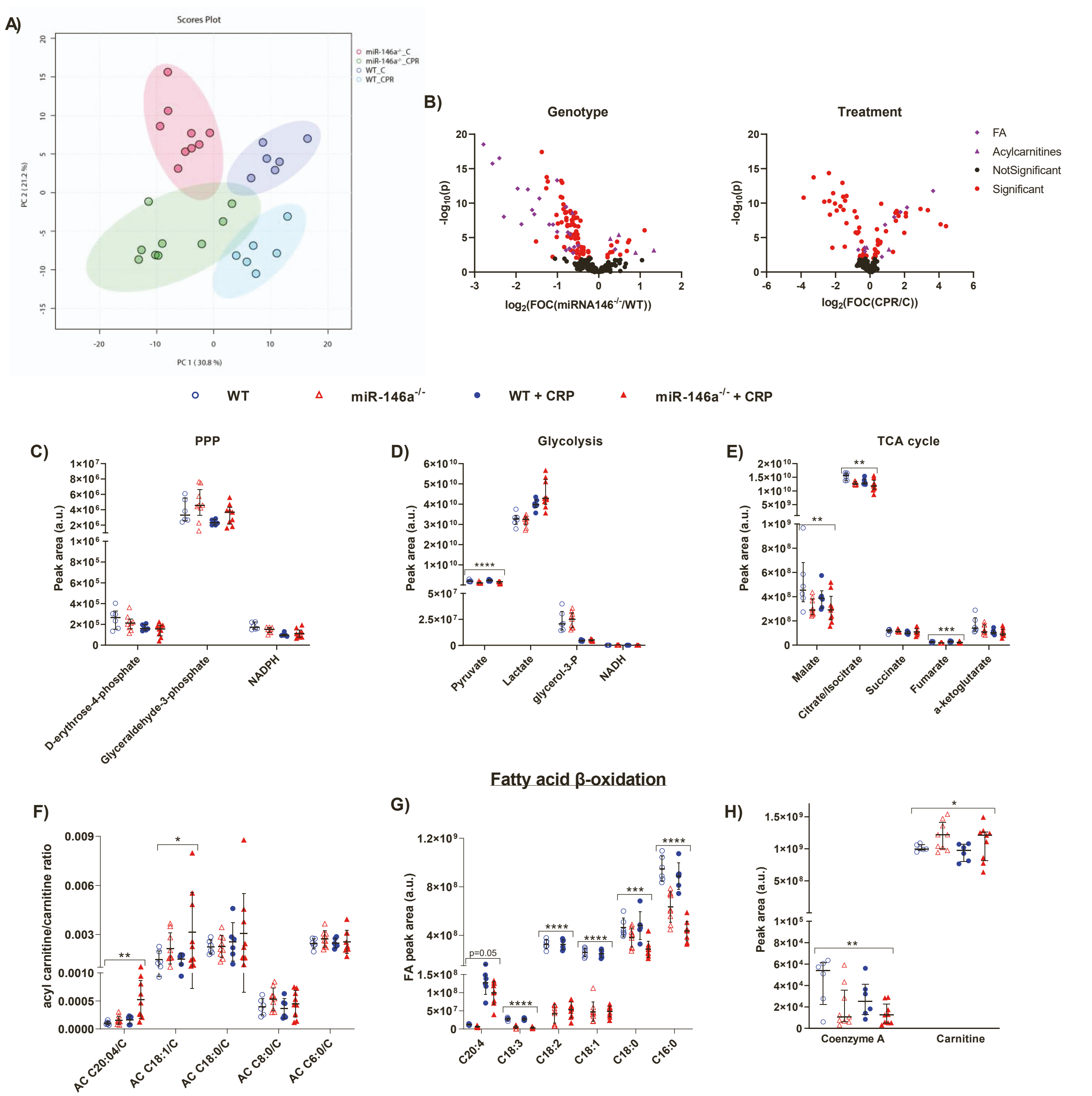

Figure S7. Metabolomics showed enhanced fatty acid β-oxidation for miR-146a deficient platelets ± CRP. Untargeted metabolomics was performed in WT *vs*. miR-146a$^{-/-}$ platelets ± agonist 10µg/mL CRP by Q Exactive Plus Hybrid Quadrupole-Orbitrap mass spectrometer (2-3 pools of 3 mice/group and technical triplicated). A) Principal component analysis (PCA), B) volcano plot of platelet metabolome. Main intermediates or products of C) pentose phosphate pathway (PPP), D) glycolysis and E) tricarboxylic acid cycle (TCA), F) acylcarnitines/carnitines (AC/C) ratio, G) fatty acid levels, H) coenzyme A and carnitines levels in resting or stimulated (Thr) platelets. The statistical significance was calculated by 2-way ANOVA *p<0.05; **p<0.01; ***p<0.001; ****p<0.0001, only the genotype statistical significance was represented in the graphs.

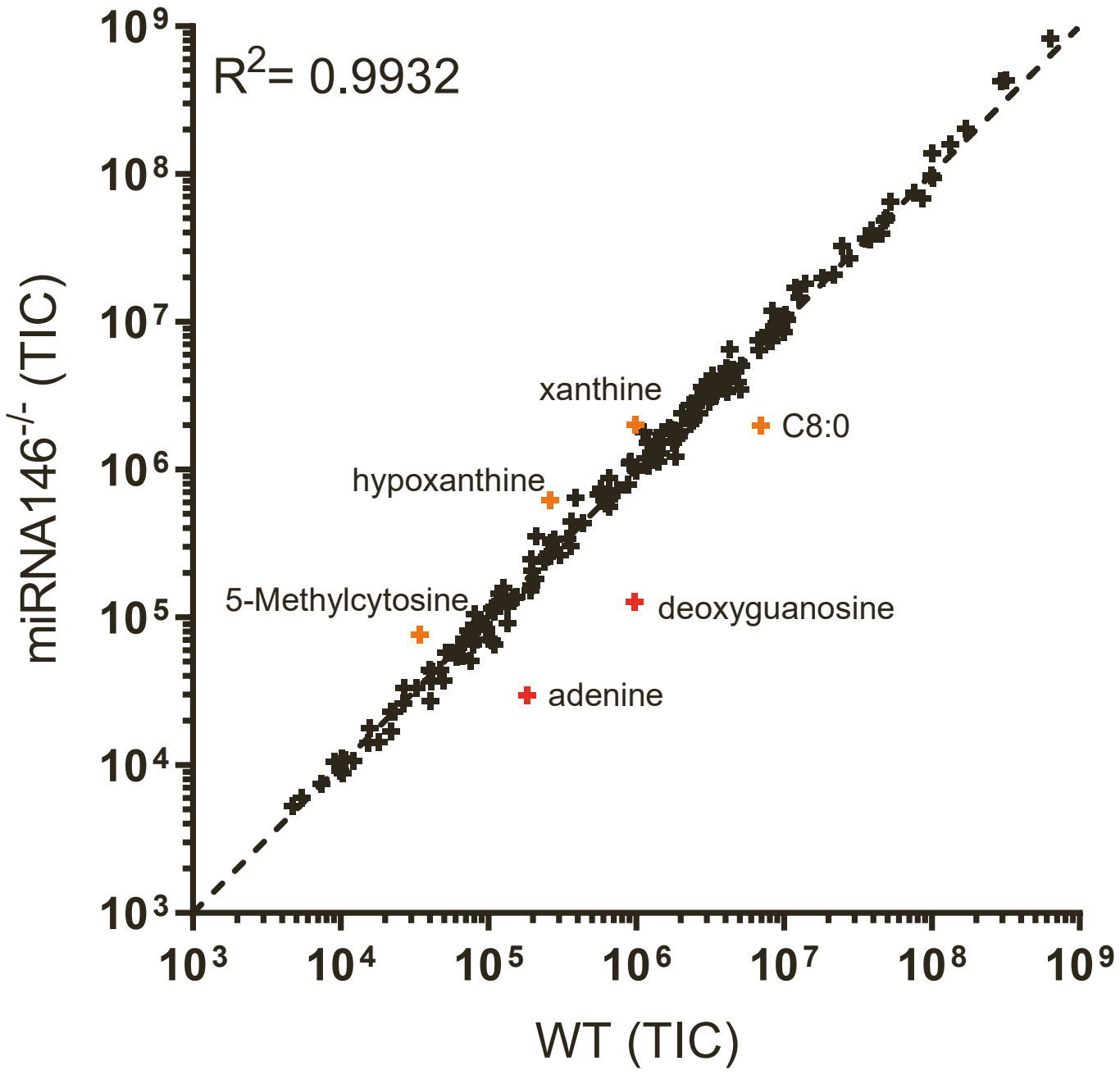

Figure S8. Serum metabolome profiles from WT and miR-146a$^{-/-}$ mice were similar. Untargeted metabolomics was performed in WT *vs.* miR-146a$^{-/-}$ serum by Quadrupole-Q Exactive Orbitrap mass spectrometer (mean, n = 6/group**).** Metabolites whose abundances differ by more than four-fold between conditions are highlighted in red, metabolites whose abundances differ by more than two-fold between conditions are highlighted in orange.

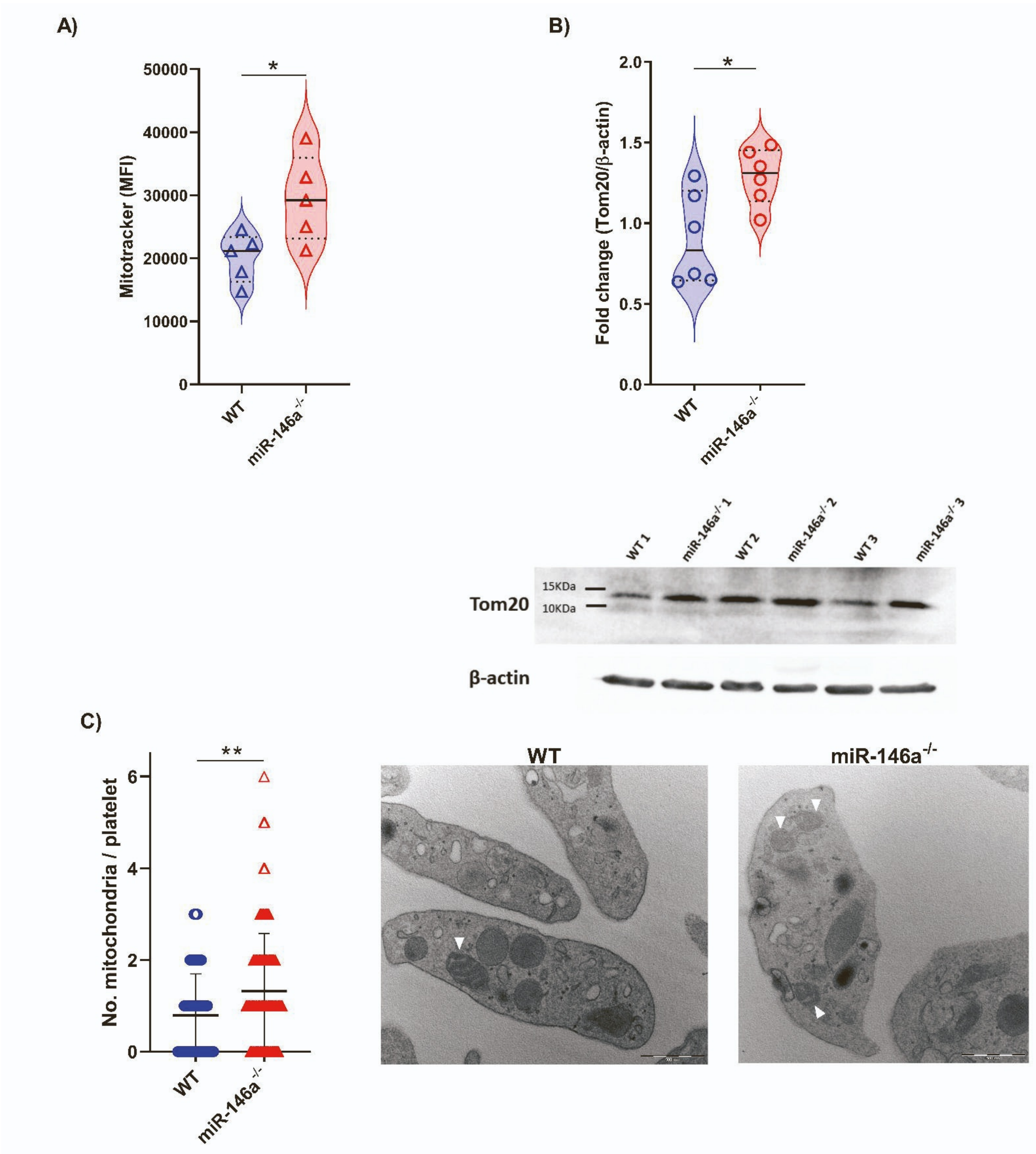

Figure S9. miR-146a deficiency increased mitochondrial mass in platelets. A) Mitotracker green (100nM) determination by flow cytometry, B) Tom20 detection by western blot in WT *vs*. miR-146a$^{-/-}$ resting platelets (a representative blot is shown) and the densitometry Tom20/β-actin was calculated. The statistical analyses were performed by 2-tailed *t*-test (*p<0.05, n=5-6 mice/group). C) Transmission electron microscopy was performed on miR-146a-deficient platelets *vs.* WT. The number of mitochondria per platelet was quantified for 40–50 platelets per mouse in randomly selected images. Scale bar: 500 nm. The Mann-Whitney U test was used for statistical analysis (**p<0.01).

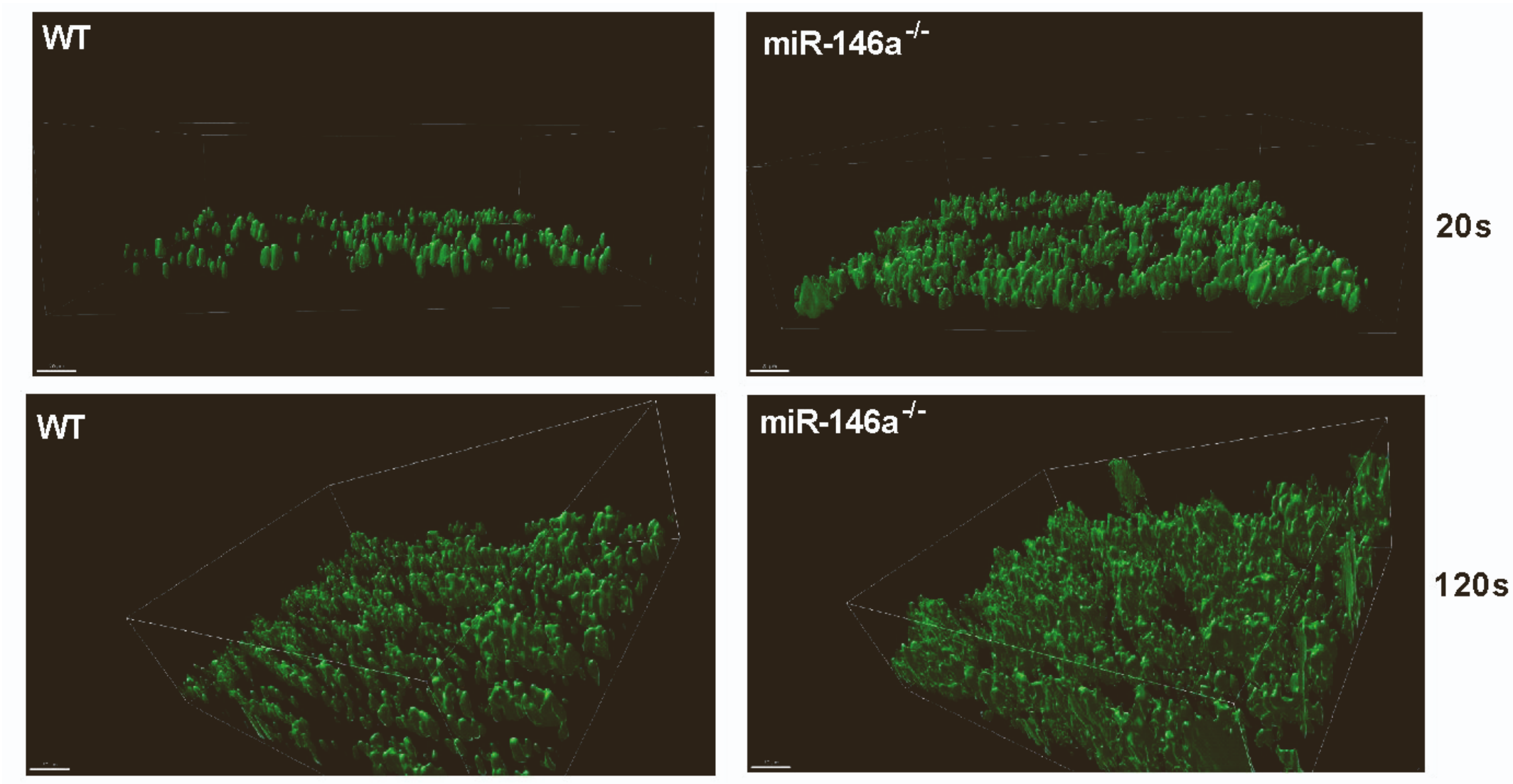

Figure S10. miR-146a$^{-/-}$ platelet hyperreactivity under flow. Whole blood from WT and miR-146$^{-/-}$ mice was perfused over collagen-coated microcapillary at a shear rate of 1100 s^{-1}. Platelets were labeled with $DiOC_6$, and thrombi volumes (μm^3) were calculated using Imaris software. Representative images of 3D aggregates formation at 20s and 120s are shown. Scale bar: 20µm.

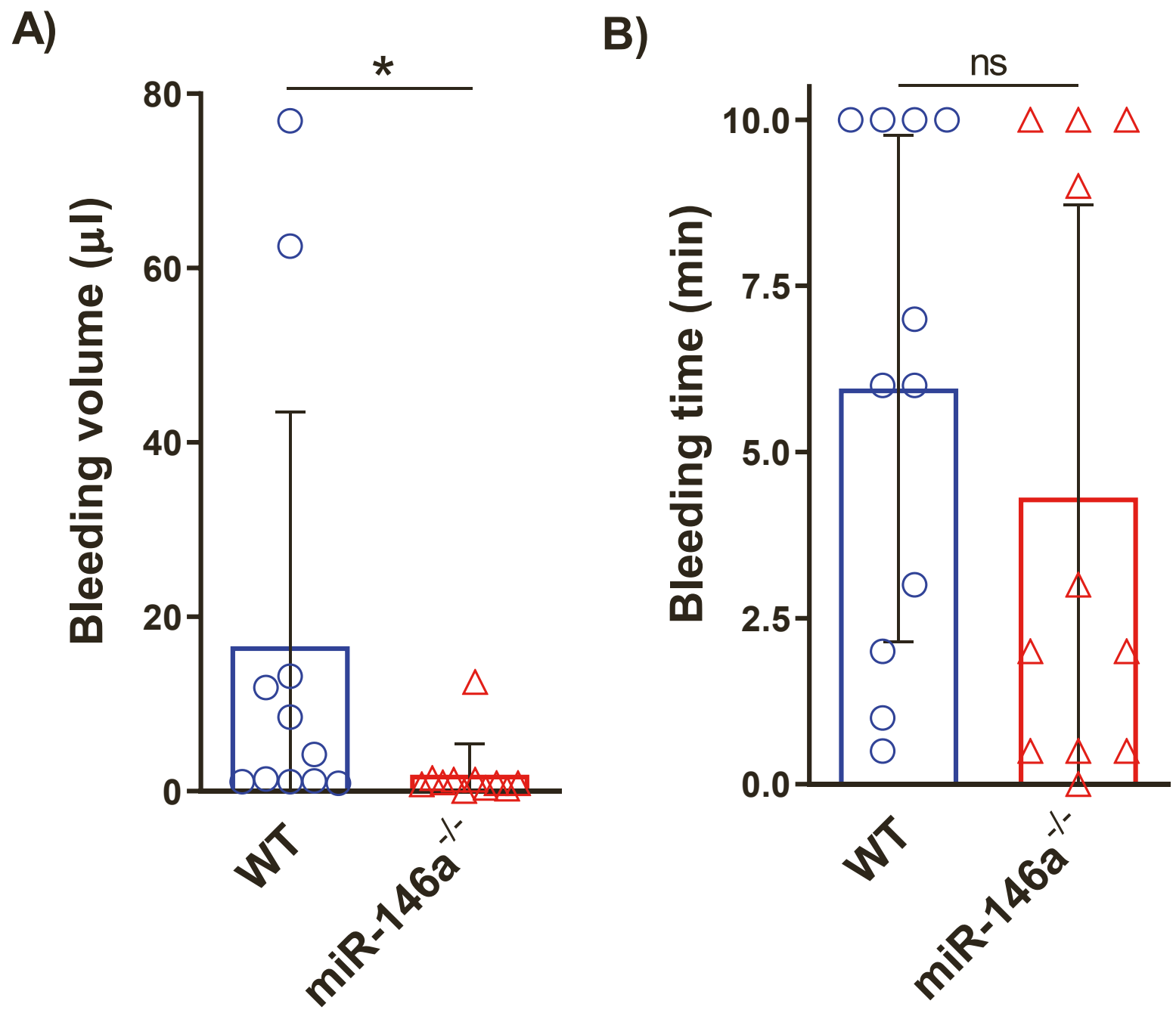

Figure S11. miR-146a deficient mice had reduced bleeding than WT mice. A) Bleeding volume was determined by analyzing the hemoglobin content of blood collected in saline tubes (n=11/group). B) Bleeding was monitored for a maximum of 10 min. The statistical significance was calculated by Mann-Whitney test (*p<0.05, ns=non-significant).

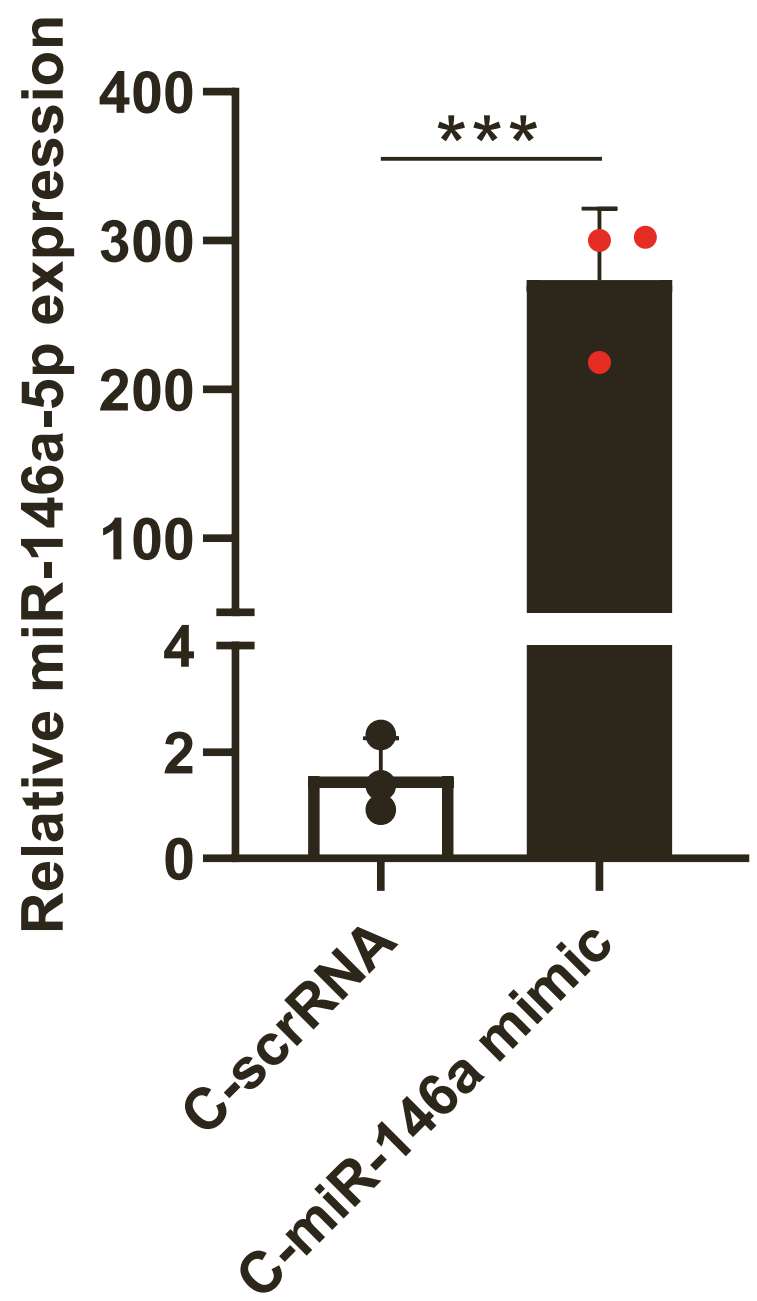

Figure S12. C-miR-146a mimic administration increased 213-fold miR-146a levels in platelets. C-miR-146a mimic and C-scrRNA as a control were intravenously injected for 3 days (10mg/kg). WT mice blood was collected from the cava vein, platelets were washed and then RT-qPCR for miR-146a and U6 was performed. The statistical significance was calculated by 2-tailed *t*-test (***p<0.001).

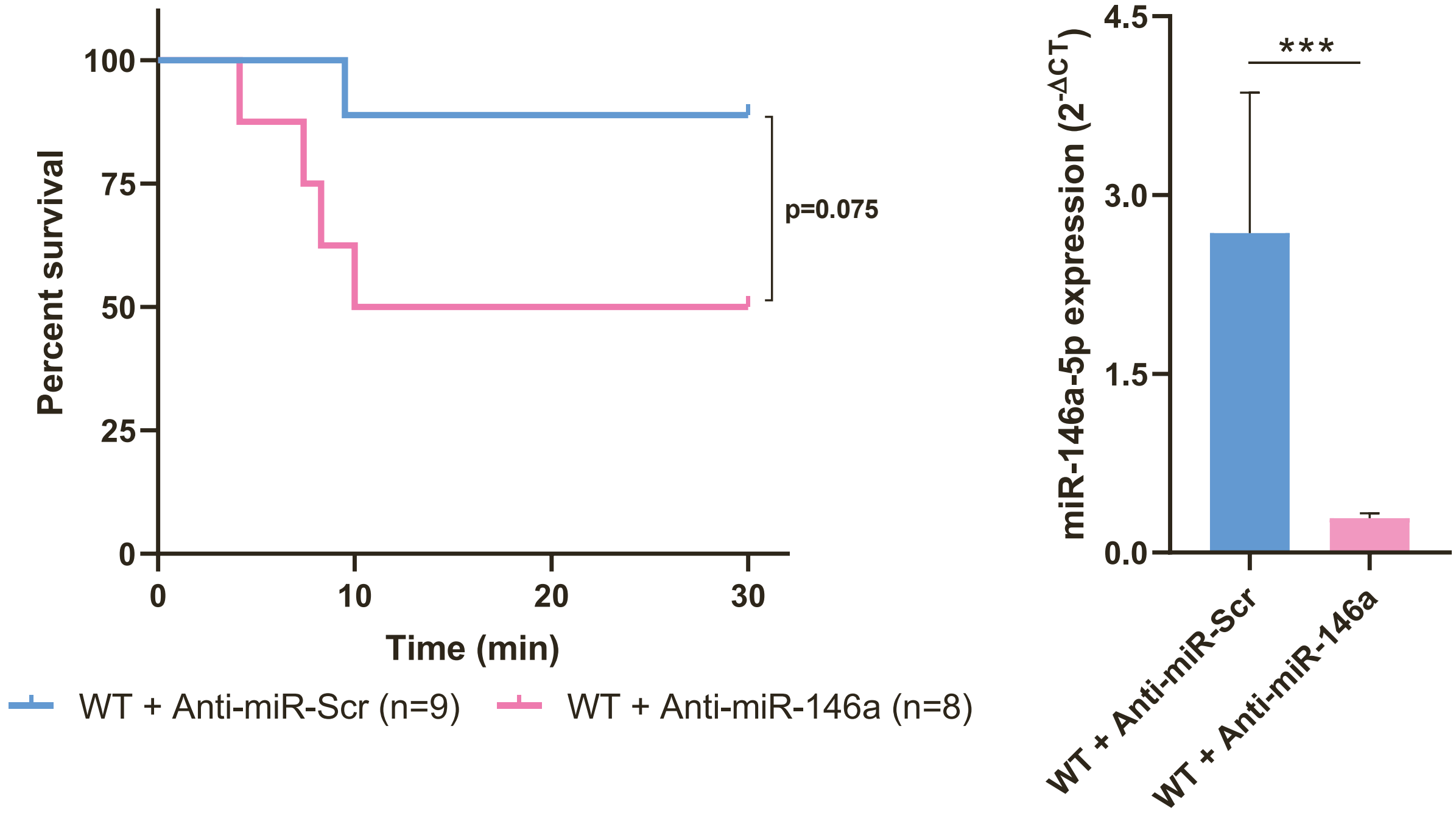

Figure S13. Anti-miR-146a administration decreased miR-146a levels in platelets and impaired the survival of WT mice after systemic thrombosis (PE). AntimiR-146a (n=8) and scrRNA (n=9) as a control were intravenously injected for 2 days (5 mg/Kg). WT mice blood was collected, platelets were washed and then RT-qPCR for miR-146a and U6 was performed. The statistical significance was calculated by Log rank Mantel-Cox test and by 2-tailed *t*-test (***p<0.01), respectively.

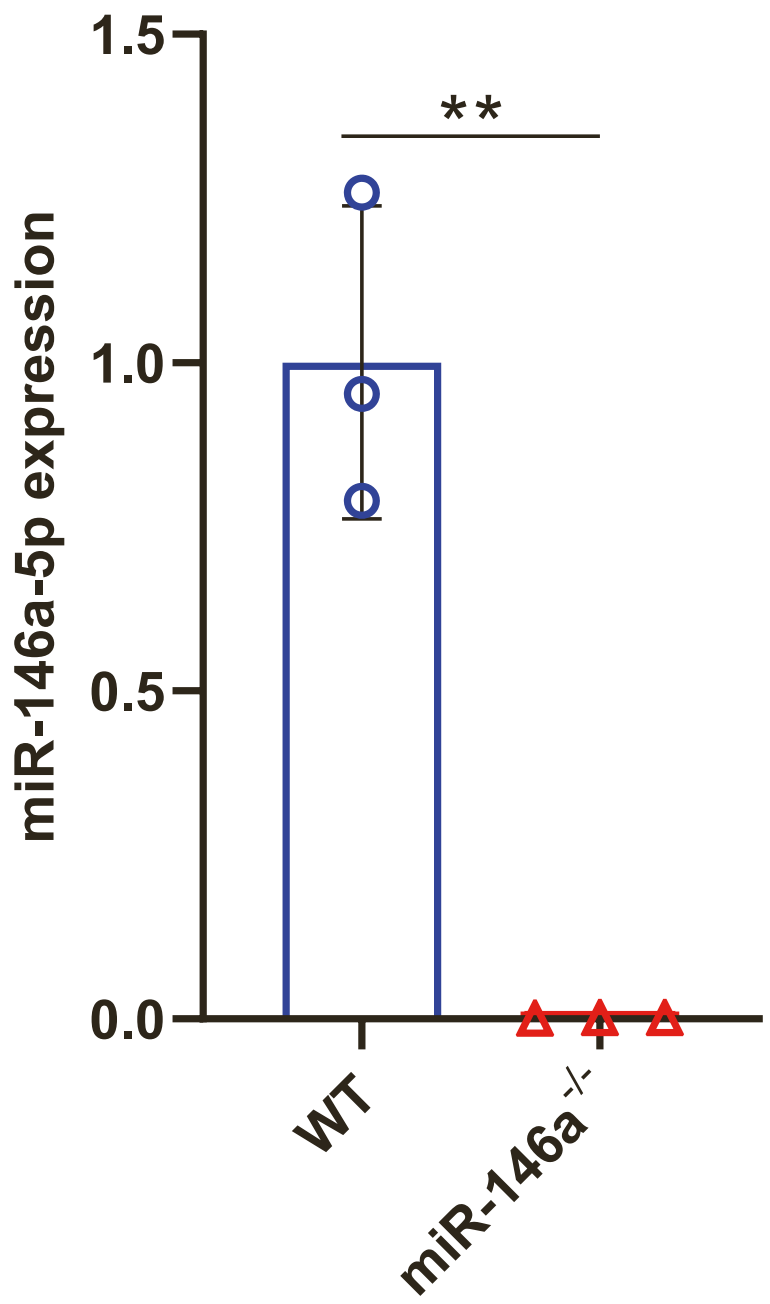

Figure S14. miR-146a levels in platelets from WT and miR-146a deficient mice. administration decreased 9.28-fold miR-146a levels in platelets. WT and miR-146a mice blood was collected, platelets were washed and then RT-qPCR for miR-146a and U6 was performed. The statistical significance was calculated by 2-tailed *t*-test (**p<0.01).

Table S1. Clinical features healthy controls

	rs2431697		
	**CC (n=11)**	**TT (n=10)**	**p-value**
**Age (years)**	41.2 ± 15.1	44.3 ± 12.8	0.6
**Platelets (x10^3/µl)**	237 ± 49	275 ± 79	0.2
**MPV (fl)**	10.9 ± 1.3	10.7 ± 1.1	0.7
**Hb (g/dl)**	14.0 ± 1.2	14.4 ± 2.2	0.6
**Hematocrit (%)**	42.2 ± 3.1	43.2 ± 5.1	0.6
**Neutrophils (x10^3/µl)**	4.0 ± 1.4	3.7 ± 1.2	0.5
**Monocytes (x10^3/µl)**	2.2 ± 0.8	2.0 ± 0.6	0.3
**Lymphocytes (x10^3/µl)**	0.7 ± 0.2	0.6 ± 0.1	0.5

Media ± SD from healthy blood donors homozygous for C or T allele of rs2431697. Their blood samples collected in BAPA or citrate tubes were used to perform T-TAS and platelet activation assays, respectively. P-values were calculated using 2-tailed *t*-test.

Video S1 legend

Microfluidics assay of WT and miR-146a-/- platelets. Mouse blood from WT and miR-146a-/- mice incubated with DiOC6 (2 µM) to label platelets was perfused into the collagen-coated microchannels at 1,100s^{-1}. Platelet adhesion and thrombus formation were visualized and recorded using a fluorescence microscope (Leica Microsystems).
